# Olefin Metathesis in Water: Speciation of a Leading Water-Soluble Catalyst Pinpoints Challenges and Opportunities for Chemical Biology

**DOI:** 10.1021/jacs.4c16700

**Published:** 2025-03-07

**Authors:** Christian O. Blanco, Samantha K. Cormier, Angus J. Koller, Eszter Boros, Deryn E. Fogg

**Affiliations:** † Center for Catalysis Research and Innovation, and Department of Chemistry and Biomolecular Sciences, University of Ottawa, Ottawa, Ontario K1N 6N5, Canada; ‡ Department of Chemistry, University of Southern Maine, Portland, Maine 04103, United States; § Department of Chemistry, University of Wisconsin−Madison, Madison, Wisconsin 53706, United States; ∥ Department of Chemistry, University of Bergen, N-5007 Bergen, Norway

## Abstract

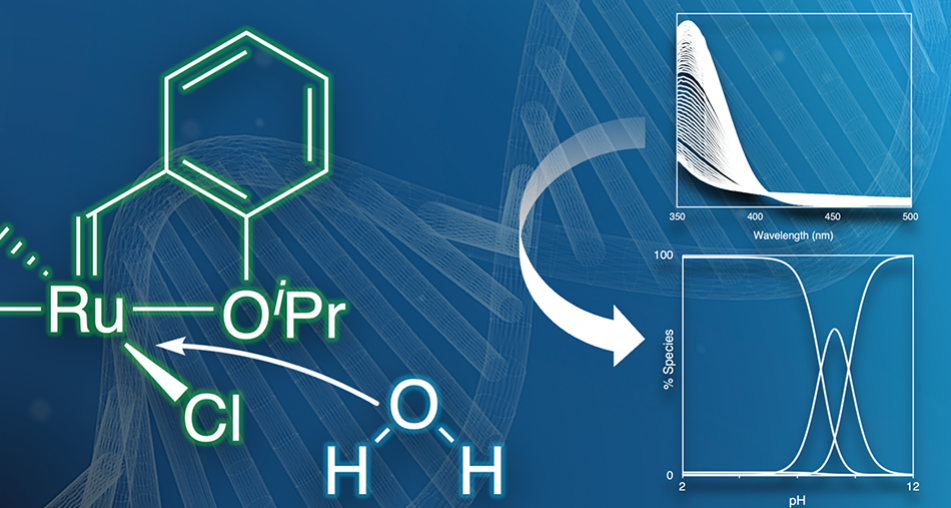

The metathetical modification of biomolecules in aqueous environments holds great promise for advances at the interface of chemistry, biology, and medicine. However, rapid degradation of the metathesis catalysts necessitates their use in large stoichiometric excess, resulting in undesired side-reactions promoted by the ruthenium products. Although water is now known to play a central role in catalyst decomposition, the elusive nature of the intermediates has hampered insight into the pathways involved. We describe the detailed speciation in water of AquaMet (**AM**), the dominant ruthenium catalyst used for aqueous metathesis, and implications for catalysis. Potentiometric and spectroscopic speciation studies reveal that only trace **AM** is present under the pH-neutral, salt-free conditions routinely employed in synthetic applications of aqueous metathesis. Instead, metathesis-inactive hydroxide species dominate. Even at pH 3, Ru–H_2_O complexes dominate in 0.01 M NaCl_(aq)_, and the water ligands are readily deprotonated as the pH is increased. Raising NaCl_(aq)_ concentrations to 1 M suppresses deprotonation events below pH 8, stabilizing **AM** as the dominant solution species at neutral pH, and significantly expanding the metathesis-compatible regime. Hitherto unrecognized catalyst solubility issues are also revealed, pointing toward avenues for advance. More broadly, the capacity to directly link catalyst environment to structure and performance opens new opportunities for olefin metathesis in complex, water-rich settings.

## Introduction

A tension at the heart of bioorthogonal catalysis lies in the nature of the reaction medium. The challenge of catalyst degradation in biological environments is well recognized.^1^ Reactive sites on biomolecules represent one clear hazard: arguably even more problematic are cases where water, the native solvent of chemical biology,^2^ is inimical to catalyst stability. Speciation studies offer critical opportunities to identify and intercept deleterious pathways.^3,4,5^ Within the context of olefin metathesis, such studies have as-yet unexplored potential to enable advance at an interdisciplinary frontier.

**Scheme 1:**
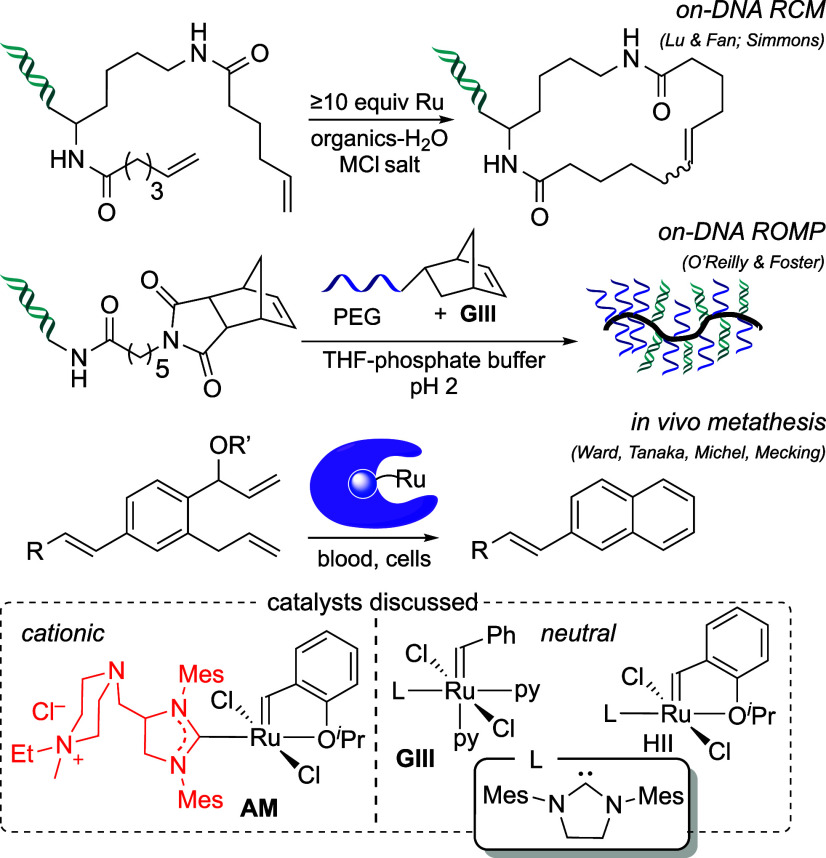
Frontier Applications in Aqueous Metathesis

Among bioorthogonal organometallic reactions, olefin metathesis stands out for its capacity to forge flexible, sterically minimal linkages.^6^ Ruthenium-promoted metathesis offers unique opportunities, owing to the greater water-tolerance of this second-row platinum-group metal, relative to metals earlier or higher in the transition-metal series.^7^ Prominent applications depicted in Scheme 1 include ring-closing metathesis (RCM) in vivo, in cells and in blood,^7a−7f^ on-DNA RCM,^7f,7g^ and ring-opening metathesis polymerization (ROMP) of DNA macromonomers in water-rich media.^7h^ Even for the ruthenium catalysts, however, decomposition competes with metathesis. *Catalytic* modification of biomolecules in water hence remains rare,^6^ despite the myriad of water-soluble metathesis catalysts available. Among these, commercial AquaMet^8,9^ (**AM**, Scheme 1) is most widely used, typically for model ‘green chemistry’ reactions.^10^**AM** has met with limited success in chemical biology,^7b,7d,7f,11^ owing to facile decomposition and side-reactions promoted by spent catalyst. Of the latter, olefin migration is most typical,^11e^ but DNA degradation represents a limitation in major areas of opportunity, including production of DNA-encoded chemical libraries.^7f^

Water itself is now recognized as a key contributor to degradation of ruthenium metathesis catalysts.^12,13,14^ This constitutes a core challenge for bioorthogonal metathesis. While the Ru species involved remain conjectural, an important advance by the Matsuo and O’Reilly groups established that chloride salts can significantly improve metathesis yields.^12a,12b^ Understanding the changes that take place in water is essential to design high-performing catalysts for chemical biology, and to enable informed use of existing catalysts. Here we establish the speciation of a leading water-soluble metathesis catalyst in water. We identify the distribution and equilibria between chloride, aqua, and hydroxide species, and demonstrate that this information can be used to identify conditions that stabilize the desired, productive catalyst species over a broad pH range. Such insight is essential to accurately predict catalyst performance in aqueous environments, and to enable challenging applications in chemical biology.

## Results and Discussion

### Impact of Water on Productivity of AquaMet (AM) in Metathesis

**Scheme 2:**
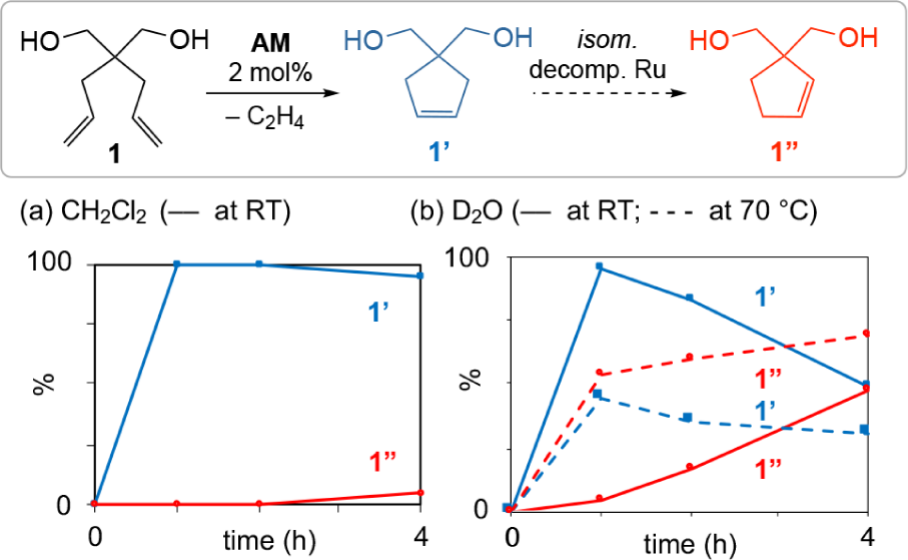
Impact of Water on RCM Rates and Selectivity The rate profile at 70 °C in D_2_O is reproduced from ref (11a).

To demonstrate that catalyst decomposition is relevant even in metathesis of undemanding substrates, we undertook probe experiments with the commercially available catalyst **AM**, which currently dominates applications in aqueous metathesis.^6d^ The impact of water on performance was examined for a benchmark metathesis reaction,^6d^ RCM of water-soluble diol **1**. As shown in Scheme 2a, cyclization of **1** is complete within 1 h at RT in CH_2_Cl_2_ (in which **AM** is fully soluble). Selectivity for the expected RCM product **1’** is complete, indicating that RCM is fast relative to catalyst decomposition. Ensuing positional isomerization of the C=C bond slowly generates **1″** in minor amounts (ca. 5% after 4 h). For the corresponding reaction in D_2_O, in contrast, isomerization is detectable even at 1 h at RT; by 4 h, it accounts for ca. 50% of the product distribution (Scheme 2b). Isomerization is significantly more aggressive at elevated temperatures, reaching 60% after 1 h at 70 °C. Water clearly limits the lifetime, productivity, and selectivity of **AM**. Moreover, isomerization appears even more problematic in water than in organic solvents.

### Insight into Speciation in Water: NMR vs UV–vis Spectroscopy

**Figure 1:**
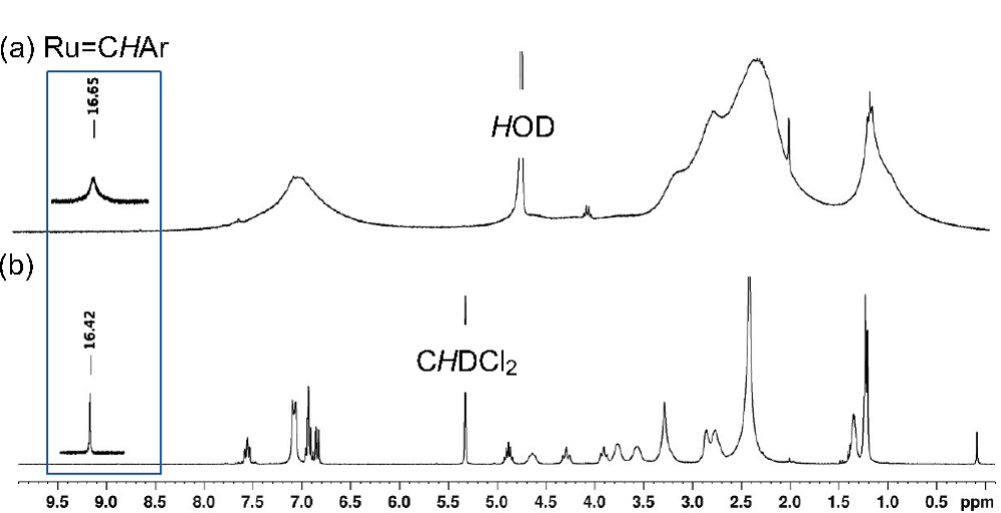
Dynamic behavior of **AM** in water limits the utility of ^1^H NMR analysis. (a) Extreme line-broadening in D_2_O. (b) Contrasting sharp signals in anhydrous CD_2_Cl_2_. Insets show the alkylidene signal.

In organic solvents, catalyst decomposition proceeds via elimination of the metallacyclobutane (MCB) and [Ru]=CHR ligands, as we demonstrated in prior mechanistic studies.^15,16^ Quantitative detection of their organic degradation products was hence invaluable in identifying the primary decomposition event. In water, this approach is less useful. While the fate of the metathesis-active ligands can afford insight into later stages in the decomposition cascade (see below), it does not report on the initial elementary steps involving aquation (i.e., exchange of chloride for water) and ensuing deprotonation of the aqua ligands. A further complication stems from rapid equilibration of multiple solution species on the NMR timescale. The associated exchange broadening (compare Figure 1a vs 1b) precludes structure elucidation and assignment by ^1^H NMR analysis.^17^

To gain insight into the early stages of the reaction, we turned to UV–vis analysis as a complementary tool. The viability of spectrophotometric analysis for insight into the ruthenium species formed is suggested by the color changes observed for the bulk solution: from green to yellow over 8 h, orange over 24 h, and red-brown after 48 h. These changes reflect the distinctive electronic structures of the [Ru]=CHR species (including **AM** and its aqua or hydroxo derivatives), and their Ru(II) decomposition products.

### Establishing the Spectrophotometric Signature for Dichloride Complex AM

**Figure 2:**
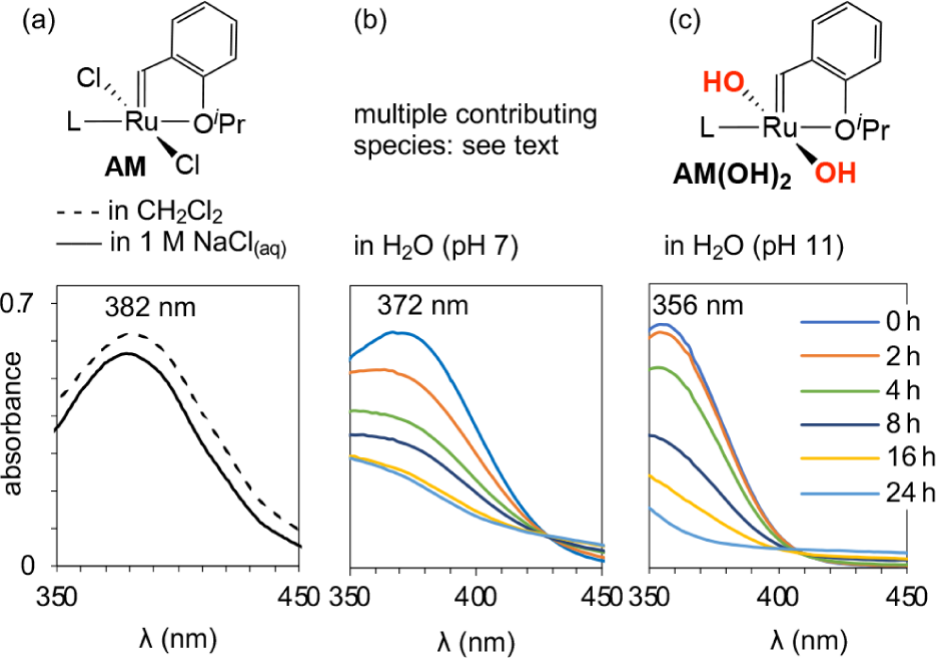
UV–vis spectra of Ru complexes over 24 h (0.1 mM Ru). (a) **AM** in CH_2_Cl_2_ or 1 M NaCl_(aq)_. For full spectra, see Figure S21. (b) Species formed from **AM** in water at pH 7 (reproduced from ref 11a). (c) Species formed from **AM(OH)**_**2**_ in water at pH 11.

The solubility of **AM** in polar organic solvents as well as water aids in establishing a clear-cut value for its absorption maximum. The principal absorption band observed in both anhydrous CD_2_Cl_2_ and ≥1 M NaCl_(aq)_ is centered at 382 nm (Figure 2a). It can therefore be assigned to the chelated dichlororuthenium structure depicted for **AM** in Figure 2, although confirmation by NMR analysis is precluded by the low solubility of **AM** in water at high chloride concentrations (0.14 mM in 1 M NaCl_(aq)_). In the absence of chloride, a solution-averaged band appears at 372 nm (Figure 2b), intermediate between the values for intact **AM** and the dihydroxide complex **AM(OH)**_**2**_ (Figure 2c).

### Establishing NMR and Spectrophotometric Signatures for AM(OH)_2_

In prior work, we synthesized **HII(OH)**_**2**_, the dihydroxide derivative of **HII** (Scheme 1), and demonstrated that it is inactive in metathesis.^18^ O’Reilly, Foster, and coworkers have suggested that such hydroxide species could account for the low activity of ruthenium metathesis catalysts in water.^12a^**AM(OH)**_**2**_ may thus represent a culminating structure in the decomposition cascade. We undertook its synthesis to bracket the anticipated structural extremes.

Addition of aqueous 2 M NaOH to **AM** caused an immediate color change from green to red. Pink **AM(OH)**_**2**_ was isolated in 93% yield by lyophilizing the water after 5 min, taking the residue up in THF, filtering off the Na salts, concentrating, and adding cold hexanes. The expected signal for the molecular ion dominates the electrospray mass spectrum in H_2_O (Figure S3), and the NMR signals in D_2_O are well resolved, enabling full assignment. Of particular note, the alkylidene singlet at 15.33 ppm is sharp (ω_0.5_ 2.1 Hz, vs 88.5 Hz for **AM**; compare Figures 2 and S2), and exhibits no correlation to the isopropyl protons by ^1^H–^1^H NOESY analysis, suggesting that the styrenyl ether remains chelated.^16b^ The principal UV–vis absorption band appears at 356 nm (Figure 2c) in water, at both neutral and basic pH.

**Scheme 3:**
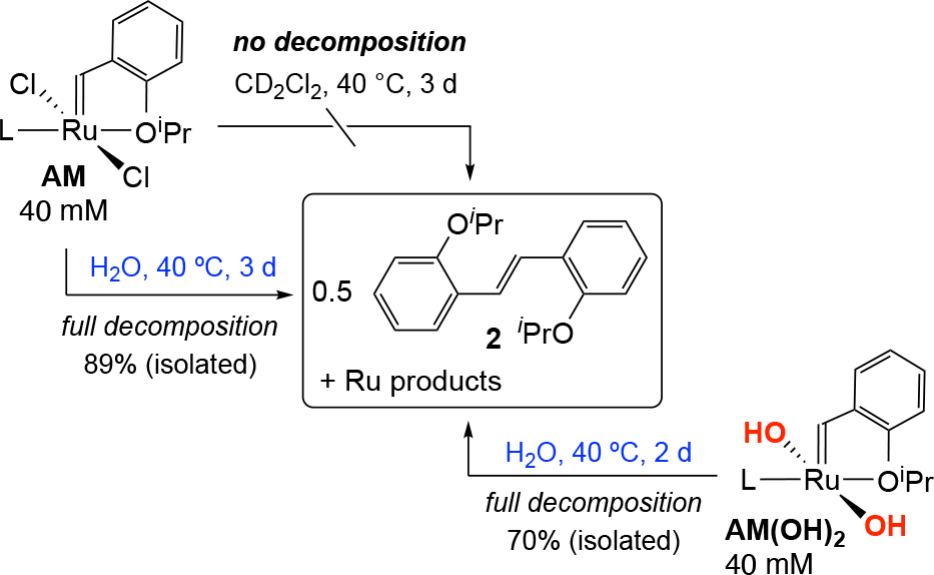
Evidence for Bimolecular Coupling of Benzylidene Species AM and AM(OH)_2_ in H_2_O

In anhydrous organic solvent, **AM** is stable for prolonged periods. No degradation is observed after 3 days at 40 °C in CD_2_Cl_2_, for example (Scheme 3). In contrast, it decomposes completely in water over the same period, with loss of the benzylidene ligand as the *trans*-stilbene **2**. The dihydroxide complex **AM(OH)**_**2**_ degrades similarly, at a faster rate.^19^ The observation of stilbene **2** for **AM** provides unequivocal evidence that bulk water promotes bimolecular coupling of such benzylidene species.^14^ Hydrogen-bonding between bridging water and/or hydroxide ligands is known to promote formation of OH_n_-bridged ruthenium dimers.^20,21,22^ In the present case, H-bonding plausibly aids coupling, resulting in elimination of **2** and formation of metathesis-inactive Ru(II) products.^23^ The high proportion of stilbene **2** formed under the conditions of Scheme 3 does not imply that benzylidene coupling will dominate at the lower catalyst concentrations normally employed. Nevertheless, it is striking that even Hoveyda-class precatalysts, although stable for prolonged periods in organic solvents, decompose via this pathway in water. We infer that bimolecular coupling of methylidene species—already very rapid in organic solvents^15a,15b,24^—is likely to play a major role in bulk water.

### Transformations of AM in Water

Having established the spectroscopic signatures for **AM** and **AM(OH)**_**2**_ in water, we undertook spectrophotometric study of the reactions of **AM** with H_2_O. Given the metathesis-inactivity of Ru–OH derivatives noted above, we particularly wished to identify the conditions under which the weakly acidic aqua species equilibrate into aqua-hydroxide mixtures. The absorption maximum seen immediately on dissolving **AM** in water in the *absence* of chloride appears at 372 nm, as noted above (Figure 2b). That is, it is blue-shifted by 10 nm relative to the 382 nm location established for **AM** itself. This signal represents an averaged band with multiple contributors, among which aqua derivatives are highly probable, from the classic chemistry of ruthenium in water.^25^

### Attempted Isolation of Aqua Species

**Scheme 4:**
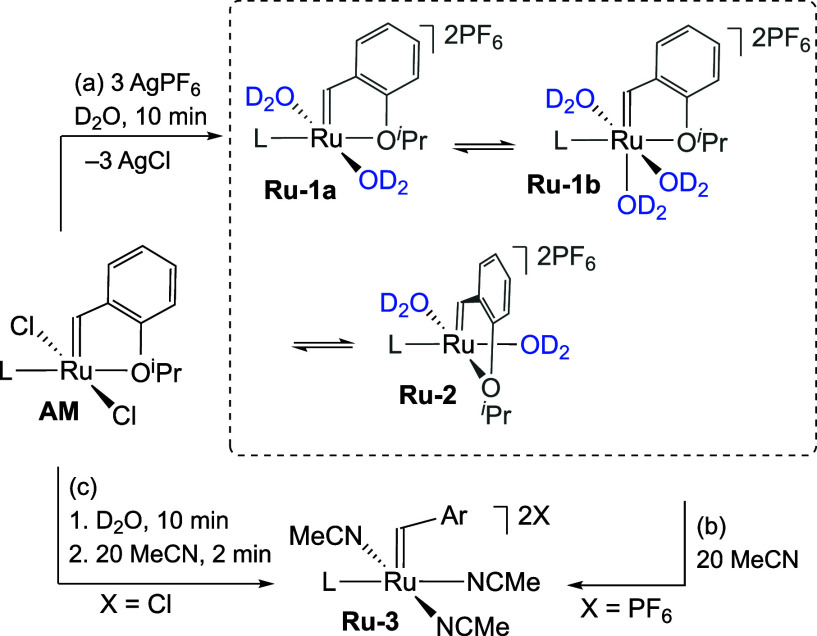
(a) Attempted Isolation of Aqua Complexes. (b) Convergence on Ru-3. (c) Independent Synthesis of Ru-3

**Figure 3:**
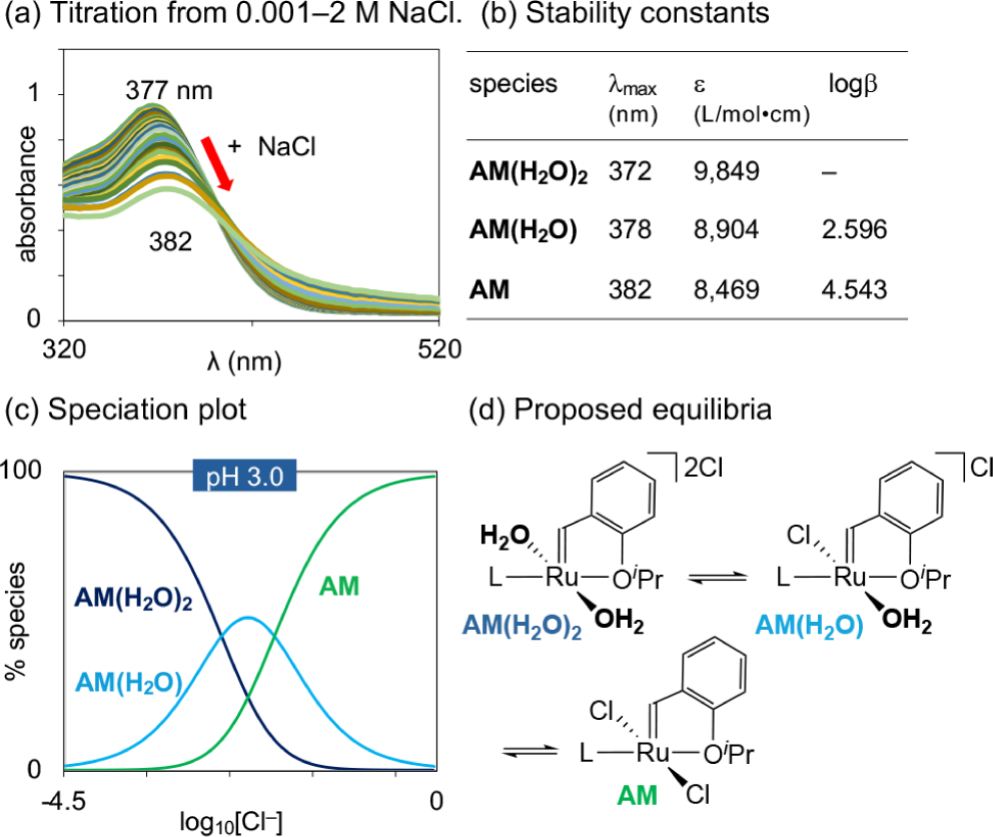
Speciation of **AM** at pH 3 as a function of chloride concentrations. (a) Chloride titration showing evolution of the electronic absorption spectra. (b) Stability constants β. (c) Speciation diagram. (d) Proposed equilibria. *Note*: additional aqua ligands may be present.

With the goal of intercepting the putative aqua species, we sought to abstract the chloride ligands from **AM** in water. Silver salts are the reagents of choice for this purpose,^26^ as precipitation of AgCl drives the reaction to completion. Accordingly, 3 equiv of AgPF_6_ was added to **AM** in D_2_O (Scheme 4a). The mixture was stirred for 10 min, then filtered through Celite to remove AgCl. Consistent with the lability of the aqua ligands, lyophilizing prior to redissolving in CD_2_Cl_2_ or CD_3_OD decomposes the sample. Direct spectrophotometric analysis of the green filtrate without lyophilizing reveals an absorption band at 370 nm, corresponding closely to that seen immediately on dissolving **AM** in water (cf. Figures 3b and S8d). The sharp ^1^H NMR signals in D_2_O, however, indicate success in arresting water-chloride exchange. Unexpectedly, three alkylidene singlets were observed in a 1:1:1 ratio (Figure S8a). A mixture of trans-**Ru-1a**, its six-coordinate water adduct **Ru-1b**, and cis-**Ru-2** appears most plausible (Scheme 4a), based on detailed NMR experiments, including EXSY evidence that the three complexes are in chemical exchange (Figure S8c).^27^ The presence of a hydroxide ligand is ruled out by the convergence of all three alkylidene species on dicationic **Ru-3** when MeCN is added (Scheme 4b,c). Their identification as aqua complexes rather than Ru-hydroxides is notable given the formal dicationic charge on Ru.

### Chloride Titration

Assigned above were the principal absorption bands for **AM** and **AM(OH)_2_**, at 382 and 356 nm, respectively. We now turn to the species that contribute to the averaged absorption band at 372 nm at pH 7, and the equilibria relating them. In initial experiments, we focused on equilibria involving chloride ion, to eliminate interference from background reactions involving (e.g.) dechelation of the ether donor, or formation of species varying only in the number of bound water ligands. We first assessed speciation as a function of chloride concentration at pH 3, where the negligible concentration^28^ of hydroxide (10^–11^ M) is anticipated to result in solely H_2_O–Cl equilibria. Accordingly, the chloride concentration was swept from 0.001 to 2.0 M by adding 5.0 M NaCl_(aq)_ at pH 3. After each addition, the system was allowed to equilibrate, and an aliquot was removed for UV–vis analysis. Representative spectra are shown in Figure 3a. The chloride concentrations and UV–vis data were analyzed using HypSpec^29^ to extract the stability constants (β) relating the species present. These data were refined by formulating a model of the system that takes into account stoichiometry, the number of species present, and the stability constants. The model was iteratively refined against the experimental data by modulating estimates of β and the number of species until an optimal fit was reached. A speciation plot was then generated via the HySS software, using the optimized equilibrium constants.

The stability constants and distribution curves obtained with the optimized model are shown in Figure 3b,c. The best-fitting model that emerges on data deconvolution^29^ requires two p*K*_Cl_ values: that is, two distinct equilibria involving replacement of water by chloride (Figure 3d). At pH 3 and the minimum chloride concentration of 0.001 M, the model is consistent with an equilibrium that favors diaqua complex **AM(H**_**2**_**O)**_**2**_. Sequential coordination of chloride generates **AM(H**_**2**_**O)**, then **AM**, as the NaCl concentration is increased. It should be noted that the accuracy of the log β values (and hence p*K*_Cl_) is limited by the changes in ionic strength inherent in these experiments.^30^

### pH Titration

**Figure 4:**
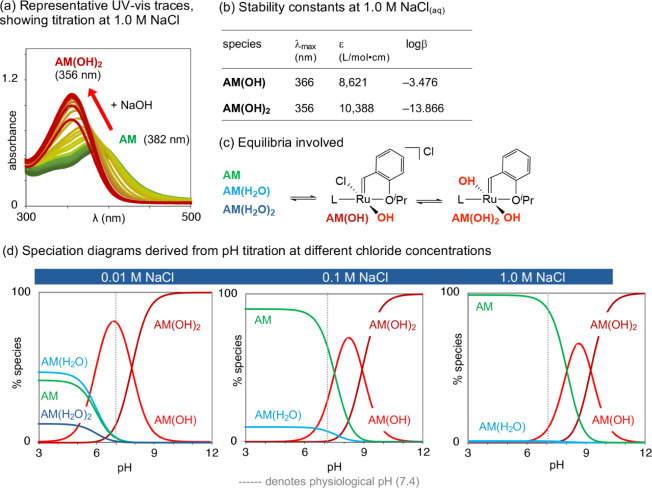
Speciation of **AM** in water as a function of pH at 0.01, 0.1, and 1.0 M NaCl_(aq)_. (a) Exemplary spectra showing spectrophotometric changes at 1.0 M NaCl_(aq)_. (b) Stability constants at 1.0 M NaCl_(aq)_ (for others, see Table S5). (c) Proposed equilibria. (d) Speciation diagrams.

To probe equilibria involving the deprotonation of bound water, we next mapped the impact of pH on speciation at fixed chloride concentrations. Accordingly, titration with 0.1 M KOH was undertaken, commencing at pH 3 (where the dichloride complex **AM** is present in significant proportions) and ending at pH 12, where the dihydroxide complex **AM(OH)**_**2**_ is the sole species present (Figure 2c). Within each set of experiments, the chloride concentration was maintained at a fixed value (0.01, 0.1, or 1.0 M NaCl_(aq)_). Figure 4a depicts representative spectrophotometric changes. Because the ionic strength is unaffected by changes in pH, it is constant within each set of titrations. The stability constants (Figure 4b) are hence of higher accuracy than those in the chloride titrations.^4^ Data deconvolution points toward two equilibria involving deprotonation of bound water: first, formation of mixed chloride-hydroxide species **AM(OH)**, and subsequently formation of dihydroxide **AM(OH)**_**2**_ (Figure 4c).

Figure 4d indicates that mono- and diaqua derivatives of **AM** are present even at pH 3, if chloride concentrations are low. At pH 7 and 0.01 M NaCl—conditions that approximate the pH-neutral, salt-free protocols routinely employed with **AM** in organic synthesis—the majority species present is the monohydroxide complex **AM(OH)**. The dominance of metathesis-inactive hydroxide species under these conditions helps account for the low productivity of **AM** under standard synthetic conditions. The deactivating effect of the hydroxide ligand is proposed to be due to slow initiation (a function of the inductive effect of the electronegative oxygen atom),^31^ exacerbated by rapid decomposition.^11^

### Implications for Catalysis

**Figure 5:**
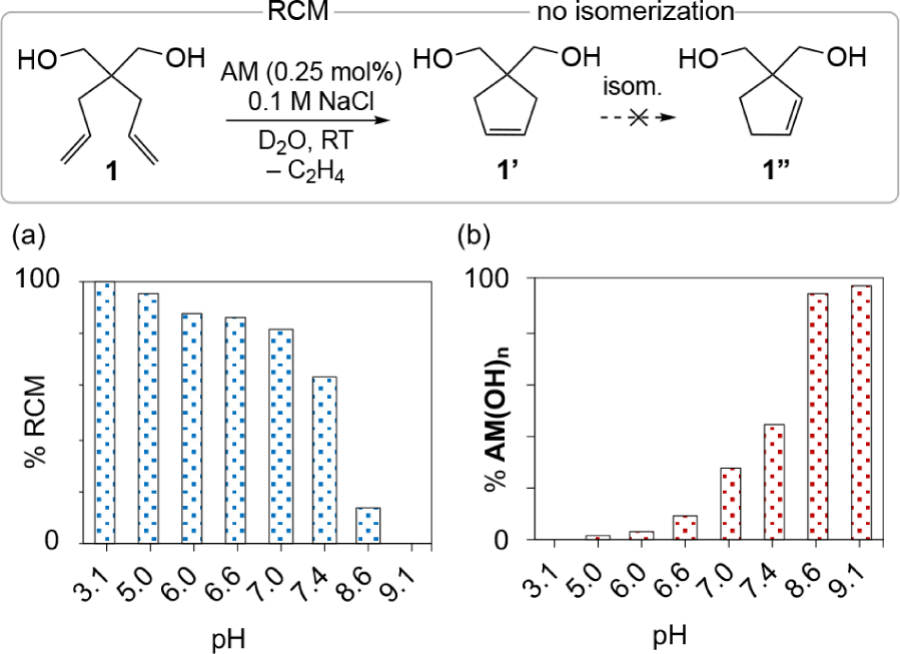
(a) Impact of pH on RCM productivity at 0.1 M NaCl_(aq)_. (b) Proportion of hydroxide species present (from Figure 4d, central plot).

To assess the correlation between aqueous speciation and metathesis productivity, we conducted RCM of diol substrate **1** in water at a series of different pH values, at a fixed NaCl_(aq)_ concentration of 0.1 M (Figure 5 and Table S7). This intermediate chloride concentration enables us to probe catalyst performance under conditions in which any of the dichloride, monohydroxide, or dihydroxide species may dominate, depending on pH. Catalyst loadings of 0.25 mol % **AM** were chosen, 20-fold lower than those routinely employed in aqueous RCM, to assess whether high RCM yields can be achieved at proportions of Ru better suited to organic synthesis, to biomolecule stability, or to minimizing toxicity^32^ for metathesis in living cells.^7a−7d^ Reactions were performed under inert atmosphere to isolate the impact of water from that of oxygen. (Experiments in air show minor decreases in productivity, with yields at 24 h declining by <10%: see Table S8.)

Shown in Figure 5a are RCM yields at 24 h over the pH range 3.1–9.1. The proportion of hydroxide species at each pH is given in Figure 5b. RCM is quantitative at pH 3.1, where **AM** is dominant and hydroxide formation is inhibited. Such strongly acidic conditions are less viable in chemical biology, however, owing to the susceptibility of many biomolecules (including proteins and native DNA or RNA) to degradation below pH 5.^33,34^ At pH 9.1, RCM fails completely, consistent with the speciation profile showing solely **AM(OH)**_**n**_ in this basicity regime. At physiological pH (7.4), metathesis proceeds with RCM yields only ca. 35% lower than those at pH 3, despite the use of 10× less catalyst than that required when no chloride is present (see Scheme 2). In principle, metathesis efficiency can be further improved by raising chloride concentrations to 1.0 M (Table S7). Limitations to this strategy for **AM** are imposed by reduced catalyst solubility, as noted above.^35^ Moreover, while high salt concentrations are often well-tolerated in synthetic chemistry, they represent a risk factor for precipitation or aggregation of biomolecules such as DNA and proteins.^36^ In chemical biology, millimolar chloride concentrations can thus represent a balance between the requirements of conserving catalyst, and maintaining substrate and catalyst solubility.

Use of NaI in place of NaCl causes TONs to drop ca. 7-fold (Table S8). The negative impact of the bulky iodide ligand on initiation rates is one contributor. However, we have shown that Ru-iodide catalysts can enable high metathesis productivity in ‘wet’ organic solvents,^13d,14^ in which they are fully soluble. Likewise, Tanaka’s Hoveyda-class bis-iodide ‘metathase’ (see Scheme 1) performs well within a hydrophobic pocket.^7e^ In bulk water, catalyst performance is undermined by the reduced solubility associated with the large, soft iodide ligand. The water-solubility of our best-in-class CAAC-sulfonate catalyst, for example, is 17 mg/mL, vs 5 mg/mL for its di-iodide analogue.^11a^

Where physiological pH is not mandated by biological constraints, speciation plots offer a valuable guide to the pH range that can be tolerated at different chloride concentrations. This opens the door to (e.g.) on-DNA metathesis in water. DNA-encoded libraries, as one topical example, could conceivably be achieved via RCM at pH 5–6 in 1 M NaCl_(aq)_, conditions that balance the proportion of the dichloride species against the limited tolerance of DNA for acidic conditions. Importantly, however, the viability of this approach for **AM** is limited by its poor solubility at high chloride concentrations.^35^ For challenging reactions in chemical biology, these data underscore the importance of catalyst solubility as a parameter for success.

Also of note in Figure 5 is the negligible isomerization observed at all pH values. This unexpected finding raises the possibility that chloride ion may inhibit isomerization by Ru decomposition products. It is particularly important given the aggressive isomerization in water revealed in Scheme 2.

## Conclusions

The foregoing provides the first detailed insight into the speciation behavior that controls the productivity of AquaMet (**AM**), the dominant catalyst in current use for aqueous olefin metathesis. On dissolution in water in the absence of added chloride, **AM** is immediately converted into a mixture of aqua species, which undergo further transformation into metathesis-inactive hydroxide complexes. High proportions of hydroxide species are present at biologically relevant pH, unless molar concentrations of chloride are present. However, high chloride concentrations severely restrict the solubility of **AM**. These findings reveal the basis for the lower productivity established for **AM**—and, more generally, for cationic metathesis catalysts—in water, relative to organic solvents. We conclude that metathesis catalysts designed for solubility in water at high chloride concentrations will be especially well suited to applications under physiological conditions and in aqueous media.

Metathesis in chemical biology is, famously, a race against decomposition. Speciation studies such as those presented herein provide an essential guide to the conditions for success. We anticipate that such studies may also offer crucial insights into catalyst interactions with complex biomolecules, a key step toward realizing the opportunities of olefin metathesis as a bioorthogonal strategy in chemical biology.

## Supporting Information Available

Experimental details and NMR, UV–vis, IR, and mass spectra (PDF)
