# Supplementary material for: Olefin Metathesis in Water: Speciation of a Leading Water-Soluble Catalyst Pinpoints Challenges and Opportunities for Chemical Biology
Source: J Am Chem Soc. 2025 Mar 7;147(11):9441–8. doi: 10.1021/jacs.4c16700 (PMC11926881; doi:10.1021/jacs.4c16700)
Supplement: Supplementary file 1 — ja4c16700_si_001.pdf [file ja4c16700_si_001.pdf]

*Supporting Information*

# Olefin Metathesis in Water: Speciation of a Leading Water-Soluble Catalyst Pinpoints Challenges and Opportunities for Chemical Biology

Christian O. Blanco,[a‡] Samantha K. Cormier,[a‡] Angus J. Koller,[b]
Eszter Boros,*[c] Deryn E. Fogg*[a,d]

[a]Center for Catalysis Research & Innovation, and Department of Chemistry and Biomolecular Sciences, University of Ottawa, Ottawa, Ontario, K1N 6N5, Canada. [b]Department of Chemistry, University of Southern Maine, Portland, Maine 04103, United States. [c]Department of Chemistry, University of Wisconsin–Madison, Madison, Wisconsin 53706, United States. [d]Department of Chemistry, University of Bergen, N-5007 Bergen, Norway.

*Corresponding authors: dfogg@uottawa.ca, dfo025@uib.no, eboros@wisc.edu

**Table of Contents**

**S1. Experimental.......................................................... S2**

S1.1. General Procedures.......................................................... S2

S1.2. Synthesis of Compounds.......................................................... S3

S1.3. Speciation Studies.......................................................... S6

S1.4. Catalytic Reactions in Water.......................................................... S8

S1.5. Monitoring Catalyst Stability by UV-Vis and NMR Spectroscopy.......................................................... S10

**S2. NMR Spectra and UV-Vis spectra.......................................................... S12**

**S3. References.......................................................... S45**

**S1. Experimental.**

**S1.1. General Procedures.** All reactions, other than a final set of catalytic experiments conducted in air, were carried out in an $N_2$-filled glovebox. Experiments with water were conveniently carried out in a dedicated glovebox using degassed water. NMR solvents ($CD_3OD$ and $D_2O$, Cambridge Isotopes, 99.5%; $CD_3CN$, ACP, 99.9%; $CD_2Cl_2$, ACP, 99.9%) and MilliQ $H_2O$ were freeze-pump-thaw degassed 4×, then stored under $N_2$ in the glovebox. Dimethyl sulfone ($Me_2SO_2$; Fisher Scientific, 99%, internal standard for NMR analysis), anthracene (Millipore, 96%, external standard for NMR analysis), NaCl (Sigma, 99.5%), $AgBF_4$ (Sigma, 98%), $AgPF_6$ (Strem, 99%), potassium trispyrazolyl borate (KTp; Sigma, 98%; used to quench metathesis reactions),[1] and **AM** (kindly provided as a gift by Apeiron Synthesis) were used as received. **AM** was assessed for purity ($^1$H NMR) prior to use, and weighed outside the glovebox on a microanalytical balance for accuracy. 2,2-Diallylpropane-1,3-diol **1** was prepared via the literature method.[2]

NMR spectra were recorded on Bruker Avance NMR spectrometers at 25.0 ±0.5 °C (Avance/ Avance II 300/400, Avance III 500/600). Chemical shifts (ppm) are referenced to the residual protons of the deuterated solvent. Quantitative NMR experiments used to quantify catalysis employed a standard delay time (D1) of 30 seconds. Electrospray (ESI) mass spectra were acquired with a Micromass Q-TOF I Mass Spectrometer (Waters) on 30 µg $mL^{-1}$ solutions in MeCN, MeOH or $H_2O$, injecting 1 mL volumes at 50 µL $min^{-1}$. Nebulization: $N_2$ (70 psi) at 200 °C; capillary and cone voltages 3.5 and 40 kV, respectively; source temperature 100 °C. IR spectra were collected on an Agilent Cary 630 Fourier Transform Infrared (FT-IR) spectrometer; ATR probe.

Spectrometric titrations were monitored using a NanoDrop Onec UV-vis spectrophotometer, conducted as out-of-cell titrations in the pH 2.0–13.0 regime. Figure S1 shows the setup for titration experiments, in which a Metrohm 6.0234.100 glass pH electrode was used with 3.0 M KCl reference pH, calibrated using a set of 3 standardized buffer solutions (pH 4.00, 7.00, 11.00). The HypSpec2014 program was used to analyze UV-vis titration data (minimum 20 individual spectra).[3,4] Aqueous solutions were lyophilized in vials by freezing in $N_{2(l)}$ and connecting to a vacuum line.

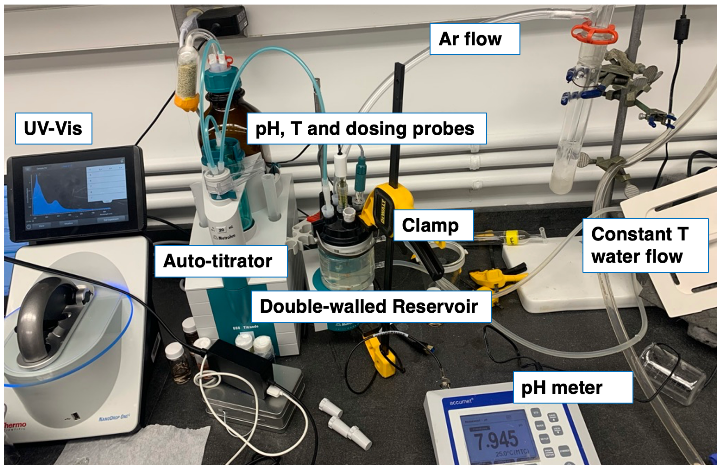


**Figure S1.** Experimental setup for titration experiments.

## S1.2. Synthesis of Compounds.

**Synthesis of AM(OH)₂.** Solid NaOH (80 mg, 2.0 mmol, 32 equiv) was added to a green solution of **AM** (50 mg, 0.062 mmol) in 2 mL $H_2O$. A colour change to red occurred immediately. The mixture was stirred for 5 min, then lyophilized. The residue was taken up in THF (2.5 mL) and immediately filtered through Celite to remove Na salts. The filtrate was concentrated to a minimum volume, and the product was precipitated with cold hexanes. The precipitate was filtered off, washed with hexanes (3 × 1 mL), and dried under vacuum. Yield of pink **AM(OH)₂**: 43 mg, 0.057 mmol (93%).

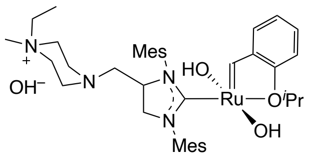
**AM(OH)₂**

$^1$H NMR (300 MHz, $D_2O$): δ 15.33 (s, 1H, [Ru]=*CH*), 7.44 (dd, $^3J_{HH}$ = 7 Hz, $^4J_{HH}$ = 2 Hz, 1H, *m*-C*H*), 7.24 (m, 4H, Mes C*H* + Ph *o*-C*H, p*-C*H*), 6.93 (m, 3H, Mes C*H* + Ph *m*-C*H*), 4.87 (sept, $^3J_{HH}$ = 6 Hz, 1H, C*H*Me₂), 4.79 (CH₂C*H* backbone; detected by $^1$H-$^{13}$C HSQC), 4.34 (m, $^3J_{HH}$ = 11 Hz, 1H, C*H*H backbone; diastereotopic), 3.88 (t, $^3J_{HH}$ = 10 Hz, 1H, C*H*H backbone; diastereotopic), 3.34 (m, 2H, N-C*H₂*Me), 3.18 (m, 3H, piperazine C*H₂*+ N-C*H*HCH), 2.95 (m, 6H, piperazine C*H₂* + N-C*H₃* + N-C*H*HCH), 2.73 (m, 4H, piperazine CH₂), 2.40 (s, 6H, C*H₃*), 2.36 (s, 3H, C*H₃*), 2.35 (s, 3H, C*H₃*), 2.28 (s, 3H, C*H₃*), 1.29 (t, $^3J_{HH}$ = 7 Hz, 3H, CH₂C*H₃*), 1.14 (d, $^3J_{HH}$ = 6 Hz, 6H, $^i$Pr C*H₃*). For fully-assigned spectrum (and 2D NMR spectra), see Figure S2.

$^{13}$C{$^1$H} NMR (76 MHz, $D_2O$): δ 269.5 ([Ru]=*C*H), 213.7 (NHC *C*:), 151.2, 143.6, 139.8, 139.5, 139.0, 137.6, 137.3, 135.8, 135.5, 129.7, 129.5(4), 129.4(5), 129.4, 127.5, 123.0, 120.8, 113.0, 74.6, 60.3, 59.6, 59.1, 57.0, 46.0, 45.6, 20.2, 20.0(8), 20.0(2), 19.9(9), 19.0, 17.9, 17.6, 17.4, 6.6.

ESI-MS ($H_2O$): Calculated for $C_{39}H_{57}N_4O_3Ru^+$ ([M]$^+$), *m/z* 731.3485. Found: *m/z* 731.3464 (Figure S3). UV-vis ($H_2O$, 25 °C): $\lambda_{max}$ 356 nm (ε = 10 388 L•mol$^{-1}$ cm$^{-1}$; Figure S4). IR (ATR): ν (O-H) 3355 cm$^{-1}$ (w); Figure S5).

**Synthesis of [AM(MeCN)₃](PF₆)₃, Ru-3**. Addition of $AgPF_6$ (15 mg, 0.058 mmol, 3.1 equiv) to a stirred green solution of **AM** (15 mg, 0.017 mmol) in MeCN (2 mL) caused an immediate colour change to orange-yellow. Reaction was continued at RT for 20 min, after which the suspension was filtered through Celite to remove AgCl. The solvent was removed under vacuum and the resulting orange-red solid was dried under vacuum. Yield: 21 mg, 0.015 mmol (88%).

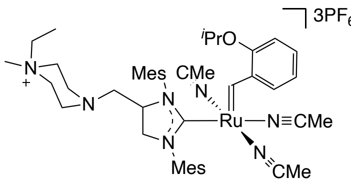
**[AM(MeCN)₃]**PF₆

$^1$H NMR (600 MHz, $CD_3CN$): δ 16.94 (s, 1H, [Ru]=*CH*), 8.08 (dd, $^3J_{HH}$ = 8 Hz, $^4J_{HH}$ = 2 Hz, 1H, Ph *o*-C*H*), 7.76 (dd, $^3J_{HH}$ = 8 Hz, $^4J_{HH}$ = 2 Hz, 1H, Ph *p*-C*H*), 7.04 (t, $^3J_{HH}$ = 8 Hz, 1H, Ph *m*-C*H*), 6.95 (d, $^3J_{HH}$ = 9 Hz, 1H, Ph *m*-C*H*), 6.87 (s, 1H, Mes C*H*), 6.84 (s, 2H, Mes C*H*), 6.81 (s, 1H, Mes C*H*), 4.63 (sept, $^3J_{HH}$ = 6 Hz, 1H, C*H*Me₂), 4.37 (m, 1H, CH₂C*H* backbone), 4.06 (m, 1H, C*H*H backbone; diastereotopic), 3.65 (m, 1H, C*H*H backbone; diastereotopic), 3.26 (q, 2H, $^3J_{HH}$ = 7 Hz, N-C*H₂*Me), 3.10 (m, 2H, piperazine C*H₂*), 3.05 (m, 2H, piperazine C*H₂*), 2.84 (s, 3H, N-C*H₃*), 2.68–2.56 (m, 4H, piperazine C*H₂*), 2.56–2.46 (m, 2H + N-C*H₂*CH), 2.31 (s, 3H, C*H₃*), 2.29

(s, 3H, C$H_3$), 2.25 (s, 6H, C$H_3$), 2.15 (s, 3H, C$H_3$), 2.13 (s, 3H, C$H_3$), 1.40 (d, $^3J_{HH}$ = 6 Hz, 3H, $^i$Pr C$H_3$), 1.36 (d, $^3J_{HH}$ = 6 Hz, 3H, $^i$Pr C$H_3$), 1.23 (t, $^3J_{HH}$ = 7 Hz, 3H, $CH_2CH_3$). For fully-assigned $^1$H NMR spectrum, see Figure S6. The complex decomposes immediately in $CD_2Cl_2$ but is stable if >20 equiv MeCN is present (Figure S7).

$^{13}$C{$^1$H} NMR (151 MHz, $CD_3CN$): δ 321.3 ([Ru]=*C*H), 207.7 (NHC *C*:), 152.2, 144.2, 139.9, 139.7, 139.5, 139.2, 137.7, 137.6, 137.5, 137.2, 136.6, 135.6, 131.0, 130.7, 130.6, 130.3, 128.0, 127.4, 122.1, 115.4, 105.9, 72.9, 68.2, 61.8, 60.5, 59.4, 58.0, 46.9, 46.5, 26.2, 22.4, 22.2, 21.0, 19.8, 18.6, 18.3, 18.2.

ESI-MS (MeCN): Calculated for $C_{45}H_{64}N_7ORu^+$ ($[M]^+$), *m/z* 820.4199. Found: *m/z* 820.4316.

**Generation of Aqua Derivatives via Chloride Abstraction with $AgPF_6$ in Water.** In a darkened glovebox, a solution of $AgPF_6$ (28 mg, 0.11 mmol, 3 equiv) in 0.5 mL $D_2O$ was added dropwise to a stirred green solution of **AM** (30 mg, 0.037 mmol) in 2 mL $D_2O$. After 20 min at RT, solid AgCl was removed by filtration. The green filtrate was analyzed directly ($^1$H NMR; UV-vis; Figure S8). $^1$H NMR (300 MHz, $D_2O$): δ 18.66 (s), 18.38 (s), 18.32 (s) 1:1:1. UV-vis ($H_2O$, 25 °C): $\lambda_{max}$ 370 nm, ε = 9 849 $M^{-1}cm^{-1}$). IR (ATR): ν (O-H) 3503 $cm^{-1}$ (m); after lyophilization (Figure S9). For additional experiments, see below.

$^1$H-$^1$H NOESY (500 MHz, $D_2O$, 5 °C to minimize decomposition). Relaxation delay (D1) 2 s, mixing time 0.3 s; phase of diagonal peaks set as positive. NOE correlations are negative, EXSY correlations positive. Positive correlations between the three alkylidene species confirm chemical exchange. For spectrum, see Figure S8c.

2D DOSY (300 MHz, $D_2O$, 5 °C; Figure S10). Diffusion delay (Δ) 220 ms, gradient pulse duration (δ) 3 ms; 80 scans per increment; relaxation delay (D1) 1 s. Diffusion coefficients are summarized in Table S1.

**With $AgBF_4$:** Use of $AgBF_4$ (21 mg, 0.11 mmol, 3 equiv, or excess; 5 equiv) gave a product distribution (Figure S11) identical to that observed with 3 equiv $AgPF_6$, confirming that (i) the products are independent of the silver salt, and (ii) that no chloride ligands remain (that is, ruling out potential stoichiometric deficiencies from degradation of the photosensitive Ag reagent).

**Table S1.** Diffusion Coefficients for Aqua Derivatives in $D_2O$.

| **Species** | **δ** | **Log D** | **D ($m^2/s$)** |
|---|---|---|---|
| **Ru-1a** | 18.66 | –9.817 | $1.524\times10^{-10}$ |
| **Ru-1b** | 18.38 | –9.780 | $1.660\times10^{-10}$ |
| **Ru-2** | 18.32 | –9.780 | $1.66\times10^{-10}$ |
| HOD | 4.79 | –8.956 | $1.107\times10^{-9}$ |

**Addition of MeCN:** Dilution of the green $D_2O$ solution from the $AgPF_6$ reaction with MeCN (40 µL, 0.74 mmol, 20 equiv) caused an immediate colour change to yellow-orange and collapse of the three alkylidene species into the singlet for [**AM(MeCN)$_3$**]$(PF_6)_3$ (17.02 ppm). For spectra, see Figures S12, S15. This experiment rules out the presence of a hydroxide ligand in the three complexes present in $D_2O$.

**Lyophilization and Redissolution.** Alternatively, the green $D_2O$ solution was lyophilized and redissolved. In $CD_2Cl_2$: colour change to yellow-green; partial regeneration of **AM** via chlorination by solvent, accompanied by decomposition. $^1$H NMR (300 MHz, $CD_2Cl_2$): 16.41 ppm; loss of intensity of all signals (Figure S13a; cf. Figure 1). In $CD_3OD$: Immediate colour change to brown. $^1$H NMR (300 MHz, $CD_3OD$): no Ru=C*H*R signals (Figure S13b). In $CD_3CN$: complete conversion to [**AM(MeCN)$_3$**]$PF_6$. $^1$H NMR (600 MHz, $CD_3CN$): δ 16.93 (Figure S14).

**Reaction of AM with MeCN in Water**. Green **AM** (10 mg, 0.012 mmol) was dissolved in $D_2O$ (0.75 mL) and transferred to a screw-capped NMR tube with a septum seal. The $^1$H NMR spectrum (Figure S16) exhibits significant signal broadening. Injection of MeCN (12 µL, 0.24 mmol, 20 equiv; puncture promptly covered with Parafilm) via gas-tight syringe caused an immediate colour change to yellow-orange. Formation of [**AM(MeCN)$_3$**]$Cl_3$ was confirmed by NMR analysis vs fully-characterized [**AM(MeCN)$_3$**]$(PF_6)_3$ above. $^1$H NMR (300 MHz, $D_2O$): δ 17.00 (Figure S17).

**Synthesis of Phosphonium Chloride 3.** To a Schlenk flask containing *o*-isopropoxybenzyl chloride (1.32 g, 7.15 mmol) in 8 mL toluene was added solid $PPh_3$ (2.06 g, 7.86 mmol, 1.1 equiv) against a flow of $N_2$. The reaction was stirred at 80 °C for 16 h. An additional (491 mg, 0.26 equiv) of $PPh_3$ was added. The reaction was stirred overnight, after which the white solid was filtered off, washed with hexanes, and dried under vacuum. Yield: 2.24 g, 5.01 mmol (70% yield). For full assignment, see Figure S18.

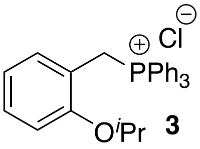

**3**

$^1$H NMR (600 MHz, $CDCl_3$): δ 7.77–7.74 (m, 3H, P*Ph$_3$*), 7.61–7.58 (m, 12H, P*Ph$_3$*), 7.25 (d, $^3J_{HH}$ = 7.2 Hz, 1H, Ar), 7.19 (t, $^3J_{HH}$ = 8 Hz, 1H, Ar), 6.64 (tt, $^3J_{HH}$ = 7.5 Hz, $^4J_{HH}$ = 1.0 Hz, 1H, Ar), 6.61 (d, $^3J_{HH}$ = 8 Hz, 1H, Ar), 5.31 (d, $^3J_{HP}$ = 14 Hz, 2H, Ar-C*H$_2$* ), 4.16 (d, $^3J_{HH}$ = 6 Hz, 1H, C*H* $^i$Pr), 0.95 (d, $^3J_{HH}$ = 6.0 Hz, 6H, C*H$_3$* $^i$Pr).

$^{13}$C{$^1$H} (151 MHz, $CDCl_3$): δ 21.8 (*C*H$_3$ $^i$Pr), δ 25.2 (d, $^3J_{PC}$ = 48 Hz, Ar-*C*H$_2$), δ 69.8 (*C*H $^i$Pr), δ 112.1 (d, $J_{PC}$ = 3 Hz, Ar), δ 116.2 (d, $J_{PC}$ = 9 Hz, Ar), δ 118.2 ($PPh_3$), δ 118.7 ($PPh_3$), δ 120.9 (d, $J_{PC}$ = 3 Hz, Ar), δ 130.1 (Ar), δ 130.2 (*d*, $J_{PC}$ = 13 Hz, $PPh_3$), δ 132.7 (d, $J_{PC}$ = 4 Hz, Ar), δ 134.4 (d, $J_{PC}$ = 9 Hz, $PPh_3$), δ 135.0 (d, $J_{PC}$ = 3 Hz, $PPh_3$), δ 155.9 (d, $J_{PC}$ = 5 Hz, Ar-O).

$^{31}$P{$^1$H} NMR (121 MHz, $CDCl_3$): δ 22.2.

ESI-MS (MeCN): Calc'd for $C_{28}H_{28}OP^+$ ($[M]^+$), *m/z* 411.1872. Found: *m/z* 411.1887.

**Synthesis of Trans-Stilbenoid 2.** Mixing white **3** (1.5 g, 3.36 mmol, 1.25 equiv), $^t$BuOK (380 mg, 3.39 mmol, 1.26 equiv), and THF (20 mL) in a Schlenk flask gave an orange solution. The septum-sealed flask was transferred to a Schlenk line and cooled to –63 °C ($CHCl_3$/$N_2$ bath). *o*-Isopropoxybenzaldehyde (441 mg, 2.69 mmol) was injected dropwise. The reaction was allowed to warm to RT and stirred overnight, after which no starting aldehyde was detected (TLC, silica). The solvent was removed under vacuum and the residue was purified by column chromatography in $CH_2Cl_2$ to yield a clear oil. 495 mg, 1.67 mmol (62%). For full assignment, see Figure S19.

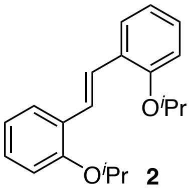

**2**

$^1$H NMR (300 MHz, $CDCl_3$): δ 7.16 (dd, $^3J_{HH}$ = 7.8, $^4J_{HH}$ = 1.7 Hz, 2H, Ar), 7.13 (td, $^3J_{HH}$ = 7.8, $^4J_{HH}$ =1.6 Hz, 2H, Ar), 6.87 (d, $^3J_{HH}$ = 8.0 Hz 2H, Ar), 6.74 (s, 2H, C*H*=C*H*), 6.69 (td, $^3J_{HH}$ = 7.5, $^4J_{HH}$ =1.0 Hz, 2H, Ar), 4.54 (sept, $^3J_{HH}$ = 6.1 Hz, 2H, C*H* $^i$Pr), 1.33 (d, $^3J_{HH}$ = 6.1 Hz, 12H, C$H_3$ $^i$Pr).

$^{13}$C{$^1$H} (151 MHz, $CDCl_3$): δ 22.4 (*C*$H_3$ $^i$Pr), δ 71.1 (*C*H $^i$Pr), δ 114.5 (Ar), δ 120.2 (Ar), δ 125.6 (*C*H=*C*H), δ 128.1 (Ar-CH=CH), δ 128.2 (Ar), δ 130.3 (Ar), δ 155.9 (Ar-O$^i$Pr).

ESI-MS ($CH_2Cl_2$): Calc'd for $C_{20}H_{24}O_2Na^+$ ($[M+Na]^+$), *m/z* 319.1674. Found: *m/z* 319.1663.

**S1.3. Speciation Studies.** The experiments below were conducted under Ar to facilitate air-exclusion. MilliQ water was degassed by sonicating under vacuum (3 × 5 min) and back-filling the flask with Ar. Solutions A–E were prepared by adding weighed amounts of 0.1 M HCl and NaCl or KCl (for details, see Table S2) to a volumetric flask and diluting to the line with degassed water added under a flow of Ar. The double-walled titration reservoir was sealed with a 4-inlet lid, probes for pH, temperature, and dosing were inserted into the inlets, and the fourth was stoppered, all under a positive pressure of Ar. To charge the vessel, the stopper was removed, the reservoir solution was added under a positive pressure of Ar, the inlet was sealed, and the system was purged with Ar (5 min). Solid **AM** was added via the inlet against a positive pressure of Ar. Titrant was added via the dosing probe and aliquots removed by pipette against a flow of Ar. Titrant and aliquot volumes were recorded over the course of the experiment to accurately track the concentrations of chloride and **AM** (i.e. to factor in dilution of the initial solutions by added titrant).[5] Table S3 summarizes the absorption maxima ($\lambda_{max}$) and molar absorptivities (ε; calculated using the known initial concentration of **AM**).

**Table S2.** Volumes and Masses of Solutes for Aqueous Solutions

| Solution | $H_2O$ (mL) | HCl (0.1 M; mL) | KCl (g) | pH | HCl (M) | KCl (M) | Ionic strength (M) |
|---|---|---|---|---|---|---|---|
| A | 50 | 5.00 | — | 2 | 0.01 | — | — |
| B | 250 | 2.50 | 73.06[a] | 3 | 0.001 | 5.0[a] | – |
| C | 50 | 5.01 | 0.336 | 2 | 0.01 | 0.09 | 0.09 |
| D | 100 | 9.93 | 7.38 | 2 | 0.01 | 0.99 | 0.99 |
| E | 100 | 1.00 | — | 2 | 0.01 | — | 0.01 |

[a]NaCl was employed for its higher water-solubility.

**Representative Procedure for Chloride Titration.** Solid **AM** (8.0 mg, 0.01 mmol; 100 μM) was added to a reservoir of Solution A (final concentration 100 μM). The chloride concentration was swept from 0.001–2.0 M at pH 3 by addition of 5.0 M $NaCl_{(aq)}$ (Solution B) to the stirred solution at pH 3. After adding each aliquot of NaCl, the solution was permitted to equilibrate until potential readings stabilized at a constant value prior to withdrawing an aliquot for analysis. A 2 mL aliquot was transferred to a cuvette, sealed, and analyzed (UV-vis). Titration was continued to 2 M Cl, at which point no further spectral changes were evident.

**Representative Procedure for pH Titration.** As above, using **AM** (4.0 mg, 0.0049 mmol) and 50 mL Solution C (final concentration 87 μM). The solution was titrated with 0.1 M KOH from pH 2–11.

**Table S3.** Summary of Spectrophotometric Parameters for **AM** and its Derivatives.

| Complex | $\lambda_{max}$ (nm) | ε ($M{\bullet}cm^{-1}$) |
|---|---|---|
| **AM** | 382 | 8,469 |
| **AM(OH)** | 366 | 8,621 |
| **$AM(OH)_2$** | 356 | 10,388 |
| **$AM(H_2O)$** | 378 | 8,904 |
| **$AM(H_2O)_2$** | 372 | 9,849 |

UV-vis titrations were analyzed using the HypSpec2014 program.[3,4] A limited number of species was initially considered, with incremental expansion, stopping once further additions had no significant effect on the fitting. Preliminary estimates of stability constants were derived from the raw data to establish an initial fit, after which nonlinear regression was applied to refine the

stability constants and molar absorptivities. The plausibility of the model was validated by ensuring the spectral fit matched observed changes, i.e., to avoid fitting noise to any species. The best fitting was obtained for a system of four equilibria, for which the cumulative equilibrium constants β are shown in Table S4. Speciation diagrams (Figures 4, 5) were plotted using the HySS program using the numerical values of logβ (Table S5).

**Table S4.** Equilibria Involved in the Speciation of **AM** in Water.

| Constant | Equilibrium | Expression |
|---|---|---|
| $\beta_1$ | $Cl^- + AM(H_2O)_2 \longrightarrow AM(H_2O)$ | $\frac{[AM(H_2O)]}{[Cl^-][AM(H_2O)_2]}$ |
| $\beta_2$ | $2Cl^- + AM(H_2O)_2 \longrightarrow AM$ | $\frac{[AM]}{[Cl^-]^2[AM(H_2O)_2]}$ |
| $\beta_3$ | $AM(H_2O)_2 \longrightarrow AM(OH) + H^+$ | $\frac{[AM(OH)][H^+]}{[AM(H_2O)_2]}$ |
| $\beta_4$ | $AM(H_2O)_2 \longrightarrow AM(OH)_2 + 2H^+$ | $\frac{[AM(OH)_2][H^+]^2}{[AM(H_2O)_2]}$ |

**Table S5.** Stability Constants (logβ) from Chloride and pH Titrations.

| [NaCl] (M) | **AM($H_2O$)** (log$\beta_1$) | **AM** (log$\beta_2$) | **AM(OH)** (log$\beta_3$) | **AM(OH)$_2$** (log$\beta_4$) |
|---|---|---|---|---|
| 0.01 | 2.60(3) | 4.54(3) | –3.04(1) | –12.88(1) |
| 0.1 | 2.60(3) | 4.54(3) | –3.96(1) | –13.87(3) |
| 1.0 | 2.60(3) | 4.54(3) | –3.48(1) | –12.70(2) |

## S1.4. Catalytic Reactions in Water.

**RCM of Diene 1 at a Range of pH Values.** Diene **1** (16 mg, 0.10 mmol), NaCl (6 mg, 0.1 mmol, 1 equiv), and dimethyl sulfone (9 mg, 0.1 mmol, 1 equiv; internal standard) were dissolved in 0.90 mL $H_2O$. The pH was adjusted to the desired level by adding HCl or NaOH; final concentration 100 mM **1**. A 50 μL aliquot was removed for NMR analysis to establish the starting ratio of **1**:$Me_2SO_2$. Catalyst was added to the stirred solution as a 20 μL aliquot of a stock solution of **AM** (10.1 mg **AM** in 1.0 mL $H_2O$): final catalyst loading 0.25 mol% (0.25 mM). The solution was stirred at RT for 24 h, quenched as above, lyophilized, redissolved in $D_2O$, and analyzed by integrating the olefinic NMR signals for **1'** and **1"** (Table S6, signals in $D_2O$ assigned by analogy to those reported[6] in $CDCl_3$). Yields and conversions are shown in Table S7.

**Table S6**. Diagnostic $^{1}$H NMR Signals for **1** and **1’**

| Compound | Proton | Role | $\delta_H$ $CDCl_3$ | $\delta_H$ $D_2O$ |
|---|---|---|---|---|
| **1’** | 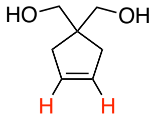 | Product | 5.65 | 5.63 |
| **1’’** | 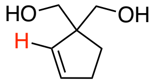 | Isomerized product | 5.96 | 5.91 |

**Table S7.** RCM of **1** by **AM** (0.25 mol%) in $D_2O$ at Different pH Values

| [NaCl] (M) | pH | Conversion (%) | Yield (%) | TON |
|---|---|---|---|---|
| 0.1 | 3.10 | 100 | 100 | 400 |
| 0.1 | 5.02 | 97 | 95 | 380 |
| 0.1 | 6.04 | 88 | 88 | 352 |
| 0.1 | 6.57 | 86 | 86 | 344 |
| 0.1 | 7.01 | 82 | 82 | 328 |
| 0.1 | 7.45 | 66 | 64 | 256 |
| 0.1 | 8.55 | 14 | 14 | 56 |
| 1.0 | 7.40 | 100 | 100 | 400 |

**Catalytic Performance of AM in Water: Representative RCM Procedure.** Diene **1** (16 mg, 0.10 mmol) and dimethyl sulfone, $Me_2SO_2$ (9 mg, 0.1 mmol, 1 equiv; internal standard) were dissolved in 0.90 mL $D_2O$; final concentration 100 mM **1**. A 50 µL aliquot was removed for NMR analysis to establish the initial ratio of **1**:$Me_2SO_2$. To the stirred solution was added 2.0 mol% **AM** (80 µL of a stock solution of 10.1 mg **AM** in 1.0 mL $D_2O$). Aliquots were removed periodically, quenched with KTp in $D_2O$ (10 mg/mL; 10 equiv vs starting Ru) and analyzed (NMR). A representative spectrum is given in Figure S20.

In air: These tests were carried out at a lower loading than Figure 5 in the main text, to permit comparison at intermediate conversions. Experiments were conducted as above with 0.05 mol% **AM** (40 µL of a diluted stock solution of 1.0 mg **AM** in 1 mL $D_2O$) and preparing all solution with $D_2O$ from the bench and stirring in an open vial in air.

With NaI 0.1 M: As above, with additinon of NaI (14 mg, 0.10 mM, 1 equiv), in the glovebox under $N_2$. Yields, conversions, and TONs are shown in Table S8.

**Table S8.** Impact of Air and Na Counter-Anion on RCM of **1** by **AM** (0.05 mol%) in $D_2O$.

| Salt | T (ºC) | Atmosphere | Conversion (%) | Yield (%) | TON |
|---|---|---|---|---|---|
| NaCl | RT | $N_2$ | 27 | 27 | 540 |
| NaCl | RT | Air | 21 | 20 | 400 |
| NaI | RT | $N_2$ | 4 | 4 | 80 |
| NaI | 60 | $N_2$ | 4 | 4 | 80 |

**S1.5. Monitoring Catalyst Stability by UV-Vis and NMR Spectroscopy.**

**Monitoring Catalyst Stability by UV-Vis Spectrophotometry.** To a quartz cuvette was added $H_2O$ (1.97 mL, pH 7 prior to catalyst addition), and a 30 µL aliquot of a stock solution of **AM** in water (5 mg/mL); final Ru concentration of 30 µM. The cuvette was sealed, wrapped with Parafilm and removed from the glovebox to the spectrometer. The first UV-vis spectrum was taken 5 min after preparing the catalyst stock solution. Subsequent spectra were recorded periodically up to 24 h. UV-vis spectra showing the stability of **AM** vs **AM(OH)₂** appear in Figure 2 in the main text. Reference spectra for the bis-chloride complex **AM** in 1.0 M aqueous NaCl, MeOH, and $CH_2Cl_2$ are shown in Figure S21.

**Monitoring Stability of AM in $CD_2Cl_2$.** A solution of green **AM** (10 mg, 0.012 mmol) and DMT (ca. 1.0 mg) in 0.75 mL $CD_2Cl_2$ was prepared in a J. Young NMR tube. The initial ratio of **AM**:IS was measured, after which the NMR sample was transferred to an oil bath behind a blast shield. The mixture was analyzed (NMR) every 24 h. No change was observed over 3 days (Figure S22a). Similarly, no decomposition was observed at higher concentrations (40 mM **AM**, Figure S22b).

**Analysis of Decomposition Products of AM in $D_2O$.** Green **AM** (10 mg, 0.012 mmol) was dissolved in $H_2O$ and stirred (glovebox, degassed oil bath) at 40 ±1 °C. The solution was analyzed every 24 h by UV-vis spectroscopy to assess the disappearance of any CT bands. After three days, no discrete bands were observed. The mixture was then lyophilized, and the resulting orange solid was extracted with $CDCl_3$ (3 × 1 mL) and filtered through Celite. To the filtrate was added anthracene (400 µL, 0.0449 mmol, from a stock solution 20 mg/mL in $CDCl_3$). The solution was stirred vigorously, then subjected to $^1$H NMR and GC-MS analysis (Figure S23).

**Determining the Solubility of AM in 1 M $NaCl_{(aq)}$.** To green **AM** (10 mg, 0.012 mmol) was added a solution of 1 M $NaCl_{(aq)}$ (1 mL). The resulting green suspension was stirred for 10 min in a sonicator and then centrifuged. An aliquot (10 µL) of the supernatant was diluted in 1 M $NaCl_{(aq)}$ (2 mL total volume) and transferred to a quartz cuvette for UV-vis analysis. The value of the molar absorbance at 382 nm (0.260) and the corresponding absorption coefficient (Table S3) were used to calculate the molar solubility of **AM**.

**Excluding the Possibility of Paramagnetic Species.** A solution of green **AM** (10 mg, 0.012 mmol) in 0.75 mL $D_2O$ was prepared in a screw-capped NMR tube equipped with a septum. Immediate $^1H$ NMR analysis revealed significant line-broadening (Figure 1, main text). To assess the contribution of dynamic or exchange broadening versus potential paramagnetic impurities, $CD_3OD$ (370 µL) was injected into the tube using a gas-tight syringe. The puncture was promptly covered with Parafilm and re-analyzed. All NMR signals sharpened (Figure S24; $\omega_{0.5}$ = 20 Hz for the alkylidene singlet; cf. 89 Hz in neat $D_2O$). These observations suggest that the initial broadening arises from dynamic processes ,rather than paramagnetic species.

**Variable Temperature NMR of AM.** A solution of green **AM** (10 mg, 0.012 mmol) in 0.75 mL $D_2O$ was prepared in a J. Young NMR tube. The sample was cooled to 5 ºC, permitted to equilibrate for 10 min, and analyzed immediately (NMR). The alkylidene resonance remained poorly resolved (Figure S25).

**Attempted Observation of Hydrides or Dimers.** A green solution of **AM** (10 mg, 0.012 mmol) in 0.75 mL $D_2O$ in a J. Young NMR tube was removed from the glovebox and heated in a thermostatted oil bath to 40 ±1 °C behind a blast shield. A colour change to brown-orange occurred over 2 days. $^1H$ NMR analysis revealed complete loss of the alkylidene signal. Multiple broad signals were present in the aliphatic region (Figure S26a). No signals were observed in the region typical for ruthenium hydride species (–5 to –30 ppm). Similarly, no deuterium-containing species formated via reaction with $D_2O$ were observed by $^2H$ NMR analysis (Figure S26b). ESI-MS analysis ($H_2O$) showed no species in the mass range for dimers (Figure S27).

**Determining the Impact of Dechelation on the Benzylidene o-C*H* Signal in AM.** A solution of **AM** (16 mg, 0.020 mmol) in 0.75 mL $CD_2Cl_2$ in a J. Young NMR tube was analyzed ($^1H$-$^{13}C$ HMBC; Figure S28a) to locate the aromatic C*H* proton *ortho* to the alkylidene carbon in the benzylidene ring, via its correlation with the alkylidene proton at 6.93 ppm (Figure S28b). The NMR tube was returned to the glovebox and solid $PCy_3$ (28 mg, 0.10 mmol, 5 equiv) was added and mixed by shaking. After 20 min, the solution had turned brown brown. NMR analysis revealed 16% **AM** and four new alkylidene signals between 19.6–19.9 ppm (Figure S29a), in excellent agreement with the value of 19.88 ppm in $CDCl_3$ reported for the corresponding dechelated **HII**-$PCy_3$ adduct.[7] The additional signals for the **AM-PCy₃** reflect the unsymmetrical nature of the NHC ligand. The alkylidene resonances exhibit the expected $^1H$-$^{13}C$ HMBC correlations to the *o*-C*H* multiplets of the benzylidene ring between 8.7–8.8 ppm (Figure S29b).

## S2. NMR Spectra and UV-Vis Spectra.

(a) $^{1}$H NMR spectrum of **AM(OH)$_2$**

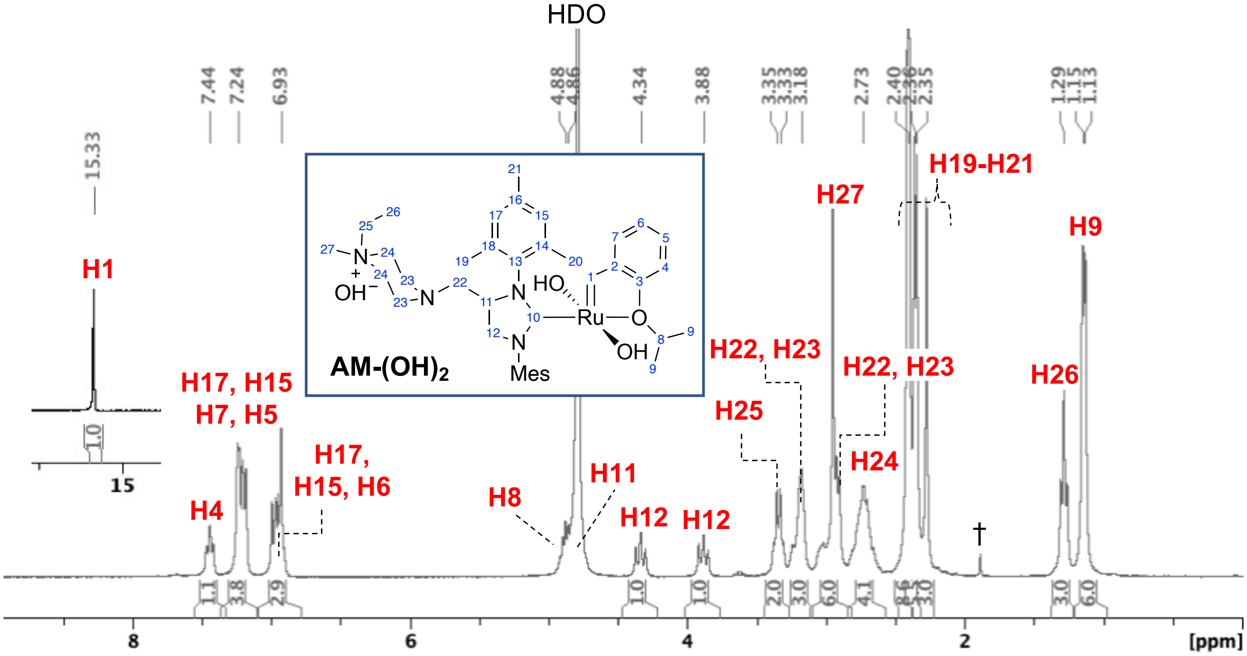


(b) $^{13}$C{$^{1}$H} NMR spectrum of **AM(OH)$_2$**

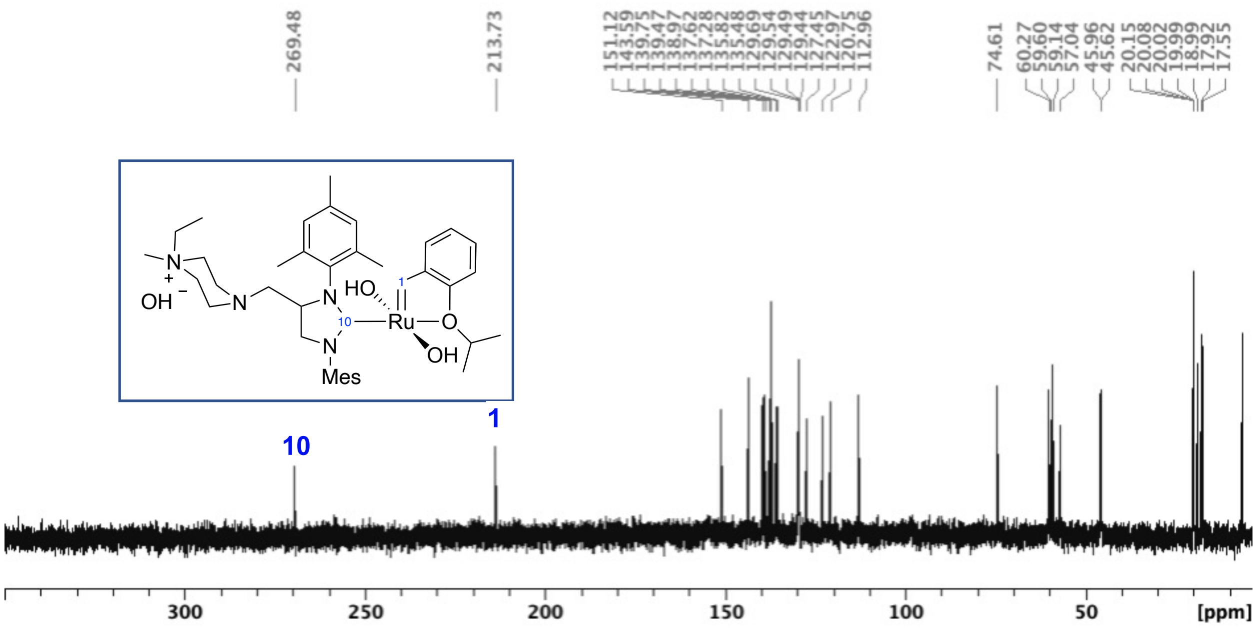

(c) $^1$H-$^1$H COSY NMR spectrum of **AM(OH)$_2$**

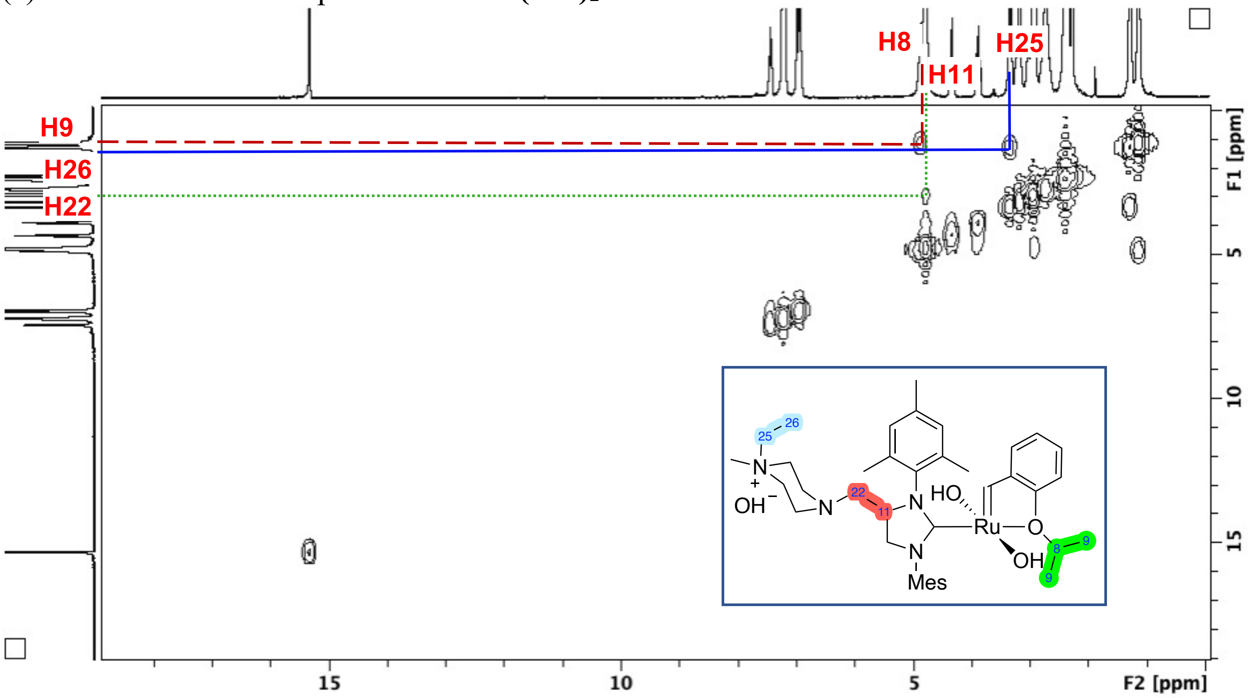


(d) $^1$H-$^{13}$C HSQC NMR spectrum of **AM(OH)$_2$**

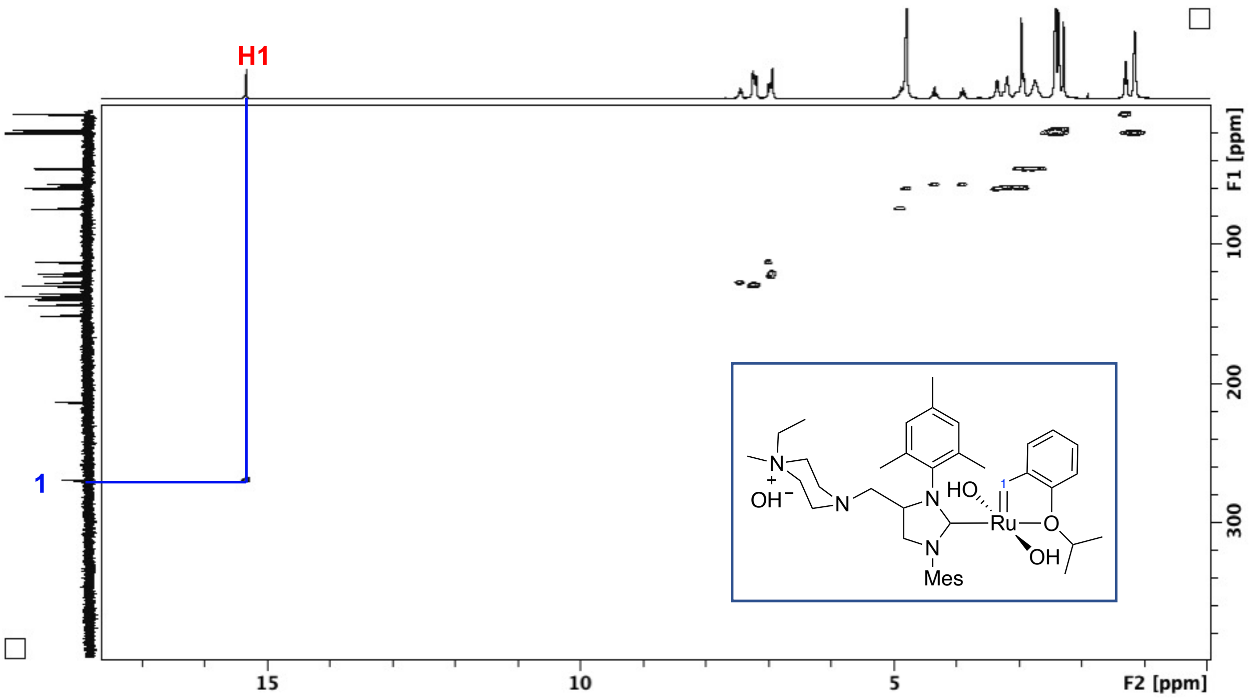

(e) $^1H$-$^{13}C$ HMBC NMR spectrum of **AM(OH)₂**

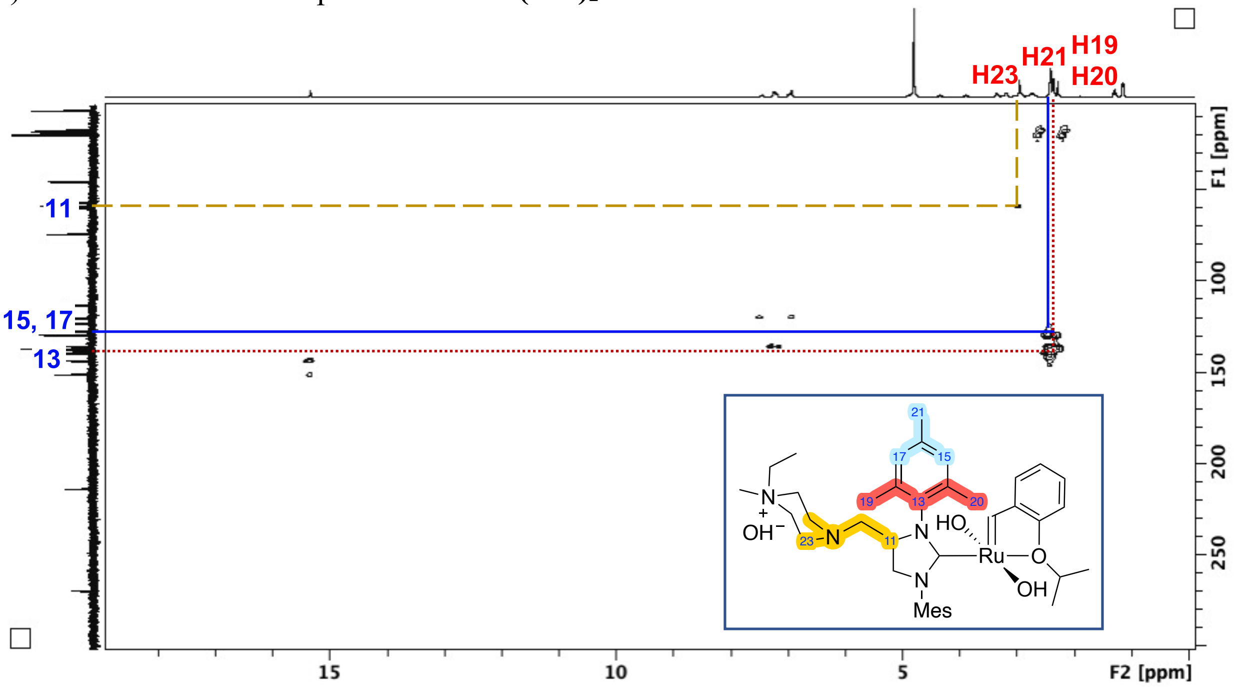


(f) $^1H$-$^1H$ NOESY NMR spectrum of **AM(OH)₂**

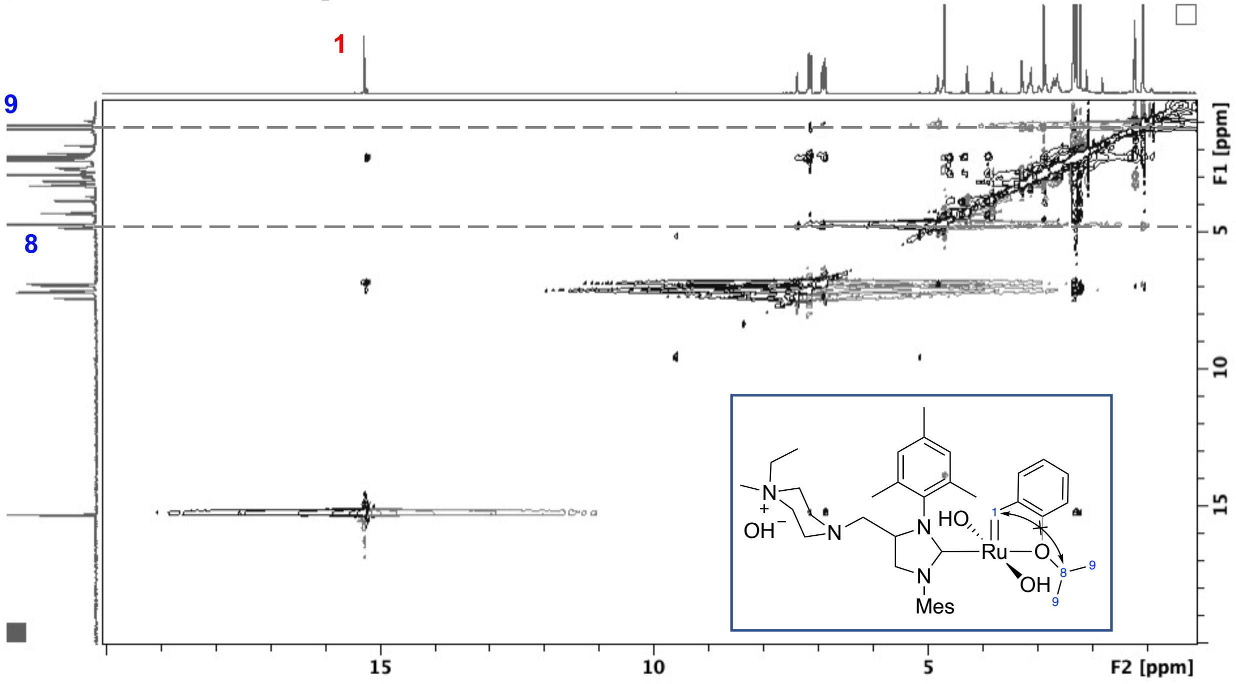


**Figure S2.** NMR characterization of **AM(OH)₂** in $D_2O$. (a) $^1H$ NMR (300 MHz); inset shows alkylidene signal. (b) $^{13}C\{^1H\}$ NMR (76 MHz). (c) $^1H$-$^1H$ COSY NMR (500 MHz). (d) $^1H$-$^{13}C$ HSQC NMR (300/76 MHz) showing correlation for alkylidene carbon. (e) $^1H$-$^{13}C$ HMBC NMR (300/76 MHz). (f) $^1H$-$^1H$ NOESY NMR (500 MHz) showing the absence of correlations between the alkylidene proton and the O$^i$Pr group, suggesting a chelate structure.

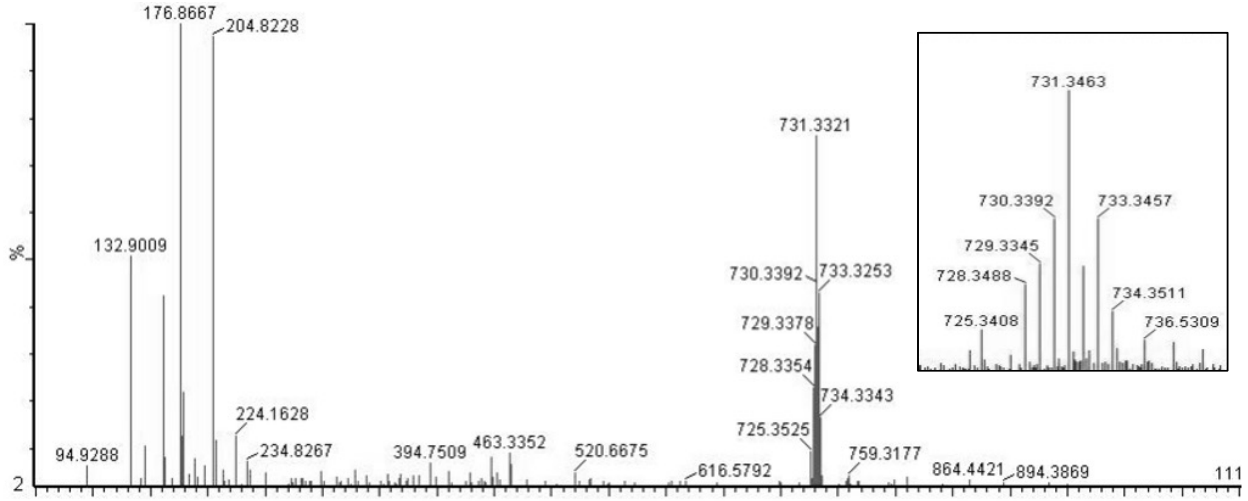


**Figure S3**. ESI-MS spectrum of **AM(OH)$_2$** in $H_2O$. (Inset shows the signal for **AM(OH)$_2$** after calibration with CsI).

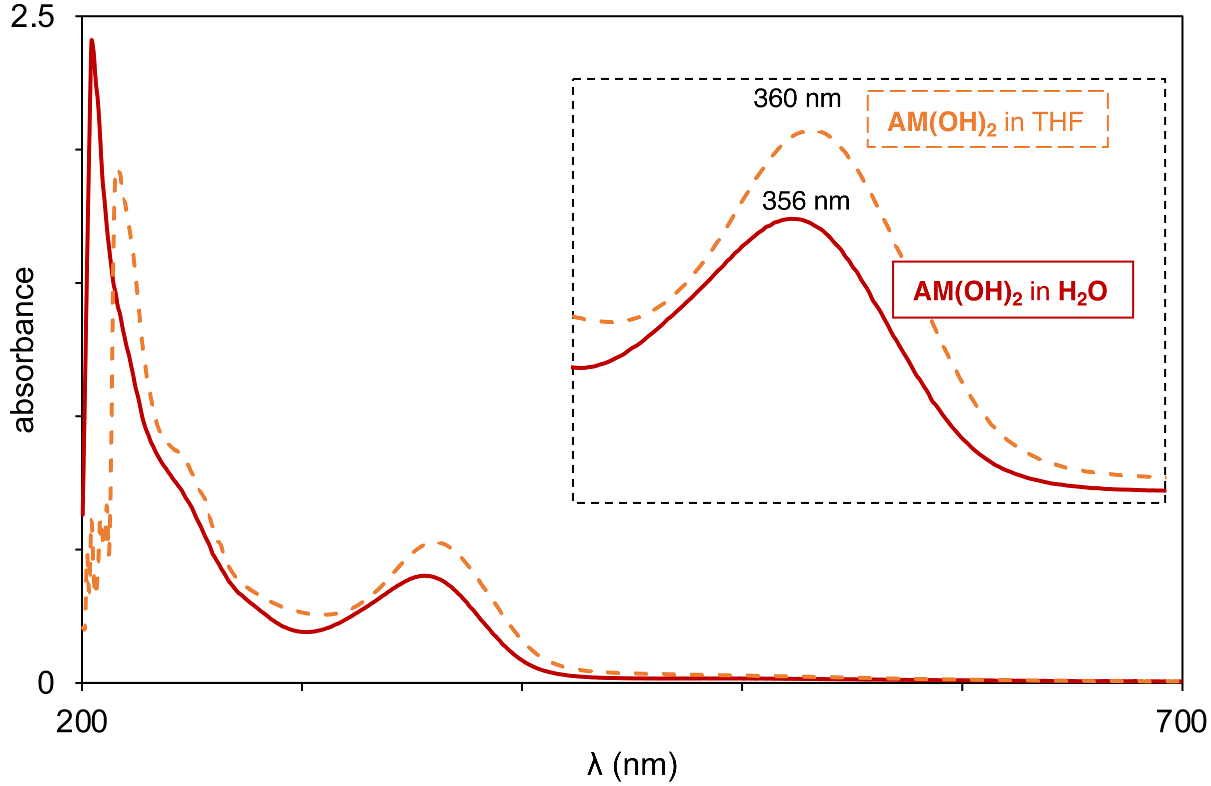


**Figure S4**. UV-vis spectra for **AM(OH)$_2$** in neutral $H_2O$ and THF. Inset shows the principal absorption band ($\lambda_{max}$).

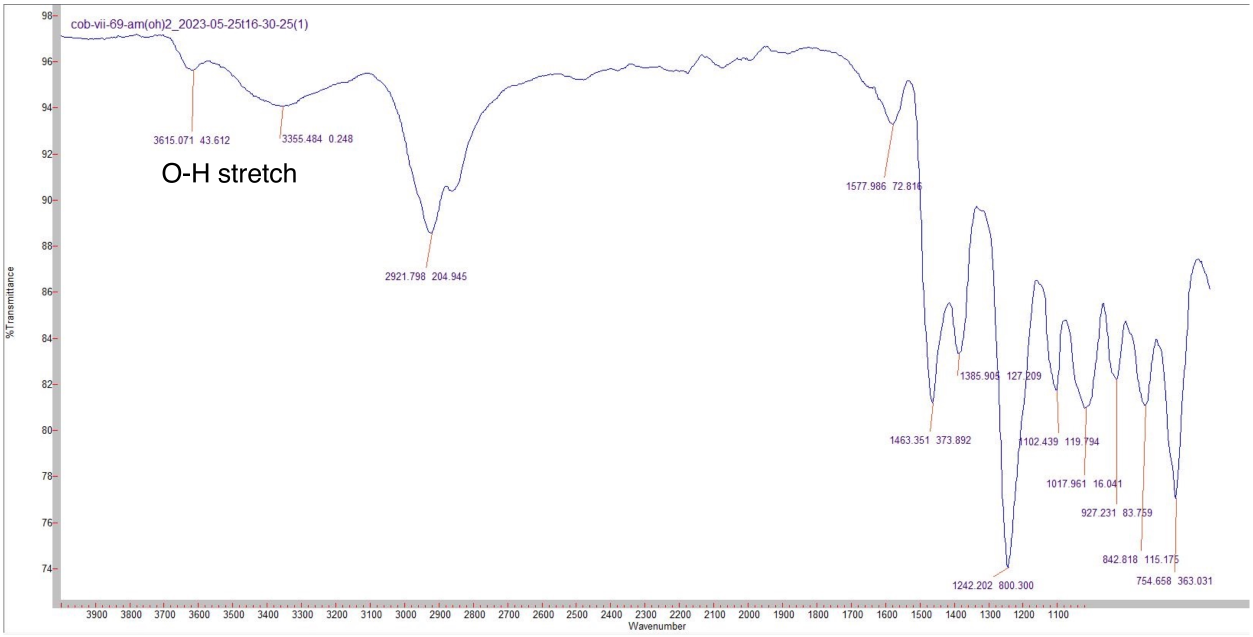


**Figure S5**. ATR-IR spectrum of **AM(OH)$_2$**.

(a) $^{1}$H NMR spectrum of **[AM(MeCN)$_3$](PF$_6$)$_3$**

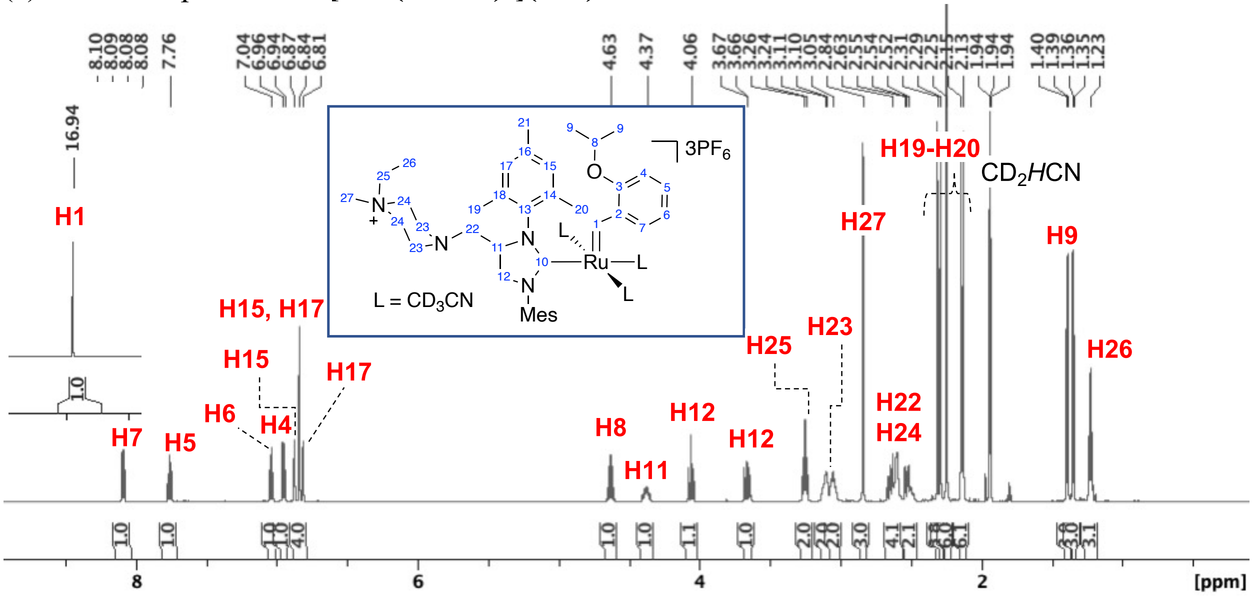


(b) $^{13}$C{$^{1}$H} NMR spectrum of **[AM(MeCN)$_3$](PF$_6$)$_3$**

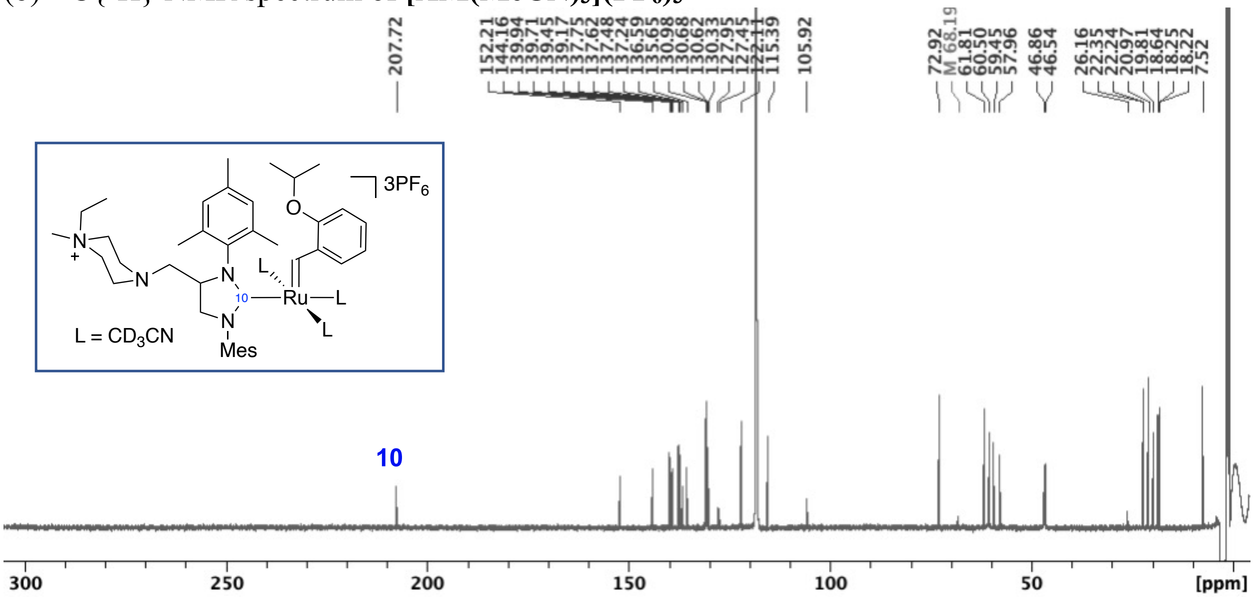

(c) $^{1}$H-$^{1}$H COSY NMR spectrum of **[AM(MeCN)$_3$](PF$_6$)$_3$**

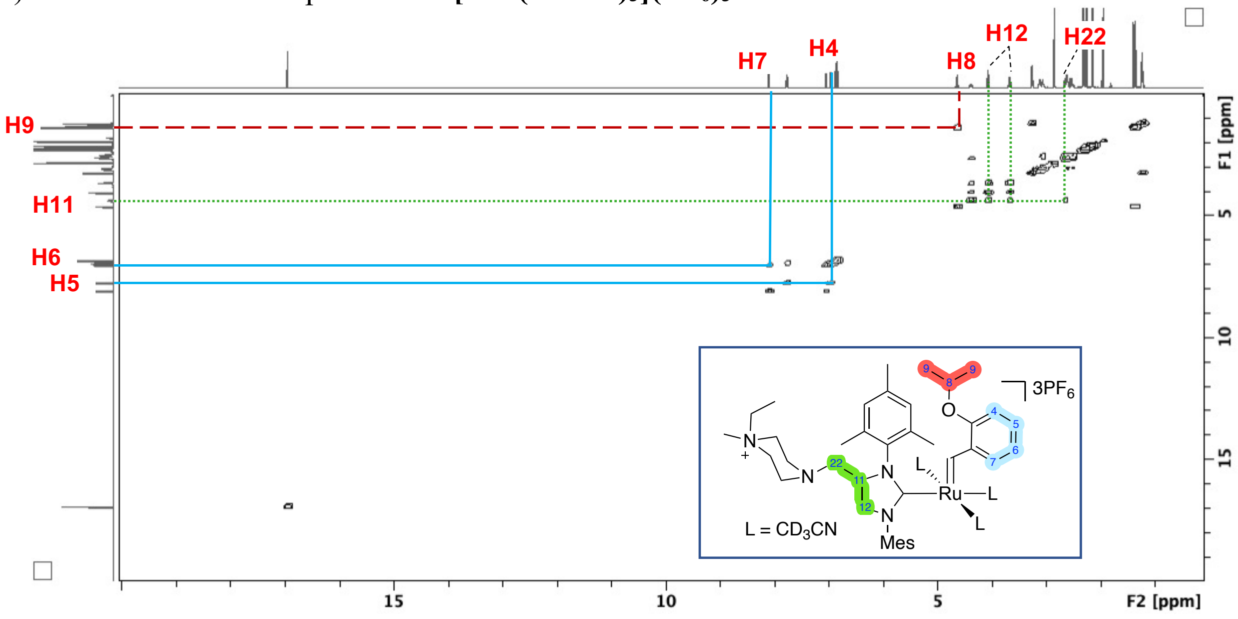


(d) $^{1}$H-$^{13}$C HSQC NMR spectrum of **[AM(MeCN)$_3$](PF$_6$)$_3$**

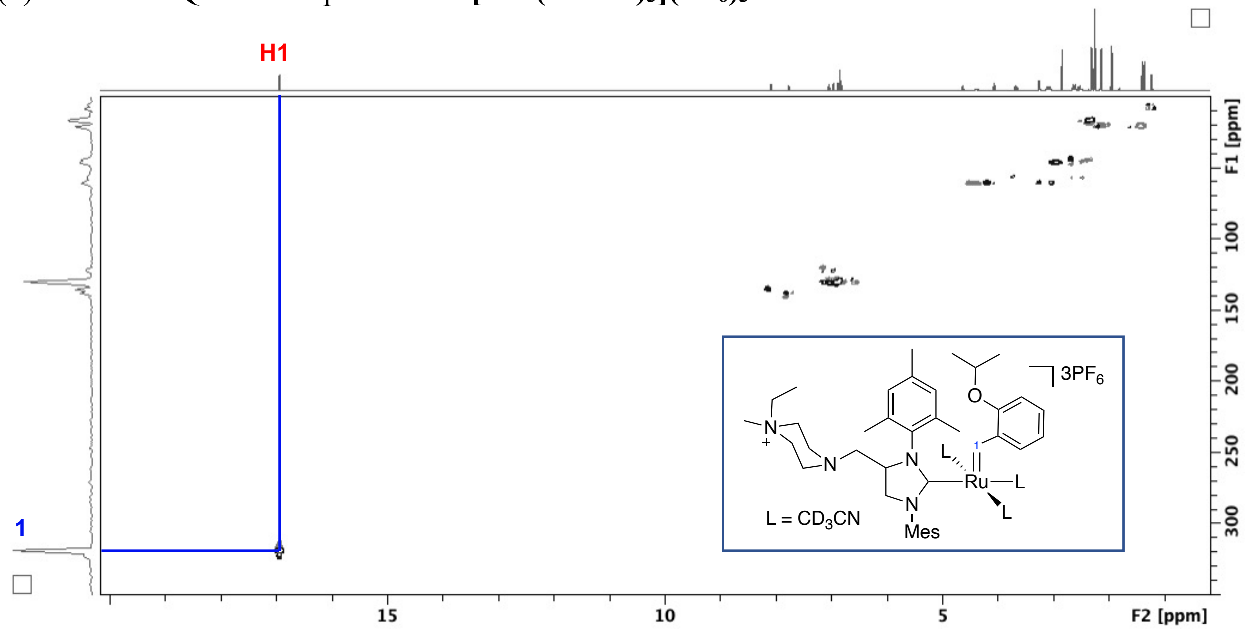

(e) $^{1}H$-$^{13}C$ HMBC NMR spectrum of **[AM(MeCN)3](PF6)3**

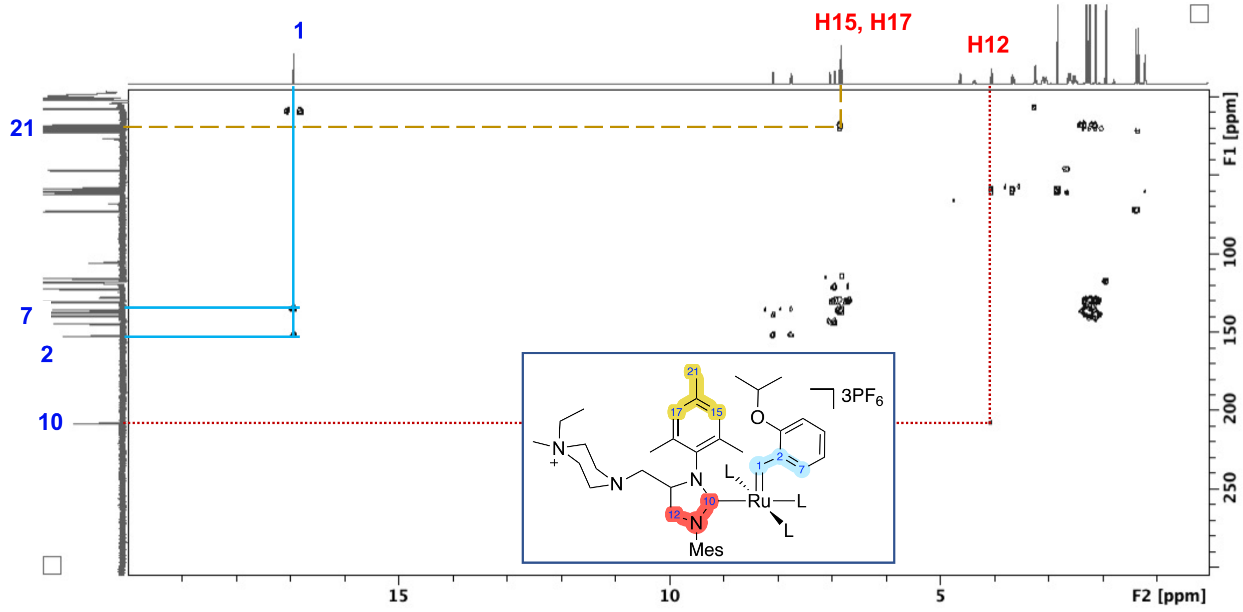


(f) $^{1}H$-$^{1}H$ NOESY NMR spectrum of **[AM(MeCN)3](PF6)3**

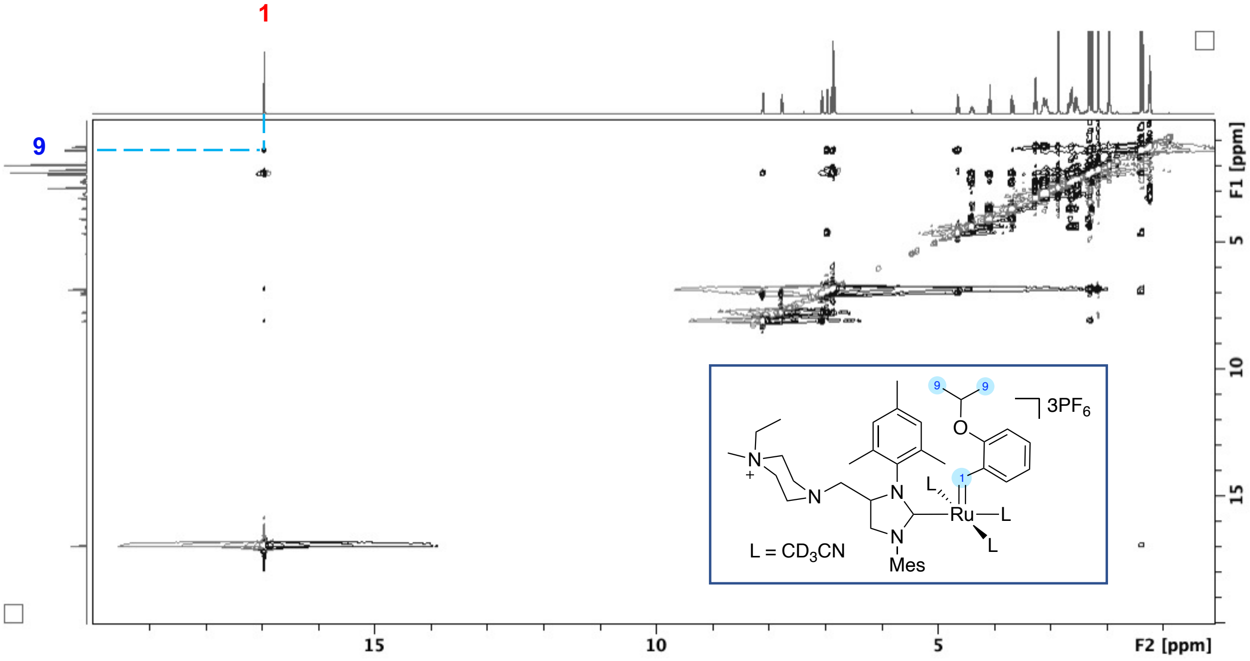


**Figure S6.** NMR characterization of **[AM(MeCN)3](PF6)3** in $CD_3CN$. (a) $^{1}H$ NMR (600 MHz); inset shows alkylidene signal. (b) $^{13}C\{^{1}H\}$ NMR (151 MHz). (c) $^{1}H$-$^{1}H$ COSY NMR (600 MHz). (d) $^{1}H$-$^{13}C$ HSQC NMR (600/151 MHz) showing correlation for alkylidene carbon. (e) $^{1}H$-$^{13}C$ HMBC NMR (600/151 MHz). (f) $^{1}H$-$^{1}H$ NOESY NMR (600 MHz) showing correlation of O$^{i}$Pr $CH_3$ protons to alkylidene proton which indicates dechelation.

(a) $^{1}$H NMR spectrum of **[AM(MeCN)$_3$](PF$_6$)$_3$** in $CD_2Cl_2$ with 20 equiv MeCN added

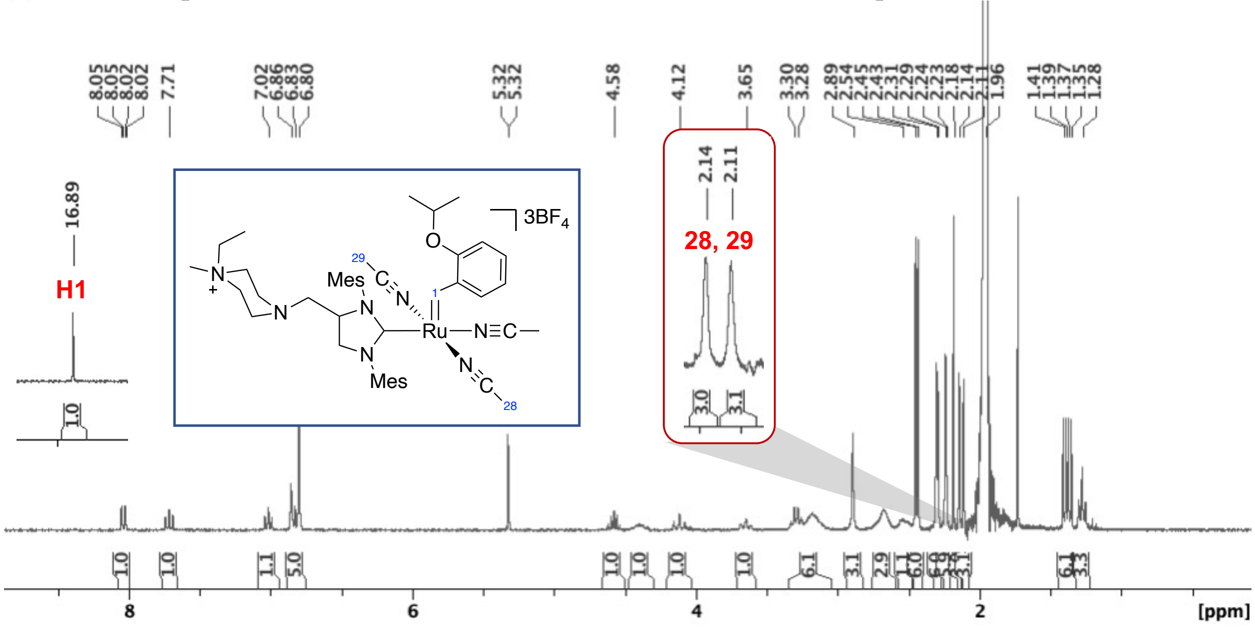


(b) $^{1}$H NMR spectrum of **[AM(MeCN)$_3$](PF$_6$)$_3$** in $CD_2Cl_2$

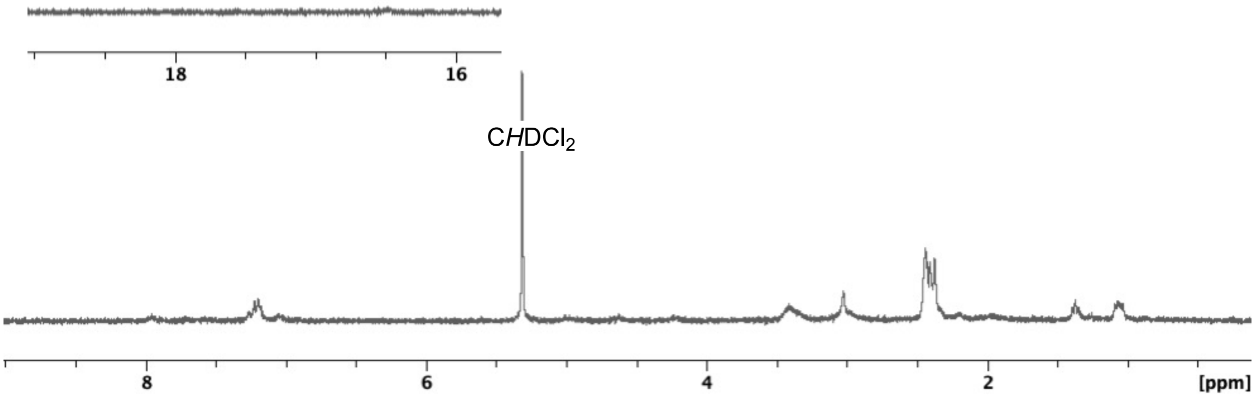


**Figure S7.** NMR spectra of **[AM(MeCN)$_3$](PF$_6$)$_3$** ($CD_2Cl_2$, 600 MHz). (a) With 20 equiv MeCN. The inset to the left shows the downfield alkylidene signal; that to the right shows expansion of the signals from the bound MeCN ligands. The third MeCN molecule was not observed. (b) Without MeCN. Inset shows null signal in the alkylidene region, indicating decomposition in the absence of MeCN.

(a) $^{1}$H NMR spectrum of Ru-aqua species generated on treating **AM** with $AgPF_6$

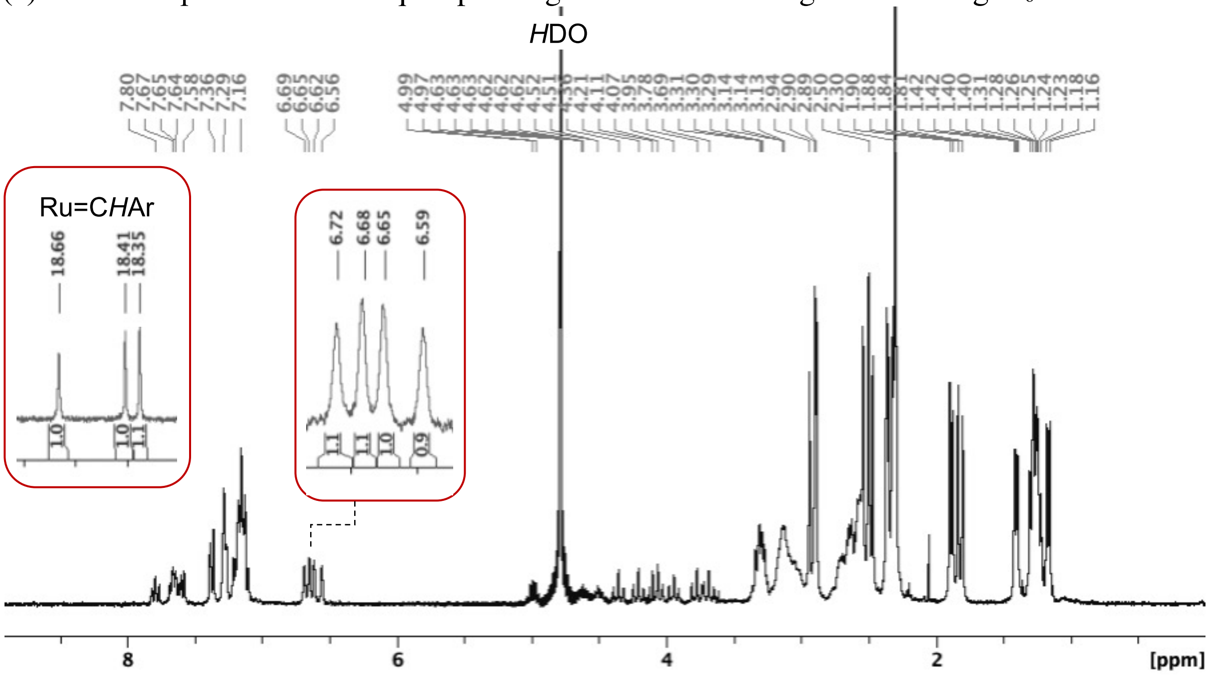


(b) $^{1}$H-$^{1}$H NOESY/EXSY NMR spectrum of $AgPF_6$-generated aqua species

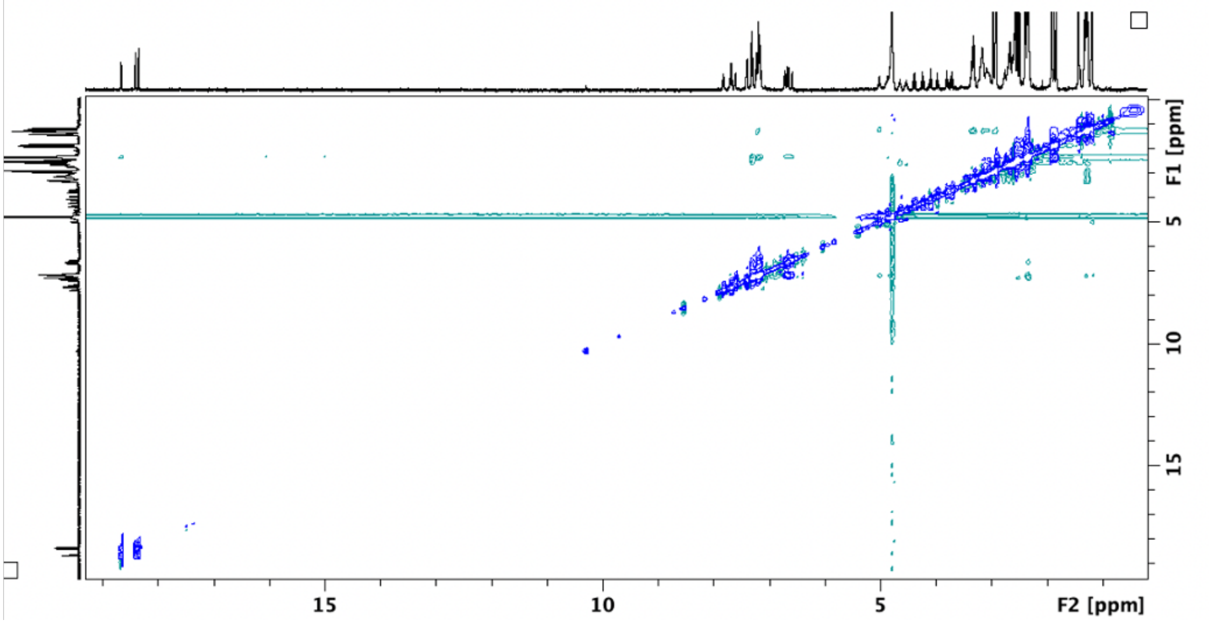

(c) $^{1}H$-$^{1}H$ NOESY/EXSY NMR spectrum of $AgPF_6$-generated aqua species, showing alkylidene region

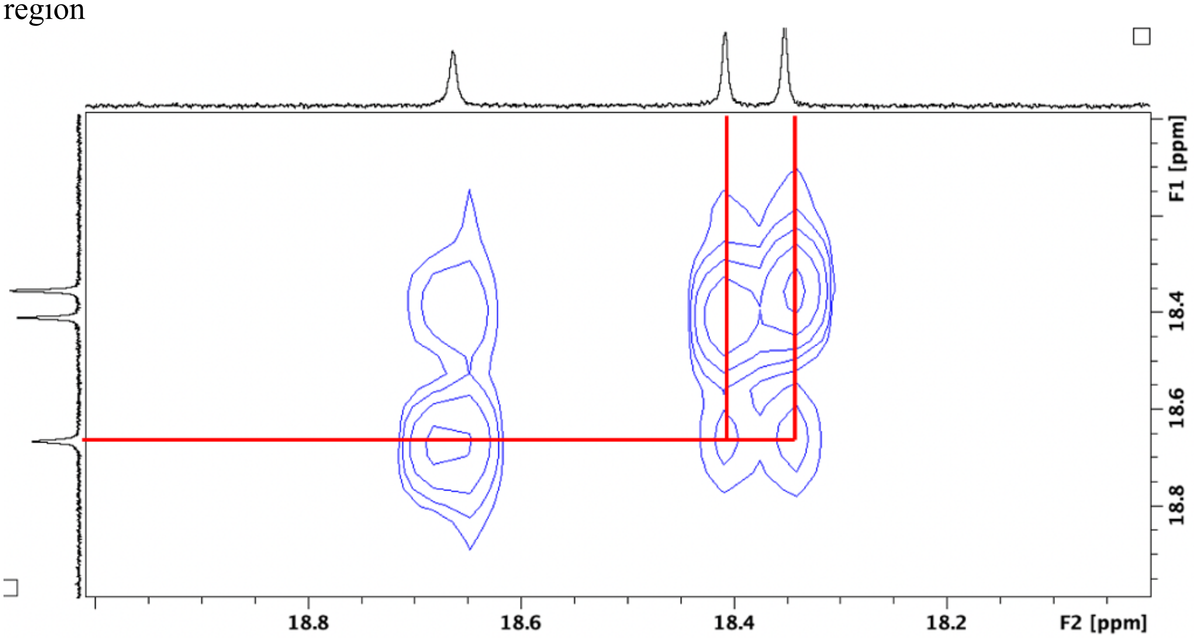


(d) UV-Vis spectrum of $AgPF_6$-generated aqua species

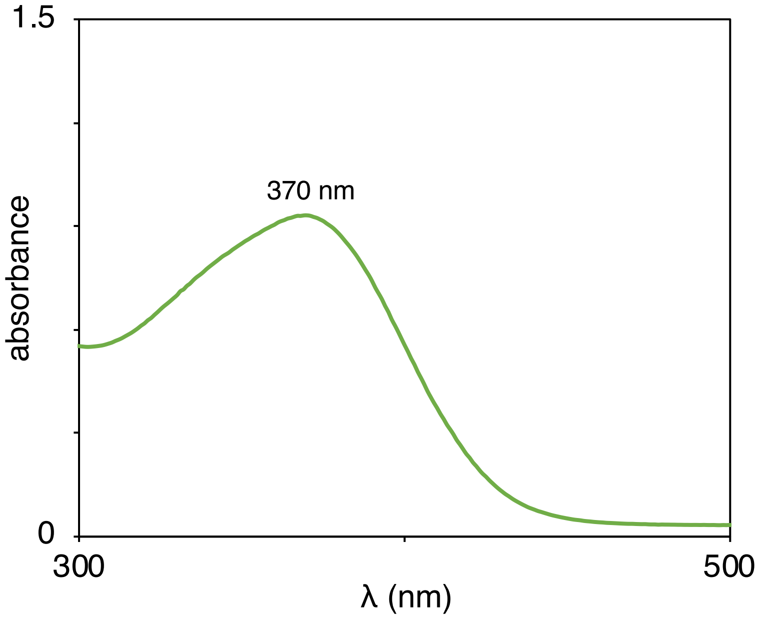


**Figure S8.** (a) $^{1}H$ NMR spectra after the $AgPF_6$-mediated generation of aqua species (300 MHz, $D_2O$; inset shows alkylidene signal). (b) $^{1}H$-$^{1}H$ NOESY NMR (500 MHz, 5 ºC, 2.0 relaxation delay). (c) Expansion showing exchange correlations between signals in the alkylidene region. (d) UV-vis spectrum in $H_2O$.

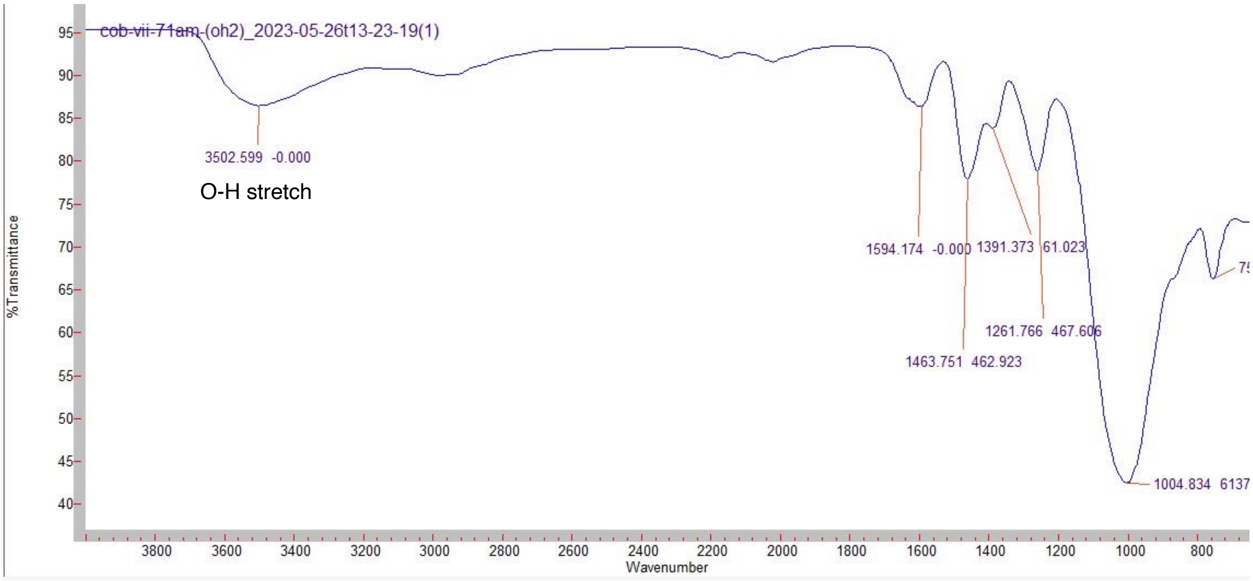


**Figure S9**. ATR-IR spectrum of $AgPF_6$-generated aqua species after lyophilization.

(a) DOSY NMR for Ru-aqua species generated on treating **AM** with $AgPF_6$

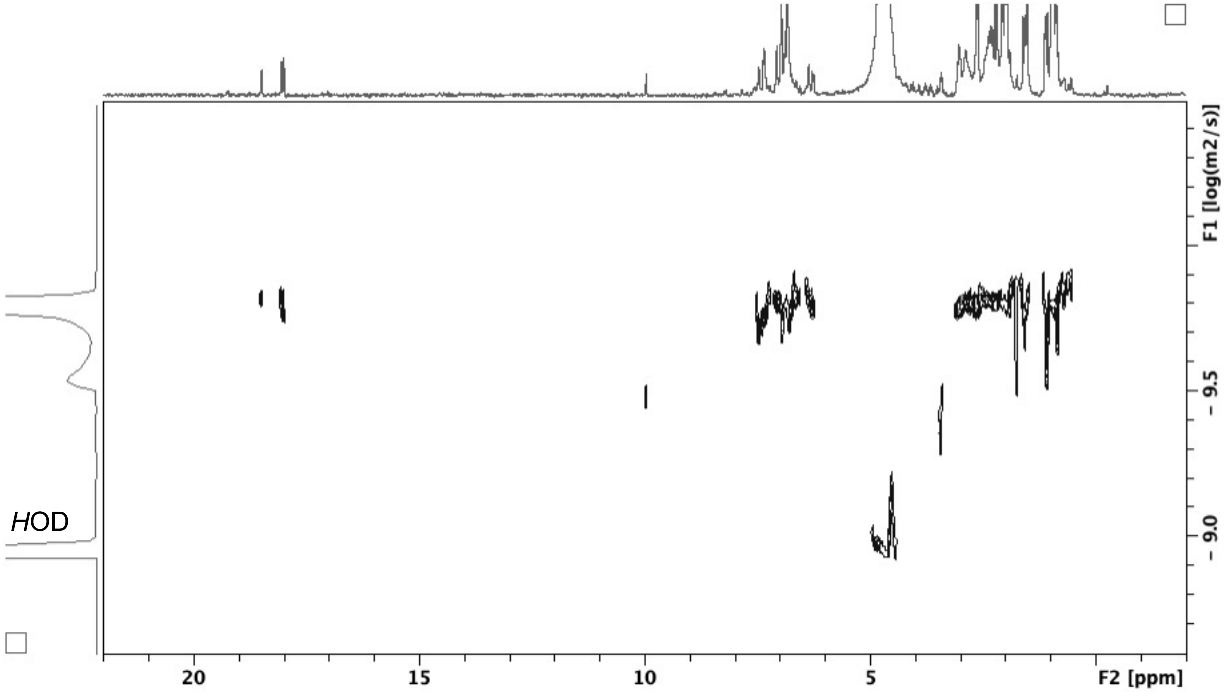


(b) Diffusion constants for Ru-aqua species generated on treating **AM** with $AgPF_6$

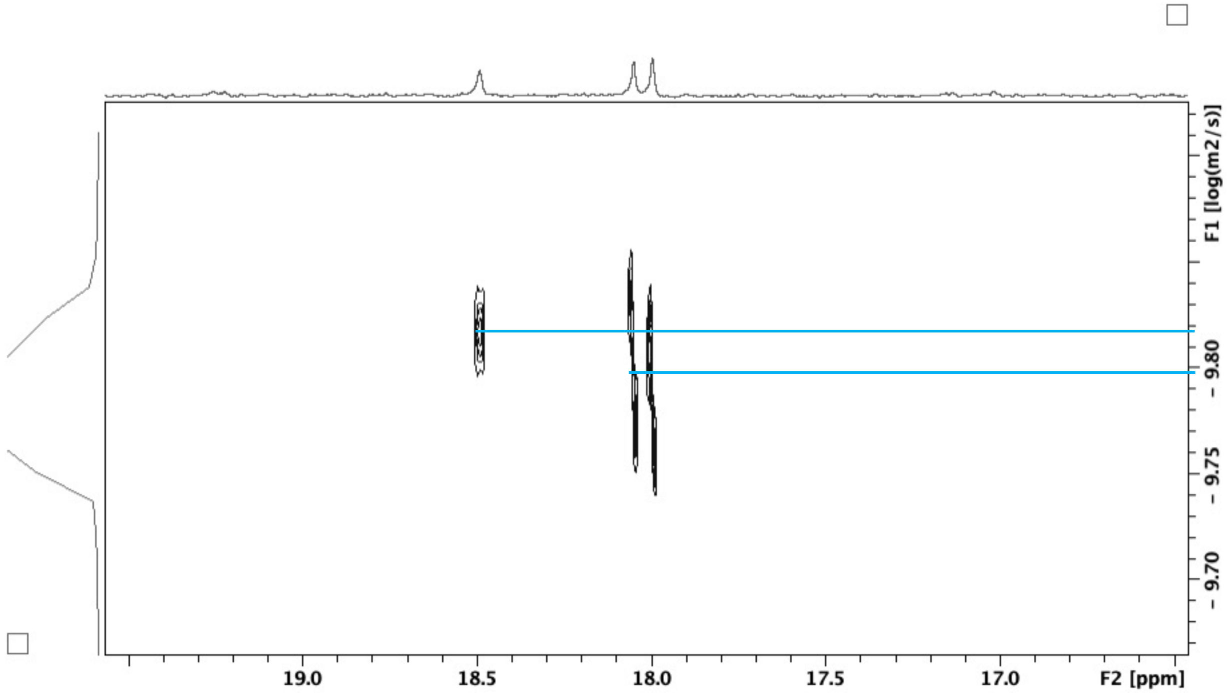


**Figure S10.** (a) DOSY-NMR spectrum for Ru-aqua species generated on treating **AM** with $AgPF_6$ (10 mM in $D_2O$; 300 MHz). (b) Expansion of alkylidene region showing diffusion constants.

(a) Ru-aqua species generated on treating **AM** with 3 equiv $AgBF_4$

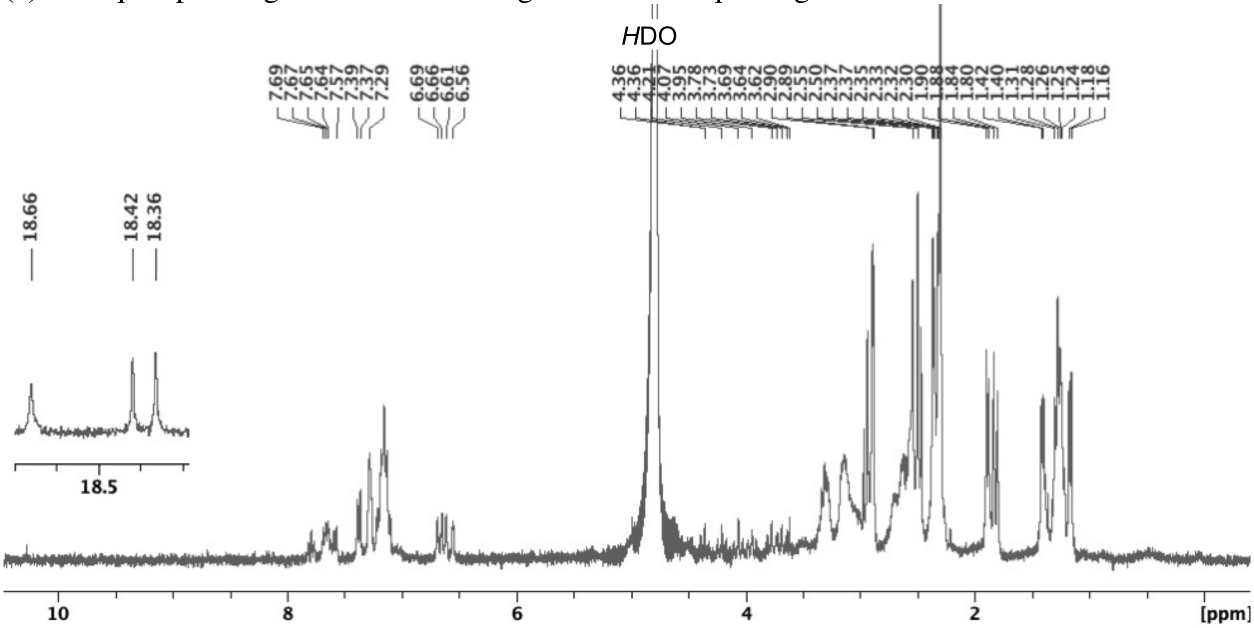


(a) Ru-aqua species generated on treating **AM** with 5 equiv $AgBF_4$

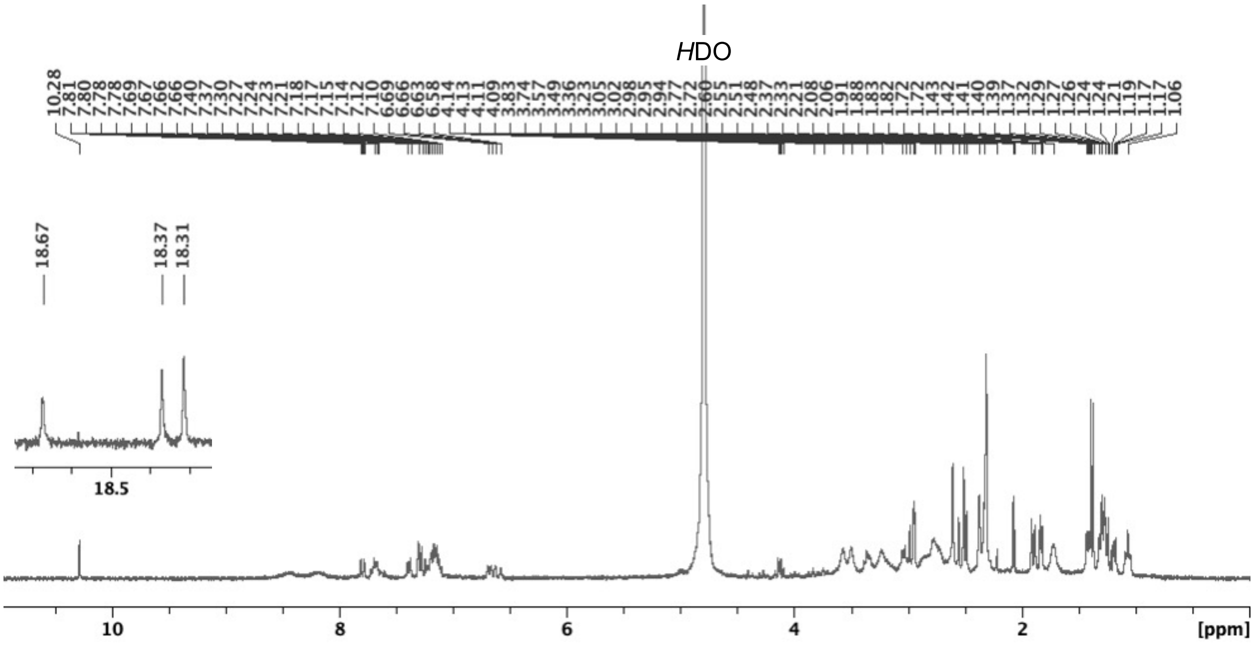


**Figure S11.** $^1$H NMR spectra of Ru-aqua species generated on treating **AM** with $AgBF_4$ at RT (300 MHz, $D_2O$). Insets show downfield alkylidene signal. (a) Experiment with 3 equiv $AgBF_4$. (b) Experiment with 5 equiv $AgBF_4$. The similarity illustrates complete chloride abstraction with 3 equiv of the silver salt.

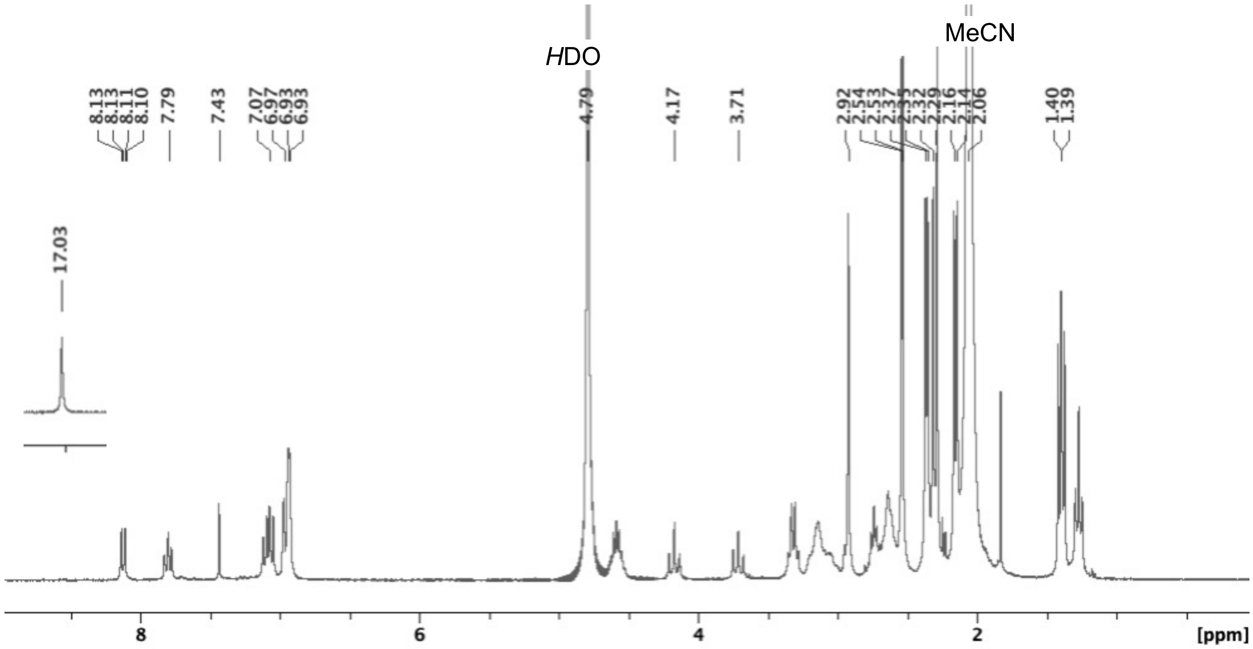


**Figure S12.** $^1$H NMR spectra after addition of MeCN to $AgPF_6$-generated aqua species (300 MHz, $D_2O$; inset shows downfield alkylidene signal).

(a) $CD_2Cl_2$

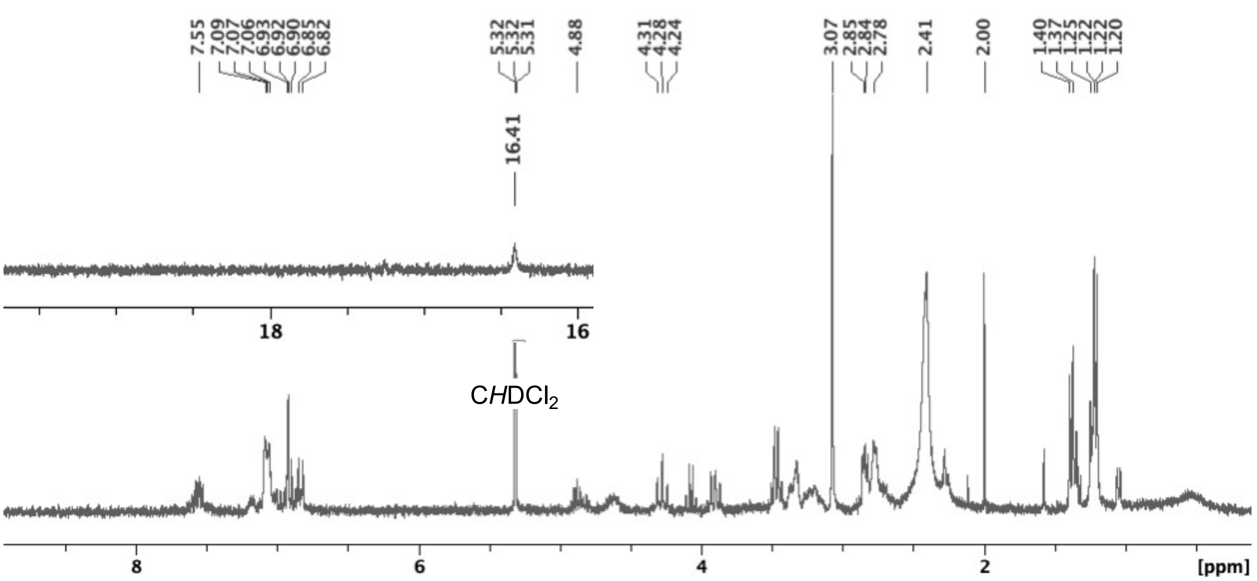


(b) $CD_3OD$

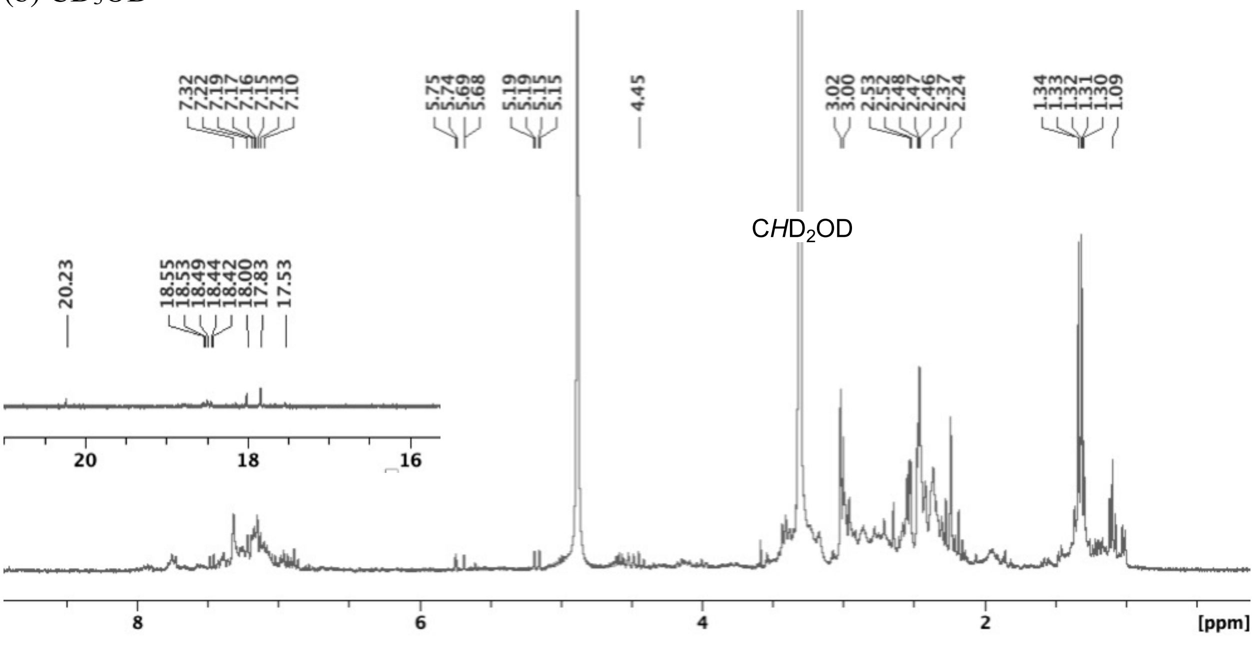


**Figure S13.** (a) $^1H$ NMR spectra (300 MHz) showing the immediate decomposition of the $AgPF_6$-generated aqua species on dissolving in the absence of MeCN. (a) In $CD_2Cl_2$. (b) In $CD_3OD$. Insets show null signals in the alkylidene region.

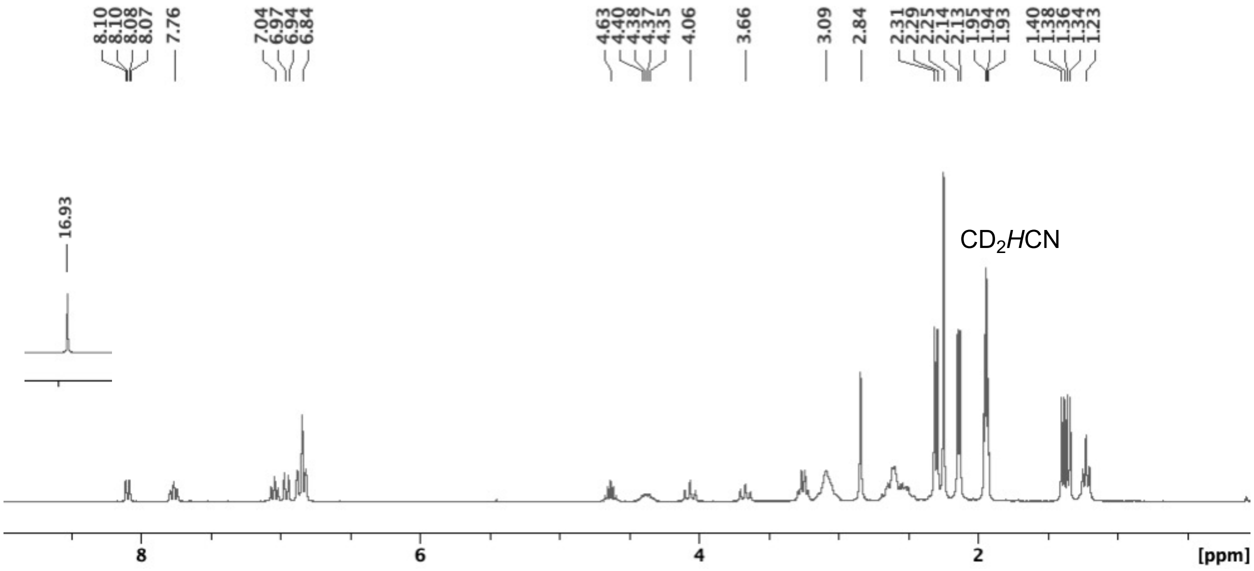


**Figure S14.** $^1H$ NMR spectra (600 MHz) after lyophilization of the $AgPF_6$-generated aqua species and dissolution in $CD_3CN$, showing complete formation of **[AM(MeCN)$_3$](PF$_6$)$_3$**.

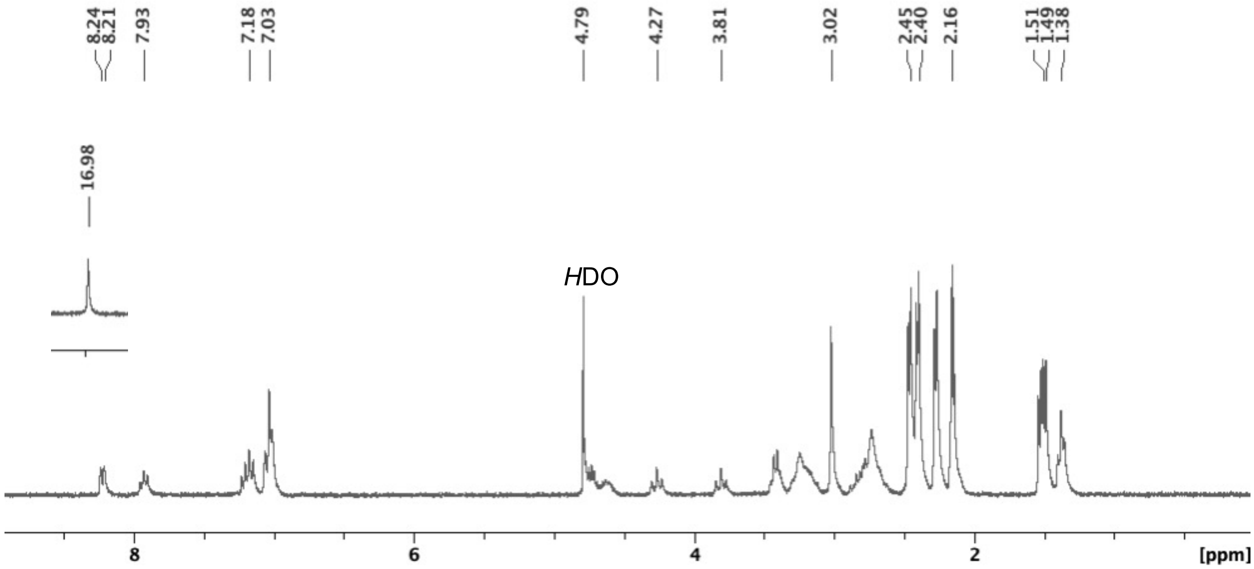


**Figure S15.** $^1$H NMR spectra (300 MHz, $D_2O$) of **[AM(MeCN)$_3$](PF$_6$)$_3$**.

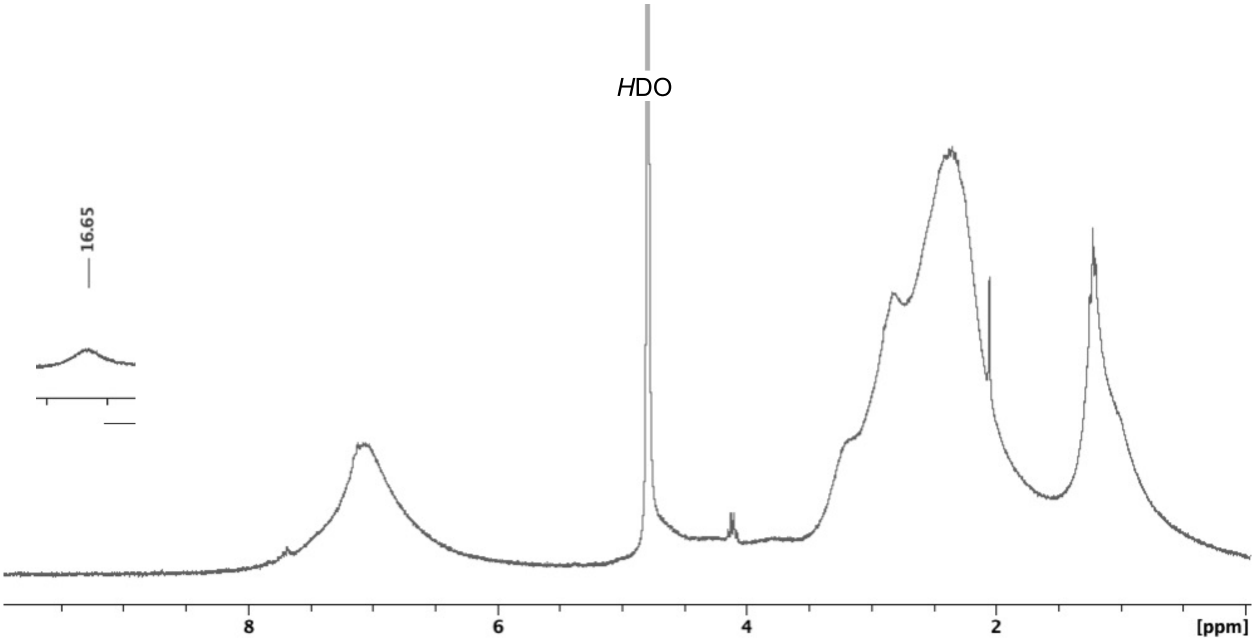


**Figure S16.** $^1$H NMR spectrum (300 MHz) of **AM** in $D_2O$, showing extreme signal broadening.

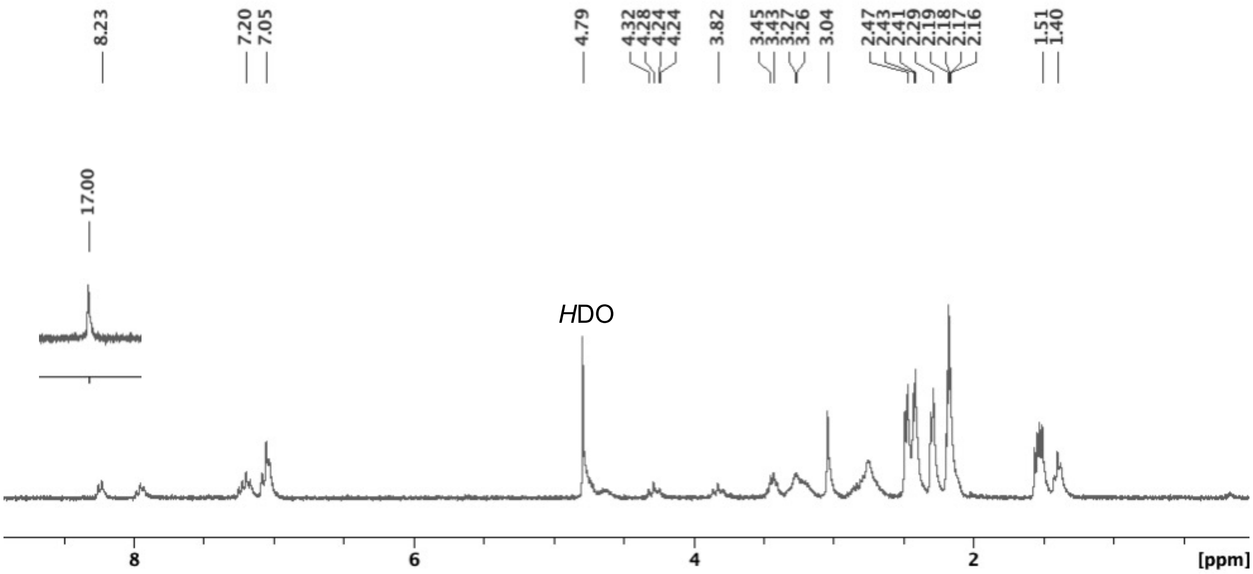


**Figure S17.** $^{1}$H NMR spectra (300 MHz, $D_2O$) after addition of 20 equiv $CD_3CN$ to **AM**, showing full conversion into **[AM(MeCN)$_3$]Cl$_3$**.

(a) $^{1}$H NMR spectrum of phosphonium salt **3**

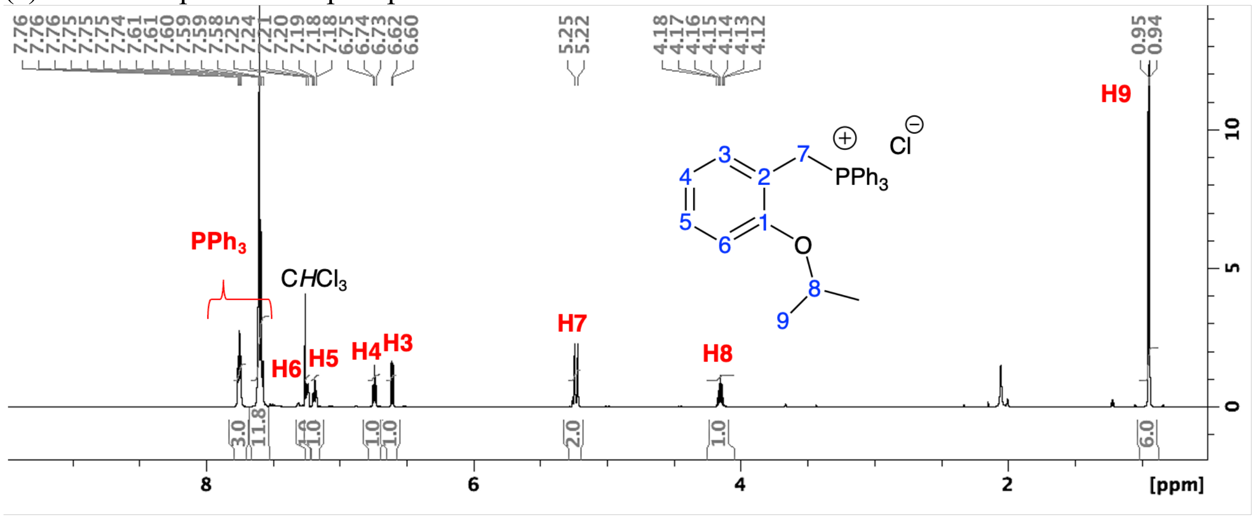


(b) $^{31}$C{$^{1}$H} NMR spectrum of **3**

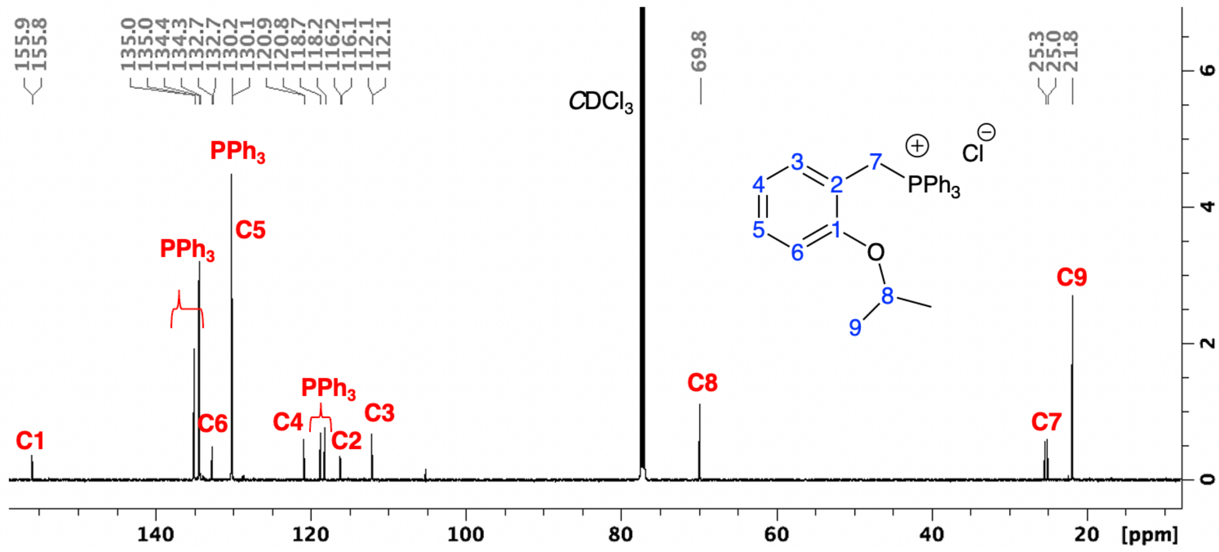

(c) $^{1}$H-$^{13}$C HSQC NMR spectra of **3**

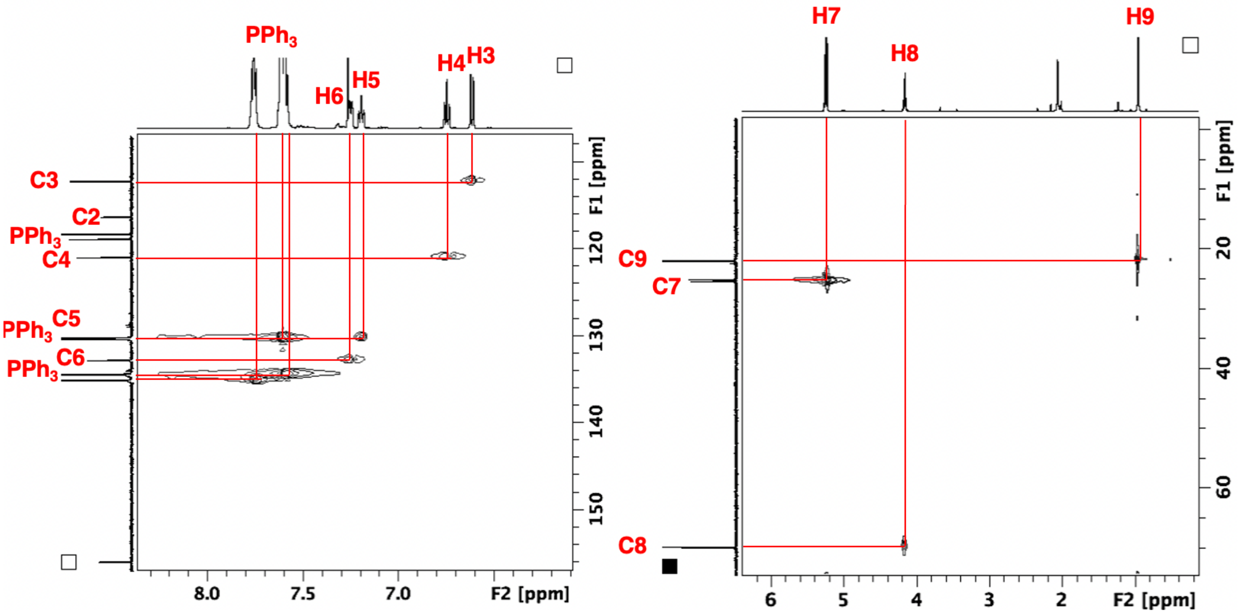


(d) $^{1}$H-$^{13}$C HMBC NMR spectrum of **3**

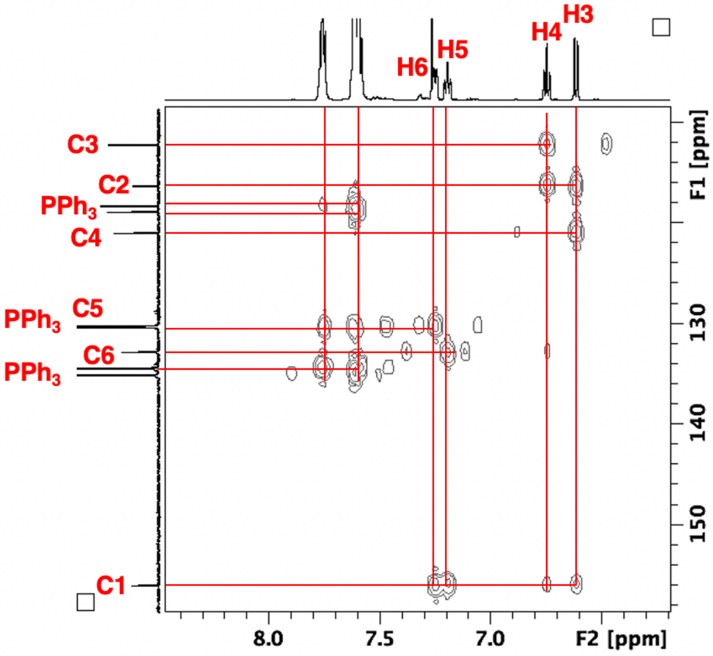

(e) $^{31}P\{^{1}H\}$ NMR spectrum of **3**

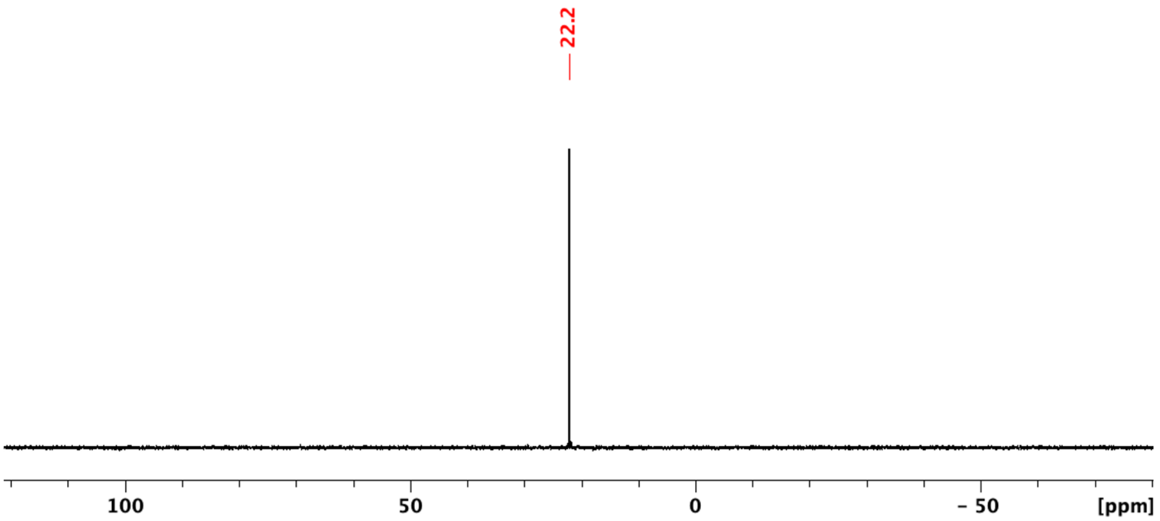


**Figure S18.** NMR characterization of phosphonium salt **3** in $CDCl_3$. (a) $^{1}H$ NMR (600 MHz). (b) $^{13}C\{^{1}H\}$ NMR (151 MHz). (c) $^{1}H$-$^{13}C$ HSQC NMR (600/151 MHz). (d) $^{1}H$-$^{13}C$ HMBC NMR (600/151 MHz). (e) $^{31}P\{^{1}H\}$ NMR (121 MHz).

(a)$^{1}$H NMR spectrum of stilbene **2**.

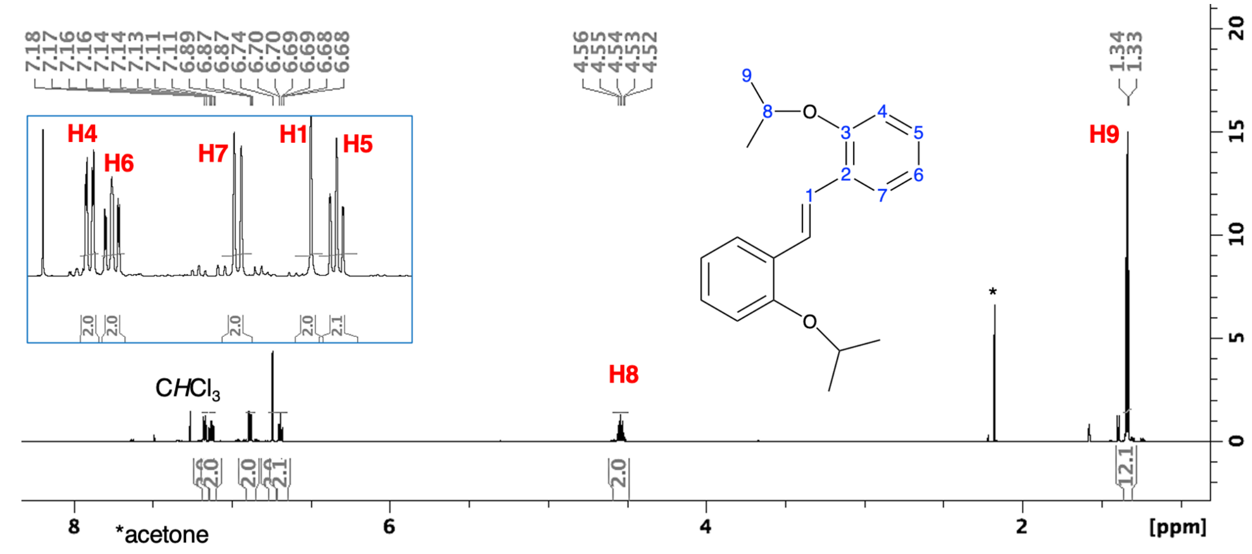


(b) $^{13}C\{^{1}H\}$ NMR spectrum of **2**.

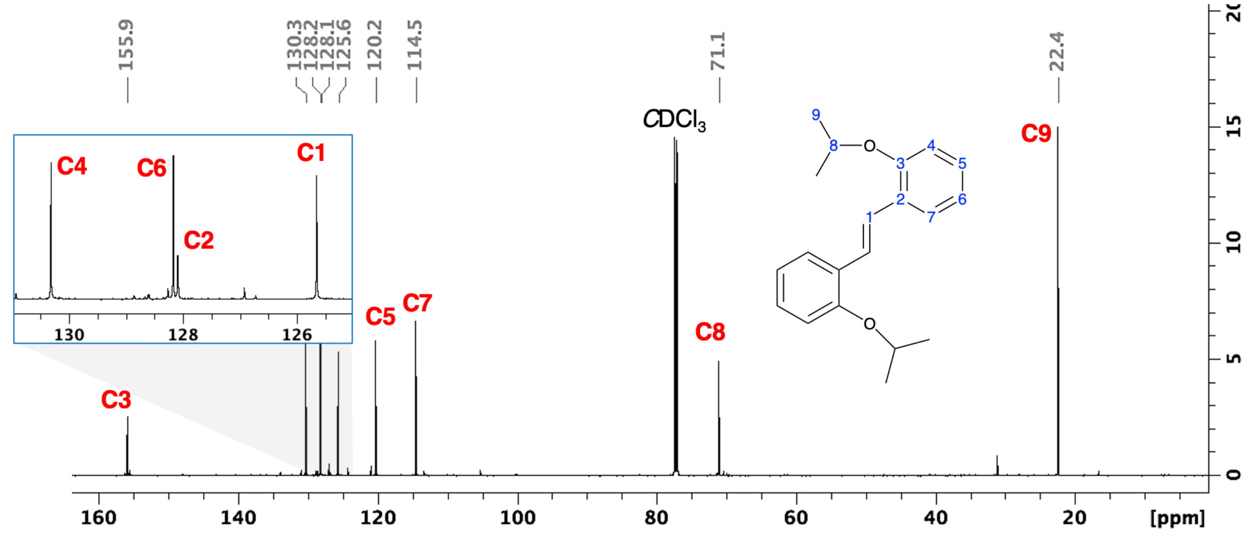

(c) $^{1}$H-$^{1}$H COSY NMR spectrum of stilbene **2**.

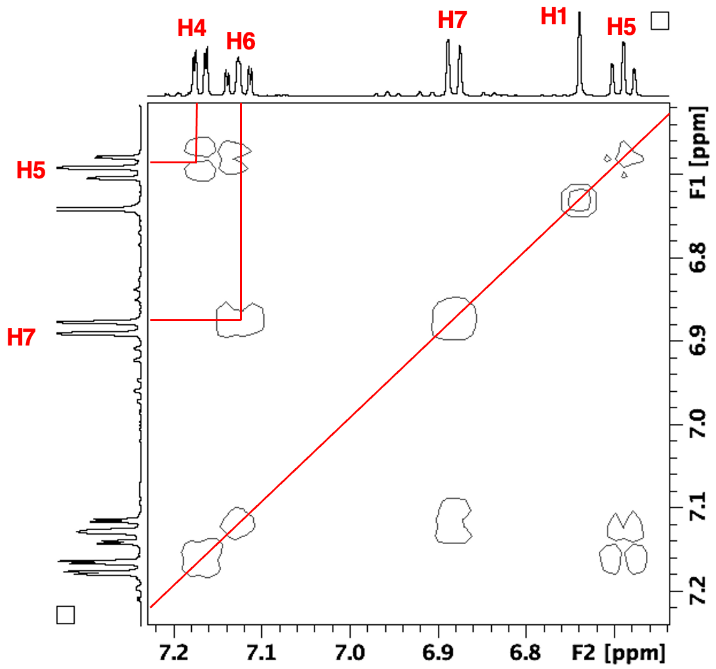


(d) $^{1}$H-$^{13}$C HSQC NMR spectrum of **2**.

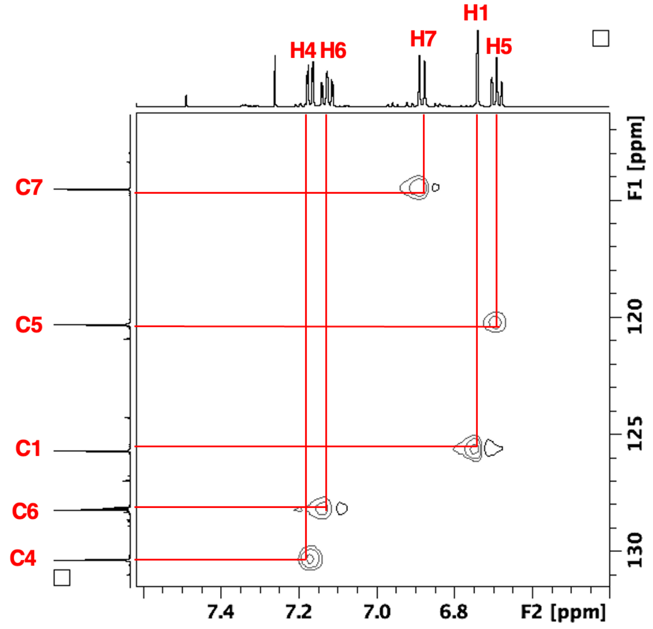

(e) $^{1}H$-$^{13}C$ HMBC NMR spectra of stilbene **2**.

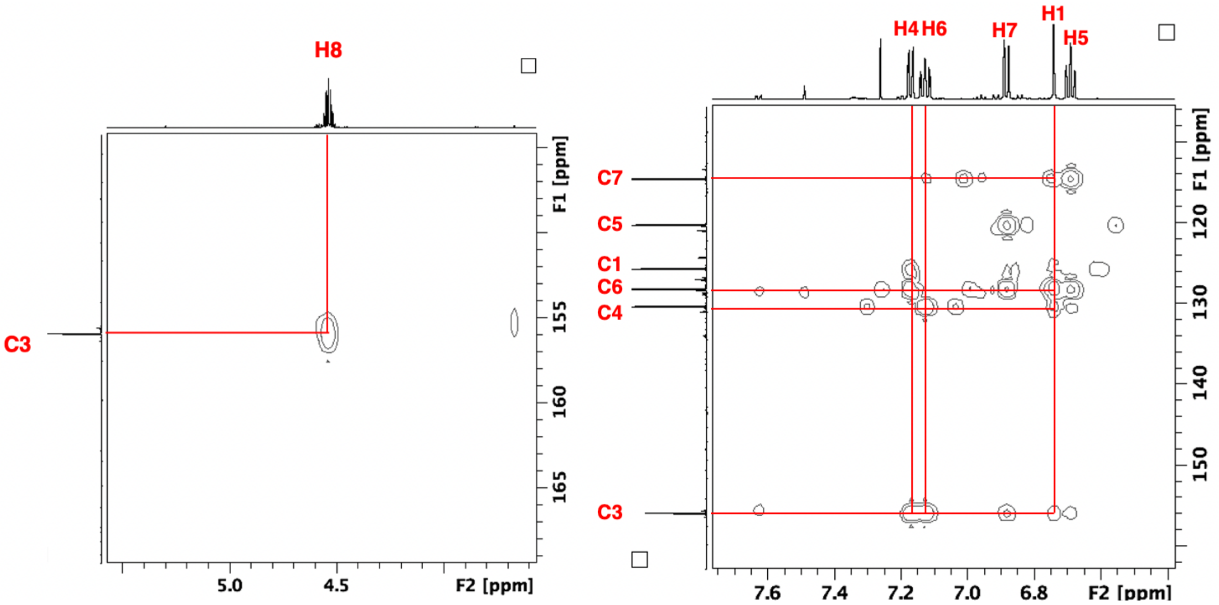


**Figure S19.** NMR characterization of stilbene **2** in $CDCl_3$. (a) $^{1}H$ NMR (600 MHz). (b) $^{13}C\{^{1}H\}$ NMR (151 MHz). (c) $^{1}H$-$^{1}H$ COSY NMR (600 MHz). (d) $^{1}H$-$^{13}C$ HSQC NMR (600/151 MHz). (e) $^{1}H$-$^{13}C$ HMBC NMR (600/151 MHz).

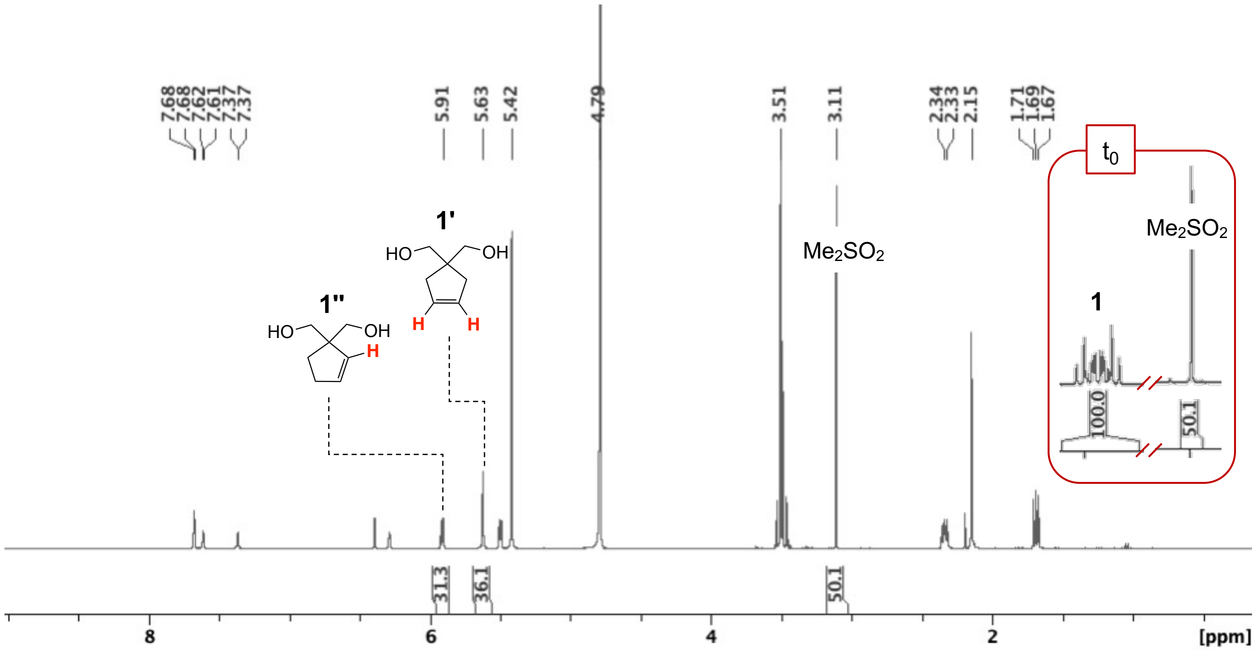


**Figure S20.** Quantifying RCM and isomerization of **1** by **AM** (2 mol%). Representative $^{1}$H NMR spectrum (400 MHz, $D_2O$) at 100% conversion. Internal standard (IS) = dimethylsulfone, $Me_2SO_2$.

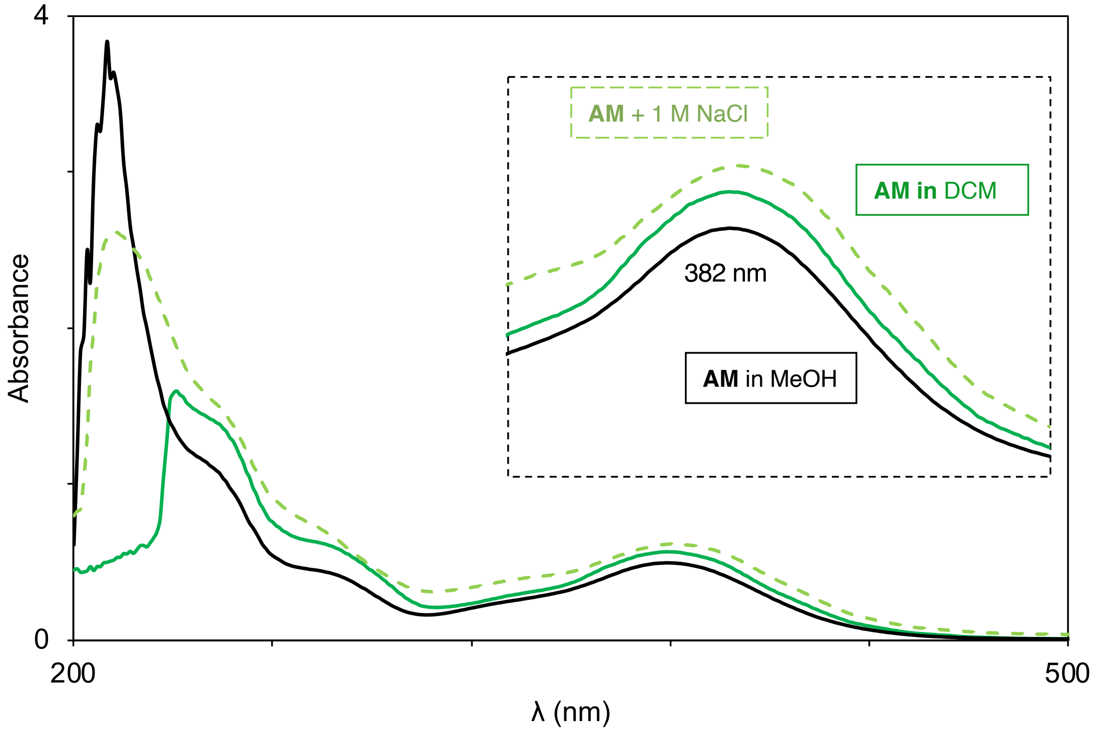


**Figure S21**.UV-vis spectra of **AM** in: $H_2O$ + 1.0 M NaCl, MeOH, and DCM showing the principal absorption band ($\lambda_{max}$) for the dichloride structure.

(a) 16 mM solution of **AM** after 3 days at 40 °C, showing no decomposition

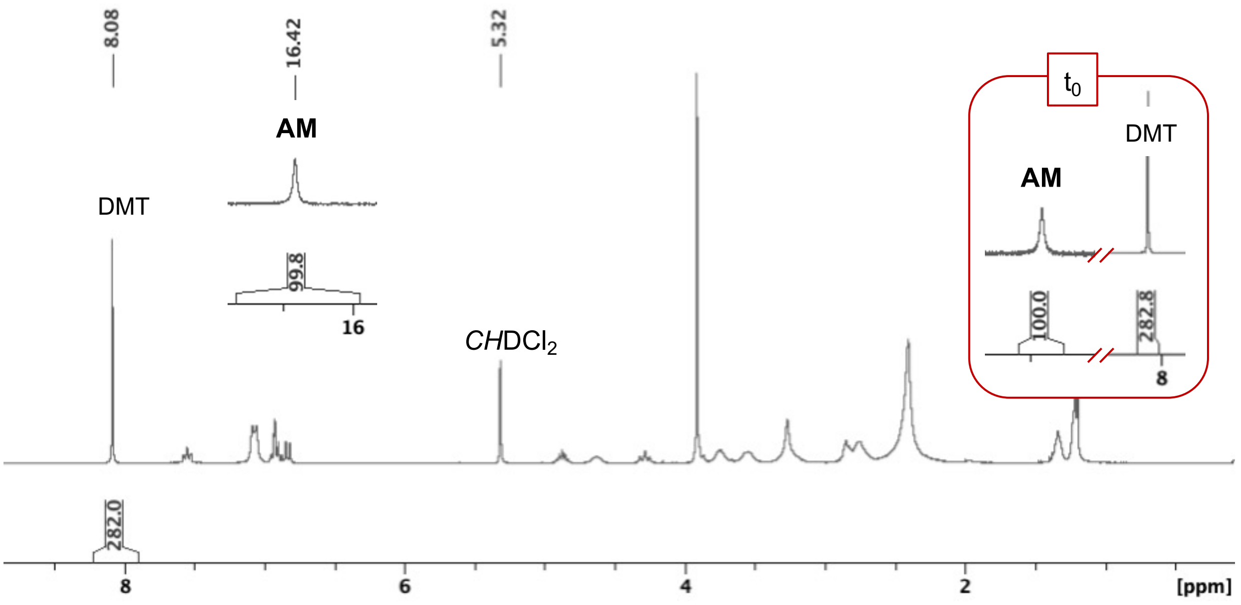


(b) 40 mM solution of **AM** after 3 days at 40 °C, showing no decomposition

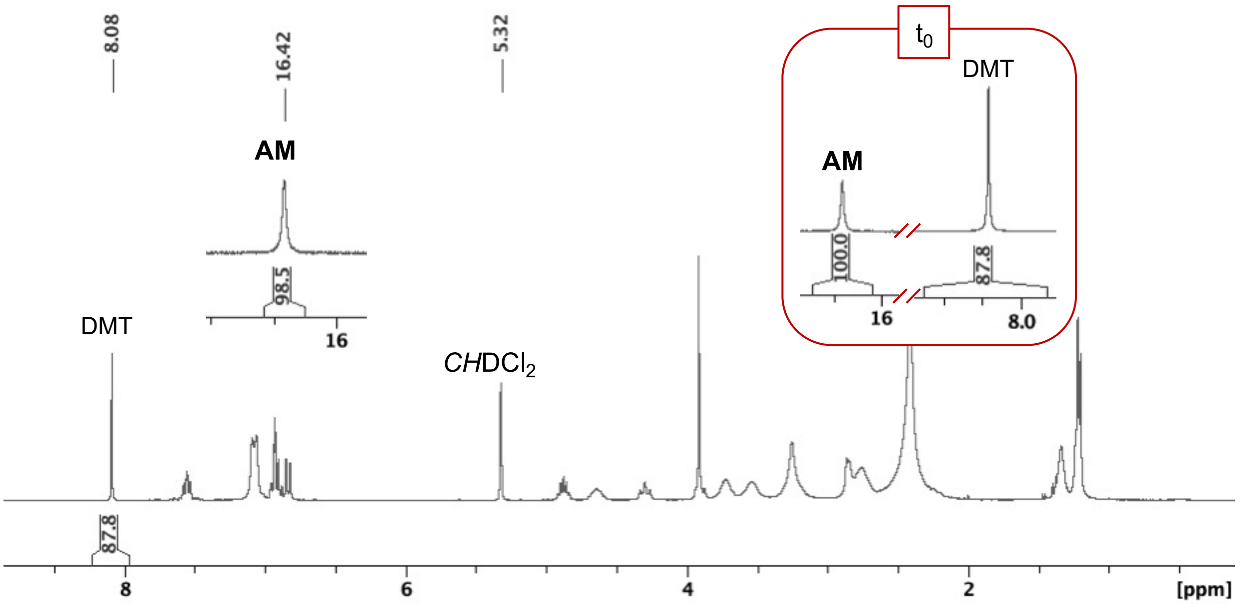


**Figure S22**. Stability of **AM** to thermolysis for 3 days at 40 ºC in $CD_2Cl_2$, assessed by $^{1}H$ NMR analysis (500 MHz). Integrations of the alkylidene signal (left inset) are normalized to that of **AM** at $t_0$ (inset at right). (a) Experiments at 16 mM **AM**. (b) Experiments at 40 mM **AM**.

(a) $^1$H NMR spectrum showing formation of trans-stilbenoid **2** on decomposition of **AM** in water

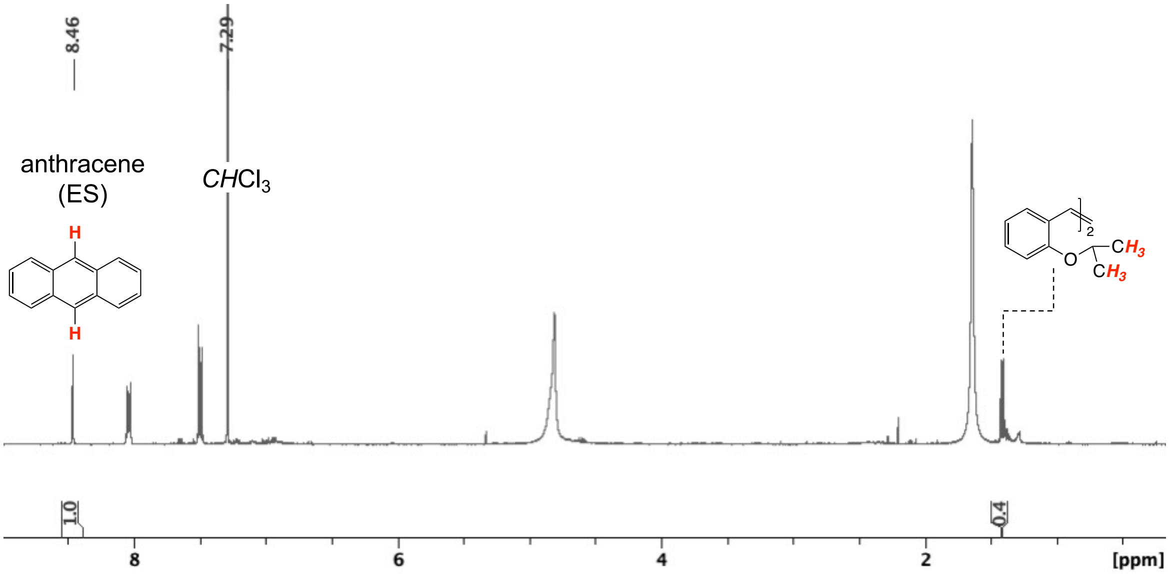


(b) GC-MS evidence for **2**

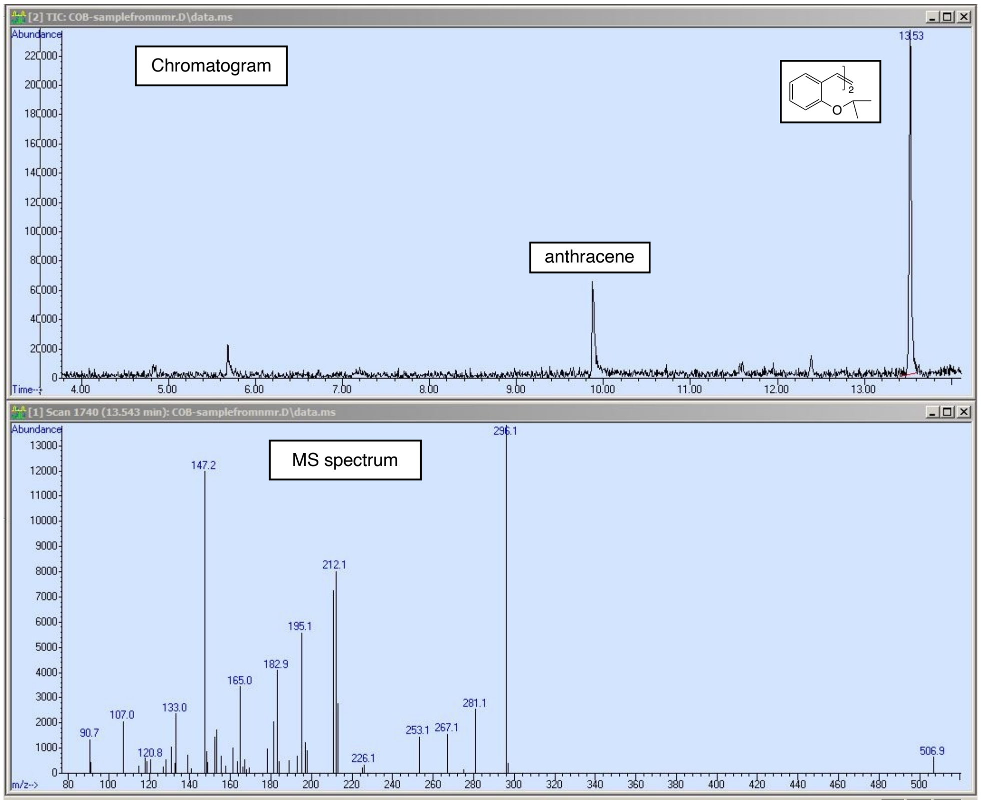


**Figure S23**. Organic products formed by decomposition of **AM** in water. (a) $^1$H NMR spectrum (300 MHz, $CDCl_3$) showing quantitation of trans-stilbenoid **2** with anthracene as external standard (ES). (b) GC-MS showing **2** as the main product (at 13.53 min) Calc'd for $C_{20}H_{24}O_2$ ($[M]^{+\cdot}$), *m/z* 296.2. Found: *m/z* 296.1.

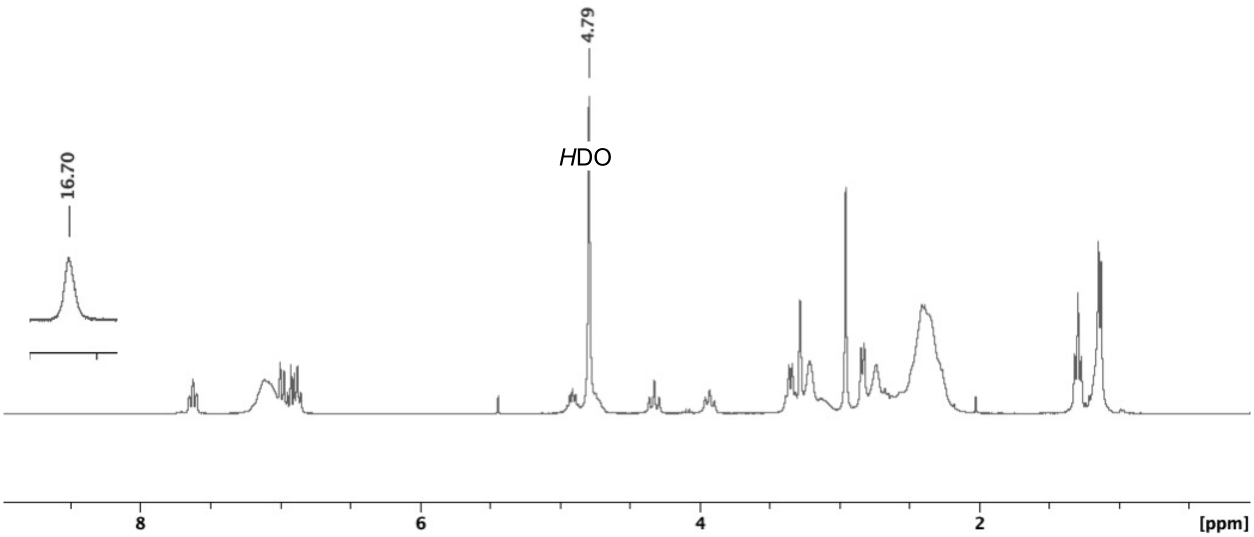


**Figure S24**. $^{1}$H NMR spectrum of **AM** (300 MHz, $D_2O$) upon addition of $CD_3OD$. The significant narrowing of the alkylidene singlet ($\omega_{0.5}$ = 20 Hz in $D_2O/CD_3OD$, vs 89 Hz in $D_2O$) implies that the initial broadening is due to dynamic processes rather than paramagnetic species.

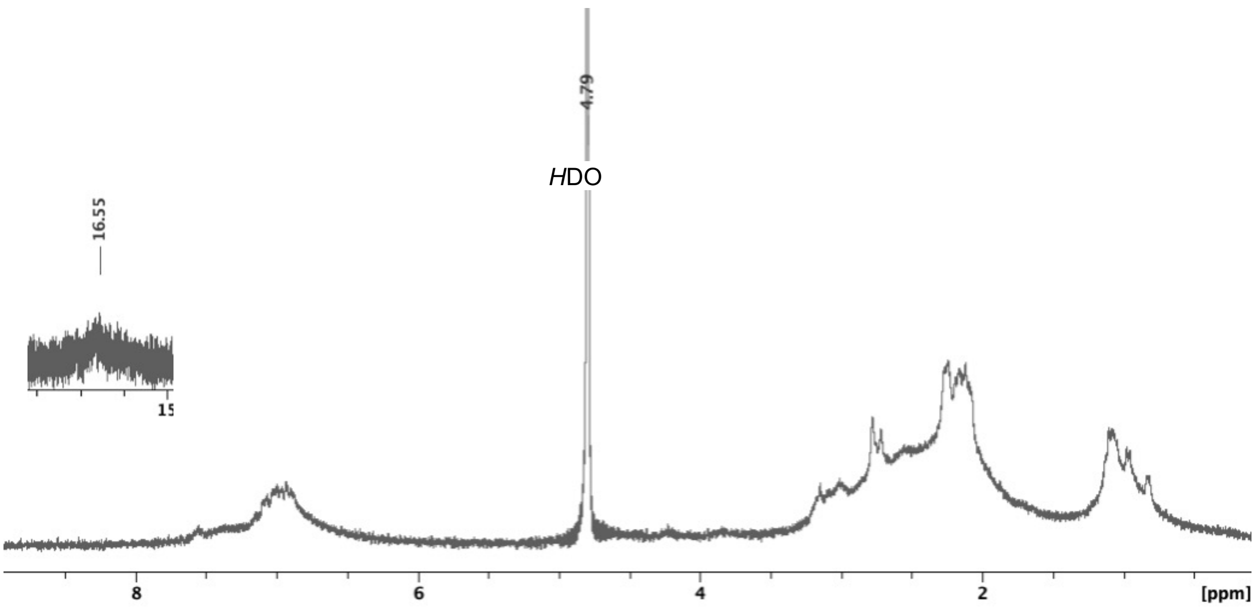


**Figure S25**. $^{1}$H NMR spectrum (300 MHz, $D_2O$) of **AM** showing poor resolution at 5 ºC.

(a) $^{1}$H NMR of decomposed **AM** showing absence of discrete signals.

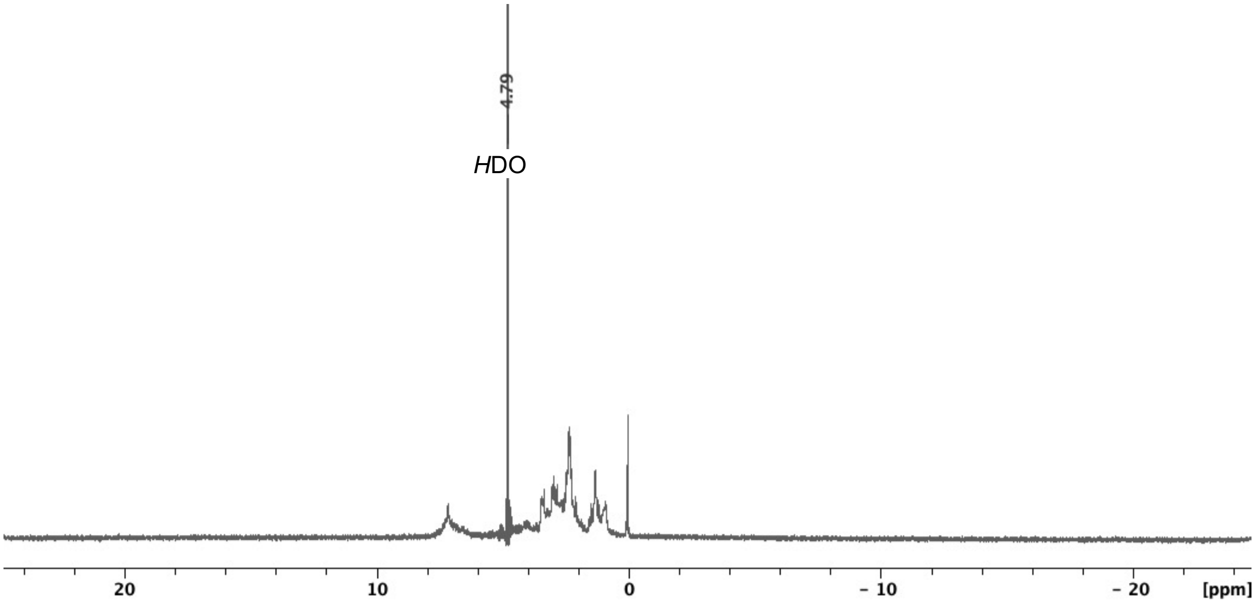


(b) $^{2}$H NMR of decomposed **AM**

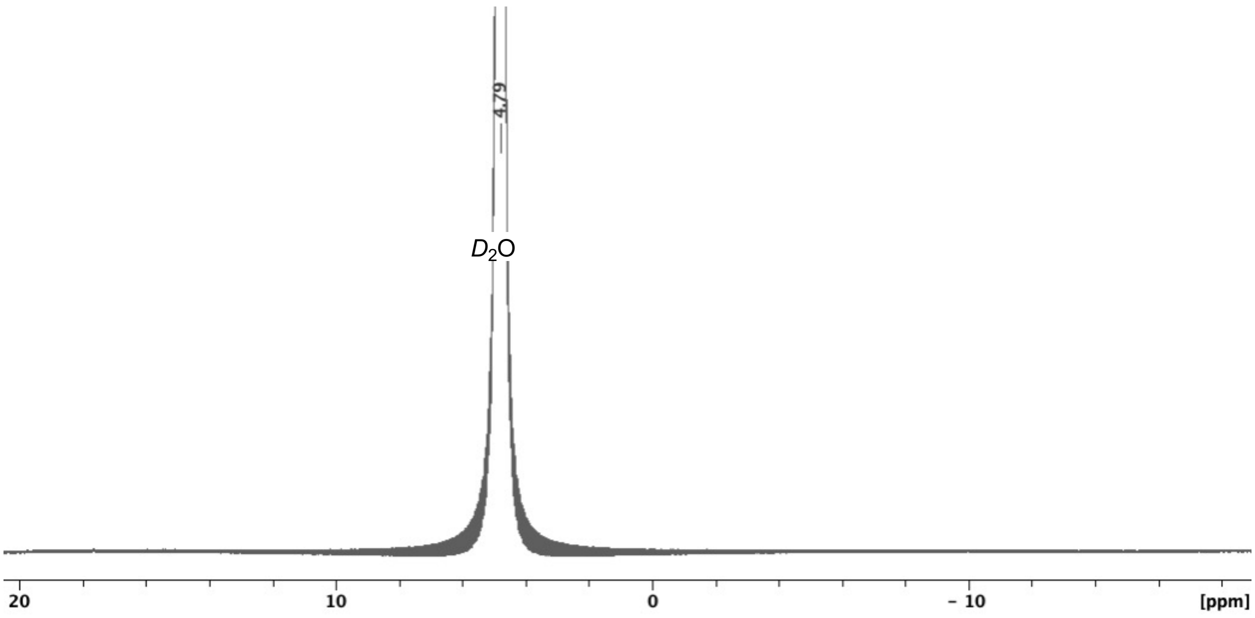


**Figure S26**. NMR analysis (300 MHz, $D_2O$) of decomposed **AM**. formation. (a) $^{1}$H NMR spectrum shows no evidence for ruthenium hydrides. (b) $^{2}$H NMR shows no deuterides.

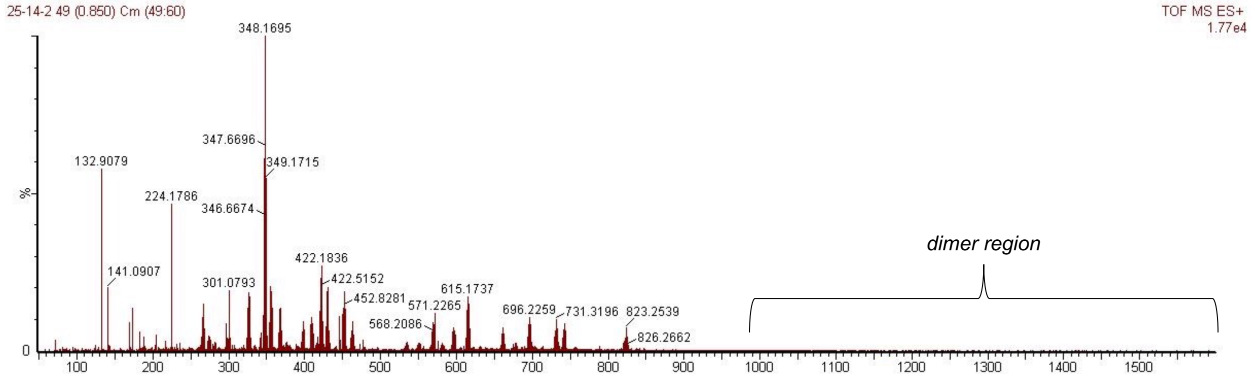


**Figure S27**. ESI-MS analysis ($H_2O$) of the decomposition products formed from **AM**, showing no evidence of ruthenium dimers.

(a) $^1$H NMR spectrum of **AM** in $CD_2Cl_2$

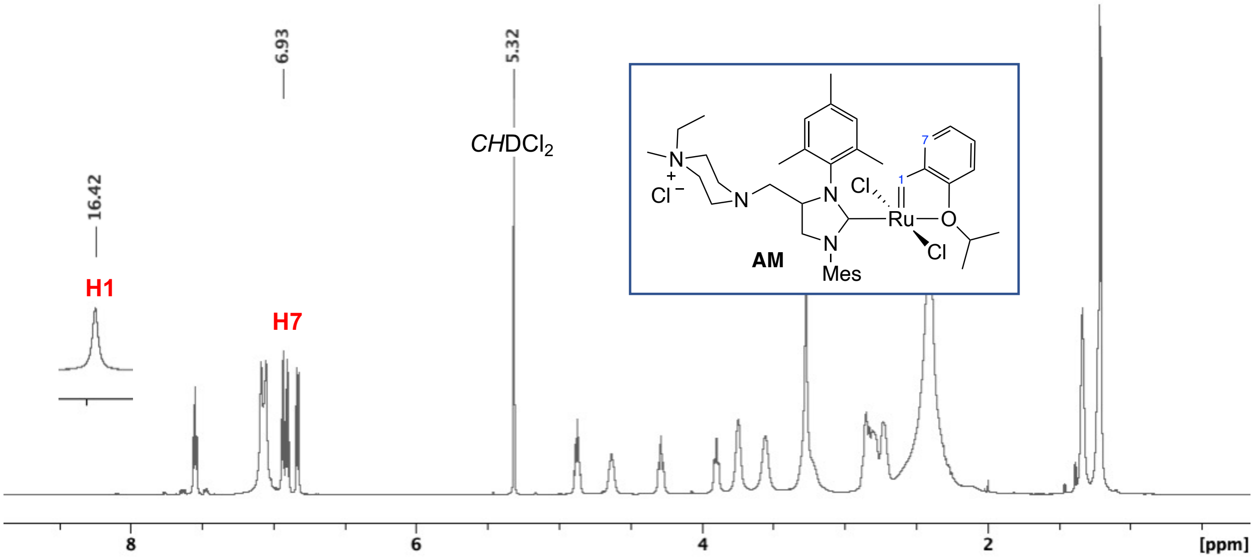


(b) $^1$H-$^{13}$C HMBC NMR of **AM** in $CD_2Cl_2$

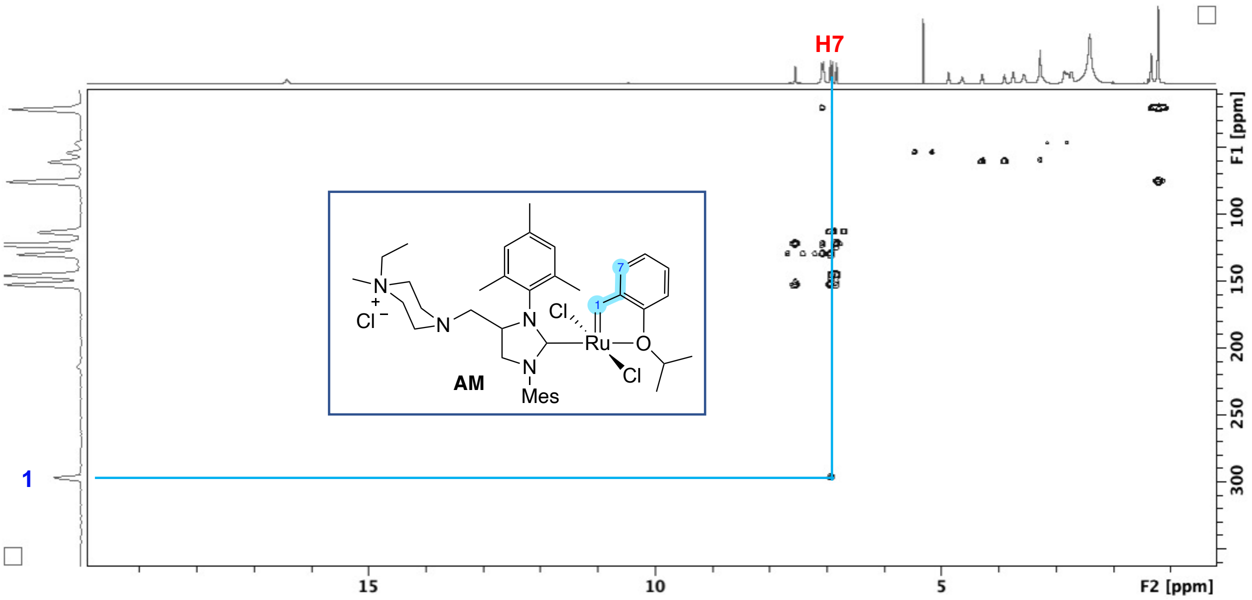


**Figure S28**. NMR spectra of **AM**. (a) $^1$H NMR spectrum (600 MHz, $CD_2Cl_2$). (b) $^1$H-$^{13}$C HMBC NMR (600/151 MHz) showing correlation between the [Ru]=C*H*Ar and benzylidene *o*-C*H* protons.

(a) $^{1}$H NMR spectrum of **AM** + 5 equiv $PCy_3$ in $CD_2Cl_2$

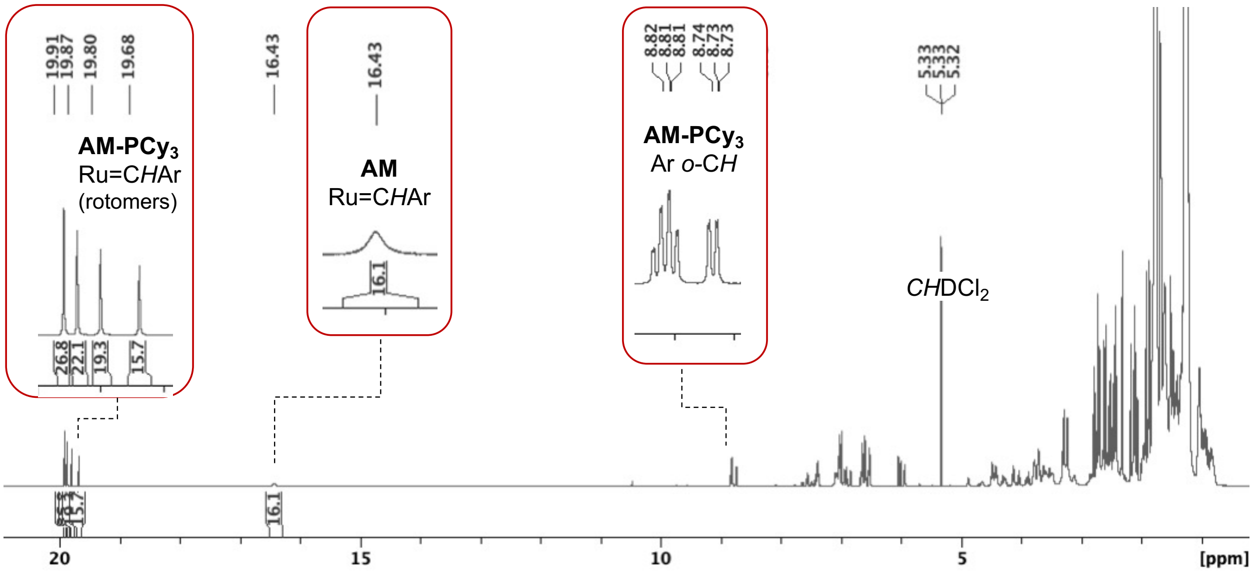


(b) $^{1}$H-$^{13}$C HMBC NMR of **AM** + 5 equiv $PCy_3$ in $CD_2Cl_2$

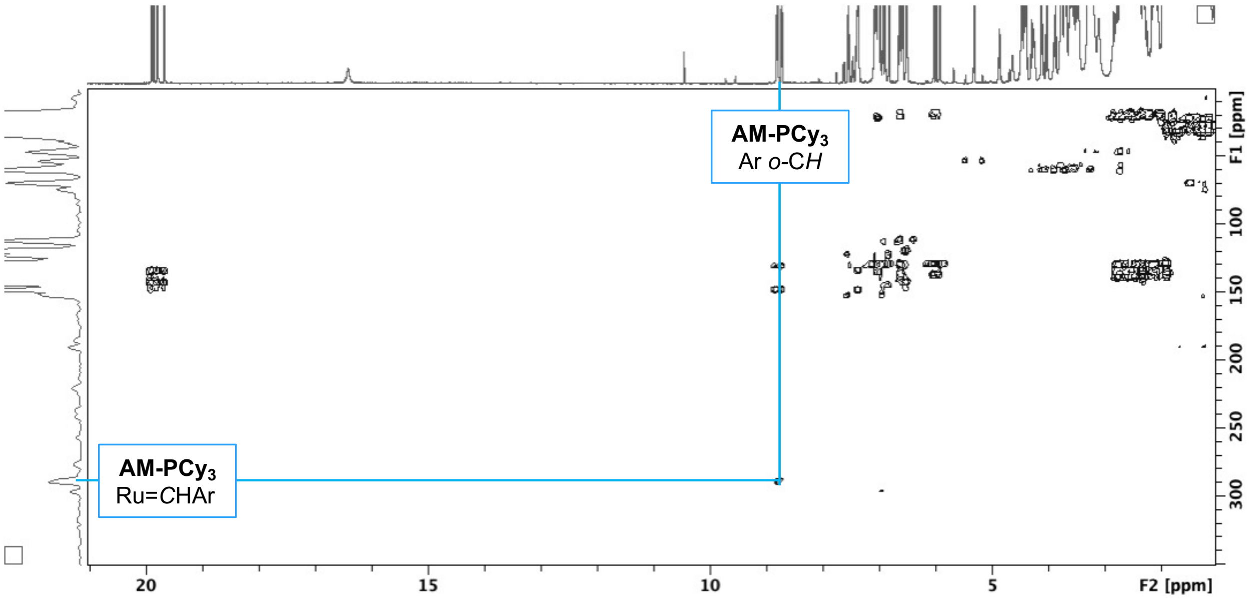


**Figure S29**. (a) $^{1}$H NMR spectrum (600 MHz, $CD_2Cl_2$) of **AM** after adding 5 equiv $PCy_3$, showing **AM**-$PCy_3$ adducts (rotamers). (b) $^{1}$H-$^{13}$C HMBC NMR spectrum (600/151 MHz) showing correlation between the [Ru]=C*H*Ar and benzylidene *o*-C*H* protons.

## S3. References.


(1) Blacquiere, J. M.; Jurca, T.; Weiss, J.; Fogg, D. E., Time as a Dimension in High-Throughput Homogeneous Catalysis. *Adv. Synth. Catal.* **2008**, *350*, 2849–2855.

(2) Escudero, J.; Bellosta, V.; Cossy, J., Rhodium-Catalyzed Cyclization of *O*,ω-Unsaturated Alkoxyamines: Formation of Oxygen-Containing Heterocycles. *Angew. Chem., Int. Ed.* **2018**, *57*, 574–578.

(3) Gans, P.; Sabatini, A.; Vacca, A., Investigation of equilibria in solution. Determination of equilibrium constants with the HYPERQUAD suite of programs. *Talanta* **1996**, *43*, 1739–1753.

(4) Gans, P.; Sabatini, A.; Vacca, A., Determination of equilibrium constants from spectrophometric data obtained from solutions of known pH: The program pHab. *Ann. Chim.* **1999**, *89*, 45–49.

(5) Kiss, T.; Enyedy, É. A.; Jakusch, T., Development of the application of speciation in chemistry. *Coord. Chem. Rev.* **2017**, *352*, 401–423.

(6) Garakani, T. M.; Sauer, D. F.; Mertens, M. A. S.; Lazar, J.; Gehrmann, J.; Arlt, M.; Schiffels, J.; Schnakenberg, U.; Okuda, J.; Schwaneberg, U., FhuA-Grubbs-Hoveyda Biohybrid Catalyst Embedded in a Polymer Film Enables Catalysis in Neat Substrates. *ACS Catal.* **2020**, *10*, 10946–10953.

(7) Gessler, S.; Randl, S.; Blechert, S., Synthesis and Metathesis Reactions of a Phosphine-Free Dihydroimidazole Carbene Ruthenium Complex. *Tetrahedron Lett.* **2000**, *41*, 9973–9976.
